# Supplementary material for: Prioritizing Parkinson’s disease risk genes in genome-wide association loci
Source: NPJ Parkinsons Dis. 2025 Apr 16;11:77. doi: 10.1038/s41531-025-00933-0 (PMC12003903; doi:10.1038/s41531-025-00933-0)
Supplement: Supplementary file 1 — Supplementary information [file 41531_2025_933_MOESM1_ESM.pdf]

Supplementary Material

Prioritizing Parkinson's disease risk genes in genome-wide association loci

Lara M. Lange[1,2,*], Catalina Cerquera-Cleves[3,4,*], Marijn Schipper[5], Georgia Panagiotaropoulou[6,7,8], Alice Braun[6,7,8], Julia Kraft[6,7,8,], Swapnil Awasthi[6,7,8], Nathaniel Bell[5], Danielle Posthuma[5,9], Stephan Ripke[6,7,8,†], Cornelis Blauwendraat[2,10,†], and Karl Heilbron[6,7,8,11,†]

[*] Denotes equal first author contribution

[†] Denotes equal last author contribution

Author affiliations

[1]Institute of Neurogenetics, University of Luebeck, Luebeck, Germany

[2]Laboratory of Neurogenetics, National Institute on Aging, Bethesda, Maryland, USA

[3]Neurology Unit, Department of Neurosciences, Hospital Universitario San Ignacio, Bogotá, Colombia.

[4]CHU de Québec Research Center, Axe Neurosciences, Laval University, Quebec City, Quebec, Canada.

[5]Vrije Universiteit Amsterdam, Amsterdam, The Netherlands

[6]Department of Psychiatry and Psychotherapy, Charité – Universitätsmedizin Berlin, Berlin, Germany

[7]Stanley Center for Psychiatric Research, Broad Institute of MIT and Harvard, Cambridge, Massachusetts, USA

[8]German Center for Mental Health (DZPG), partner site Berlin/Potsdam, Berlin, Germany

[9]Department of Child and Adolescent Psychiatry and Pediatric Psychology, Section Complex Trait Genetics, Amsterdam Neuroscience, Vrije Universiteit Medical Center, Amsterdam, The Netherlands

[10]Center for Alzheimer's and Related Dementias, National Institute on Aging and National Institute of Neurological Disorders and Stroke, National Institutes of Health, Bethesda, MD, USA

[11]Current address: Bayer AG, Research & Development, Pharmaceuticals, Berlin, Germany

Supplementary Tables

Supplementary Table 1. Independent genome-wide significant loci.

Chr	SNP	bp	refA	freq	b	se	p	n	freq_geno	bJ	bJ_se	pJ
1	rs11164870	93552187	C	0.386	0.054	0.009	2.16E-09	151259	0.386	0.054	0.009	2.16E-09
1	rs114138760	154898185	C	0.009	0.281	0.048	4.19E-09	145870	0.009	0.306	0.048	1.57E-10
1	rs35749011	155135036	A	0.011	0.607	0.034	1.72E-70	238901	0.011	0.446	0.038	9.70E-32
1	rs76763715	155205634	C	0.002	0.747	0.076	1.59E-22	251713	0.002	0.766	0.076	1.32E-23
1	rs12734374	155388851	T	0.018	0.619	0.037	3.85E-62	122193	0.018	0.415	0.041	1.50E-23
1	rs1801274	161479745	G	0.464	0.064	0.009	1.14E-11	133186	0.464	0.064	0.009	1.15E-11
1	rs10913559	171701487	T	0.315	-0.055	0.009	4.02E-09	155874	0.315	-0.055	0.009	4.04E-09
1	rs1772140	205672482	A	0.051	-0.191	0.021	2.13E-20	143902	0.051	-0.142	0.021	1.83E-11
1	rs11557080	205737739	A	0.182	0.152	0.012	6.35E-39	144495	0.182	0.110	0.013	4.23E-18
1	rs708723	205739266	C	0.448	-0.123	0.009	3.28E-44	153306	0.448	-0.077	0.010	3.82E-15
1	rs16846351	226846712	G	0.010	0.285	0.046	8.16E-10	139353	0.010	0.266	0.046	1.09E-08
1	rs4653767	226916078	C	0.301	-0.090	0.010	1.08E-20	149068	0.301	-0.088	0.010	1.42E-19
1	rs1326293	232669682	T	0.141	0.099	0.012	3.93E-16	162401	0.141	0.099	0.012	3.98E-16
2	rs76116224	18147848	T	0.088	-0.110	0.019	1.27E-08	96426.1	0.088	-0.110	0.019	1.27E-08
2	rs12613887	29111099	C	0.303	-0.058	0.011	3.98E-08	123071	0.303	-0.058	0.011	3.99E-08
2	rs398290	32503526	T	0.393	-0.052	0.009	1.92E-08	143321	0.393	-0.052	0.009	1.93E-08
2	rs9309337	61763207	T	0.233	0.056	0.010	4.24E-08	156141	0.233	0.056	0.010	4.25E-08
2	rs4581940	69655790	C	0.406	0.050	0.009	2.18E-08	152114	0.406	0.050	0.009	2.18E-08
2	rs72819488	95947099	A	0.146	-0.072	0.013	1.99E-08	140779	0.146	-0.072	0.013	2.00E-08
2	rs11683001	102396963	A	0.349	0.065	0.009	8.84E-13	157649	0.349	0.065	0.009	8.91E-13
2	rs57891859	135464616	G	0.331	-0.067	0.010	1.96E-11	134062	0.331	-0.085	0.010	1.22E-16
2	rs17698151	135543367	C	0.144	-0.077	0.013	7.01E-09	135500	0.144	-0.103	0.014	4.13E-14
2	rs4547489	161159225	G	0.451	0.051	0.009	5.43E-09	152893	0.451	0.051	0.009	5.45E-09
2	rs353128	166138608	G	0.403	-0.049	0.009	3.49E-08	152918	0.403	-0.049	0.009	3.50E-08
2	rs1474055	169110394	T	0.144	0.180	0.014	2.54E-39	126406	0.144	0.180	0.014	2.86E-39
3	rs73038319	18361759	C	0.033	0.169	0.024	5.94E-13	165866	0.033	0.169	0.024	5.98E-13
3	rs6808178	28705690	T	0.361	0.065	0.009	1.47E-12	152705	0.361	0.065	0.009	1.48E-12

3	rs12497850	48748989	G	0.326	-0.060	0.010	1.10E-09	139784	0.326	-0.060	0.010	1.10E-09
3	rs55961674	122196892	T	0.155	0.086	0.013	9.98E-12	139493	0.155	0.086	0.013	1.01E-11
3	rs6803771	151112968	A	0.346	-0.063	0.009	1.21E-11	148315	0.346	-0.063	0.009	1.37E-11
3	rs16864251	152116954	A	0.256	0.058	0.010	2.24E-08	142004	0.256	0.058	0.010	2.54E-08
3	rs1450522	161077630	G	0.339	0.055	0.009	1.35E-09	158028	0.339	0.055	0.009	1.36E-09
3	rs6806917	178861417	C	0.174	0.067	0.012	1.71E-08	145016	0.174	0.067	0.012	1.72E-08
3	rs10513789	182760073	G	0.245	-0.158	0.011	4.29E-49	137833	0.245	-0.158	0.011	5.08E-49
4	rs873786	925376	T	0.094	-0.173	0.018	1.79E-21	103991	0.094	-0.149	0.018	3.26E-16
4	rs34311866	951947	C	0.176	0.206	0.011	1.67E-73	156432	0.176	0.199	0.011	3.68E-68
4	rs4698412	15737348	G	0.479	-0.105	0.009	1.86E-33	155938	0.479	-0.105	0.009	1.99E-33
4	rs34025766	17968811	A	0.163	-0.084	0.012	8.60E-12	141739	0.163	-0.084	0.012	8.67E-12
4	rs3816248	77101068	C	0.172	-0.071	0.012	1.03E-09	153458	0.172	-0.126	0.013	5.68E-23
4	rs1465922	77134870	G	0.391	0.066	0.009	7.08E-13	144942	0.391	0.117	0.011	6.79E-26
4	rs6854006	77198054	T	0.331	-0.083	0.009	1.10E-18	147970	0.331	-0.088	0.010	6.08E-19
4	rs356183	90626098	G	0.463	0.241	0.010	2.50E-140	128314	0.463	0.162	0.014	2.03E-32
4	rs356203	90666041	C	0.390	0.256	0.009	2.82E-183	155950	0.390	0.198	0.012	8.13E-57
4	rs3910105	90682571	G	0.442	-0.224	0.024	6.63E-21	20781.5	0.442	-0.232	0.027	5.76E-18
4	rs2619356	90750844	C	0.207	-0.346	0.035	5.57E-23	14371.1	0.207	-0.291	0.038	1.14E-14
4	rs13117519	114369065	T	0.165	0.085	0.012	4.25E-13	154093	0.165	0.085	0.012	4.28E-13
4	rs62333164	170583157	A	0.307	-0.066	0.010	2.51E-11	142702	0.307	-0.066	0.010	2.53E-11
5	rs4546327	60177302	T	0.090	0.152	0.016	2.52E-22	146405	0.090	0.152	0.016	2.59E-22
5	rs246815	75599176	C	0.082	0.095	0.016	1.25E-09	158952	0.082	0.095	0.016	1.26E-09
5	rs66872803	79612283	A	0.134	-0.076	0.014	3.78E-08	132803	0.134	-0.076	0.014	3.79E-08
5	rs26431	102365794	G	0.322	-0.055	0.009	4.22E-09	151998	0.322	-0.055	0.009	4.24E-09
5	rs11950533	134199105	A	0.124	-0.082	0.014	1.50E-09	145241	0.124	-0.082	0.014	1.50E-09
6	rs4140646	27738801	A	0.219	0.084	0.011	3.19E-14	139653	0.219	0.082	0.011	1.52E-13
6	rs3132453	31604044	T	0.065	0.112	0.018	8.33E-10	146592	0.065	0.107	0.018	3.77E-09
6	rs112485576	32578772	A	0.158	-0.167	0.014	9.63E-31	105132	0.158	-0.216	0.017	2.70E-37
6	rs12528068	72487762	T	0.254	0.069	0.010	4.09E-12	154730	0.254	0.069	0.010	4.12E-12
6	rs9487736	112168309	A	0.157	-0.092	0.012	9.95E-15	155338	0.157	-0.092	0.012	1.01E-14
6	rs41286192	133118216	G	0.031	0.243	0.032	6.29E-14	94231.7	0.031	0.243	0.032	6.40E-14
7	rs199351	23300049	C	0.411	-0.097	0.009	1.37E-27	150894	0.411	-0.097	0.009	1.44E-27

7	rs76949143	66009851	A	0.062	-0.120	0.020	1.57E-09	128748	0.062	-0.120	0.020	1.58E-09
8	rs1293298	11712443	C	0.229	-0.093	0.011	3.99E-16	127019	0.229	-0.093	0.011	4.06E-16
8	rs34096562	16701281	G	0.302	-0.085	0.010	9.36E-19	150522	0.302	-0.085	0.010	9.55E-19
8	rs3736147	22471824	A	0.314	0.057	0.009	5.65E-10	158433	0.314	0.057	0.009	5.68E-10
9	rs13294100	17579690	T	0.377	-0.083	0.009	2.05E-19	147617	0.377	-0.078	0.009	3.97E-17
9	rs10756905	17726888	T	0.243	0.092	0.010	1.43E-20	160986	0.243	0.087	0.010	2.77E-18
9	rs6476434	34046391	C	0.255	0.065	0.010	7.66E-11	153190	0.255	0.065	0.010	7.70E-11
10	rs878321	15557115	G	0.342	-0.073	0.010	4.55E-13	128289	0.342	-0.073	0.010	4.60E-13
10	rs10748818	104015279	G	0.163	0.070	0.011	5.19E-10	166735	0.163	0.070	0.011	5.21E-10
10	rs72840788	121415685	A	0.195	0.076	0.011	1.57E-11	145700	0.195	0.083	0.011	2.22E-13
10	rs117896735	121536327	A	0.015	0.435	0.039	2.36E-28	131635	0.015	0.451	0.040	3.69E-30
11	rs4910149	10533056	A	0.127	-0.089	0.013	6.02E-12	156262	0.127	-0.089	0.013	6.06E-12
11	rs12283611	83487277	A	0.402	-0.072	0.010	4.89E-14	134813	0.402	-0.072	0.010	4.94E-14
11	rs2156675	133707509	G	0.206	0.104	0.011	6.88E-20	137509	0.206	0.104	0.011	7.06E-20
12	rs140427697	32491673	G	0.008	0.298	0.047	2.54E-10	163784	0.008	0.298	0.047	2.55E-10
12	rs814228	40383902	C	0.337	0.088	0.009	1.79E-21	151802	0.337	0.079	0.010	1.20E-16
12	rs141336855	40387749	T	0.002	0.689	0.068	2.97E-24	281617	0.002	0.668	0.068	9.29E-23
12	rs28370650	40399948	A	0.017	0.366	0.030	9.78E-35	195057	0.017	0.417	0.031	3.90E-41
12	rs7134524	40453281	A	0.148	0.406	0.054	3.41E-14	8044.77	0.148	0.347	0.054	1.42E-10
12	rs17442721	40535773	G	0.016	0.403	0.039	1.77E-24	123106	0.016	0.597	0.054	2.00E-28
12	rs4272849	40670801	T	0.456	0.057	0.009	4.85E-11	154864	0.456	0.112	0.010	6.78E-29
12	rs144296031	41001985	C	0.013	0.391	0.039	3.76E-24	157597	0.013	0.340	0.044	6.01E-15
12	rs11179174	41347387	A	0.022	0.238	0.030	2.82E-15	148526	0.022	0.260	0.030	1.05E-17
12	rs7134559	46419086	T	0.410	-0.056	0.009	5.65E-10	148291	0.410	-0.056	0.009	5.68E-10
12	rs10847864	123326598	T	0.367	0.134	0.010	3.25E-38	116374	0.367	0.134	0.010	3.66E-38
12	rs11610045	133063768	A	0.449	0.063	0.009	4.05E-12	143832	0.449	0.063	0.009	4.08E-12
13	rs9535211	49939903	T	0.284	-0.061	0.011	3.82E-08	117297	0.284	-0.061	0.011	3.84E-08
13	rs6491345	97915358	C	0.345	0.059	0.010	1.30E-08	120244	0.345	0.059	0.010	1.30E-08
14	rs8005136	37995936	T	0.386	-0.057	0.009	1.11E-09	143612	0.386	-0.057	0.009	1.11E-09
14	rs11158026	55348869	T	0.357	-0.077	0.009	2.11E-16	146706	0.357	-0.077	0.009	2.14E-16
14	rs3742785	75373034	C	0.218	-0.072	0.011	4.18E-11	145991	0.218	-0.072	0.011	4.21E-11
14	rs2008686	88485777	G	0.476	0.061	0.009	1.33E-11	145783	0.476	0.061	0.009	1.34E-11

15	rs28648524	50787409	T	0.244	-0.061	0.011	3.07E-08	130011	0.244	-0.061	0.011	3.08E-08
15	rs2251086	61997385	T	0.148	-0.120	0.013	1.70E-21	146085	0.148	-0.120	0.013	1.75E-21
16	rs6497339	19277493	A	0.399	0.063	0.009	2.76E-11	136411	0.399	0.063	0.009	2.77E-11
16	rs2904880	28944396	C	0.299	-0.065	0.011	7.87E-10	124850	0.299	-0.065	0.011	7.91E-10
16	rs11150601	30977799	G	0.331	-0.091	0.010	4.15E-21	142428	0.331	-0.091	0.010	4.26E-21
16	rs6500328	50736656	G	0.382	-0.059	0.009	1.44E-10	145986	0.382	-0.059	0.009	1.45E-10
16	rs3104783	52636242	A	0.472	0.069	0.009	1.01E-14	147709	0.472	0.066	0.009	2.20E-13
16	rs10221156	52969426	A	0.086	-0.108	0.017	3.24E-10	126686	0.086	-0.100	0.017	7.23E-09
17	rs9217	7363088	C	0.343	0.055	0.010	2.03E-08	137081	0.343	0.055	0.010	2.04E-08
17	rs178654	16064716	A	0.465	0.053	0.010	4.64E-08	127485	0.465	0.053	0.010	4.66E-08
17	rs12944773	36136832	C	0.295	-0.184	0.028	6.78E-11	17589.6	0.295	-0.184	0.028	7.14E-11
17	rs665268	40722029	G	0.293	-0.064	0.011	1.54E-09	125771	0.293	-0.064	0.011	1.55E-09
17	rs10208	42300278	T	0.366	-0.063	0.010	4.71E-11	135892	0.366	-0.065	0.010	2.58E-11
17	rs35941271	42436220	A	0.092	0.107	0.019	9.47E-09	100009	0.092	0.107	0.019	9.87E-09
17	rs62053943	43744203	T	0.134	-0.270	0.015	3.58E-68	105229	0.134	-0.270	0.017	9.89E-59
17	rs11652924	44182267	T	0.422	0.106	0.010	1.42E-27	127549	0.422	0.092	0.011	5.97E-18
17	rs12951057	44794558	G	0.135	0.081	0.015	4.00E-08	115050	0.135	0.100	0.016	1.70E-10
17	rs112857769	59931916	G	0.207	0.080	0.014	4.23E-09	96890	0.207	0.080	0.014	4.25E-09
17	rs8071516	76416825	A	0.156	-0.075	0.013	6.05E-09	134185	0.156	-0.075	0.013	6.08E-09
18	rs8091977	31359414	T	0.481	-0.057	0.009	8.67E-11	151406	0.481	-0.057	0.009	8.72E-11
18	rs12456492	40673380	G	0.320	0.102	0.009	4.76E-29	161752	0.320	0.102	0.009	4.99E-29
19	rs55818311	2341047	C	0.352	0.070	0.011	4.18E-10	103315	0.352	0.070	0.011	4.21E-10
20	rs2295545	3164686	C	0.422	-0.062	0.010	8.45E-11	130730	0.422	-0.062	0.010	8.51E-11
20	rs111593423	6008226	C	0.112	0.079	0.014	7.89E-09	157941	0.112	0.079	0.014	7.91E-09
20	rs1413146	46407290	C	0.439	-0.053	0.009	1.69E-08	136729	0.439	-0.053	0.009	1.70E-08
21	rs11701722	38770530	C	0.283	0.067	0.010	9.66E-12	151467	0.283	0.067	0.010	9.73E-12
21	rs2837424	41452034	T	0.186	0.063	0.011	3.80E-08	146349	0.186	0.063	0.011	3.81E-08
22	rs9611522	41627924	T	0.235	-0.060	0.011	4.22E-08	138082	0.235	-0.060	0.011	4.23E-08
22	rs10775809	50808017	T	0.112	-0.092	0.016	6.68E-09	117406	0.112	-0.092	0.016	6.71E-09

Chr = chromosome; SNP = rsID; bp = base pairs (physical position); refA = effect allele; freq = frequency of the effect allele in the genome-wide association study; b = marginal log odds ratio; se = marginal standard error; p = marginal p value; n = effective sample size; freq_geno = frequency of the effect allele in the reference panel, bJ = conditional effect size; bJ_se = conditional standard error; pJ = conditional P value

Supplementary Table 2. Gene prioritization results for all genes in all loci.

Prioritized	Locus	rsID	Gene	ENSGID	Best Distance	Distance	Best PoPS	PoPS quantile	PoPS value	Non-synonymous PIP	# genes	L2G	Yu2024
FALSE	1	rs11164870	CCDC18	ENSG00000122483	TRUE	0	FALSE	0.885	0.254		9		
FALSE	1	rs11164870	TMED5	ENSG00000117500	FALSE	49334	TRUE	0.979	0.465	0.01528346	9		
FALSE	1	rs11164870	MTF2	ENSG00000143033	FALSE	90771	FALSE	0.930	0.315		9		
FALSE	1	rs11164870	DR1	ENSG00000117505	FALSE	116057	FALSE	0.923	0.306		9		
FALSE	1	rs11164870	FNBP1L	ENSG00000137942	FALSE	218268	FALSE	0.866	0.234		9		
FALSE	1	rs11164870	DIPK1A	ENSG00000154511	FALSE	268328	FALSE	0.969	0.420		9		
FALSE	1	rs11164870	BCAR3	ENSG00000137936	FALSE	331891	FALSE	0.257	-0.127		9		
FALSE	1	rs11164870	RPL5	ENSG00000122406	FALSE	387926	FALSE	0.825	0.199		9		
FALSE	1	rs11164870	EVI5	ENSG00000067208	FALSE	437446	FALSE	0.848	0.218		9		
FALSE	2	rs114138760	PMVK	ENSG00000163344	TRUE	0	FALSE	0.955	0.369		25		0.026196038
FALSE	2	rs114138760	PBXIP1	ENSG00000163346	FALSE	18367	FALSE	0.243	-0.135		25		0.020343689
FALSE	2	rs114138760	PYGO2	ENSG00000163348	FALSE	31317	FALSE	0.922	0.304		25		0.004409654
FALSE	2	rs114138760	SHC1	ENSG00000160691	FALSE	36589	FALSE	0.966	0.405		25		0.010275714
FALSE	2	rs114138760	CKS1B	ENSG00000173207	FALSE	48944	FALSE	0.166	-0.182		25		0.017501485
FALSE	2	rs114138760	KCNN3	ENSG00000143603	FALSE	55428	FALSE	0.893	0.265		25		0.023060345
FALSE	2	rs114138760	FLAD1	ENSG00000160688	FALSE	57629	FALSE	0.630	0.073		25		0.026615797
FALSE	2	rs114138760	LENEP	ENSG00000163352	FALSE	67877	FALSE	0.391	-0.056		25		0.008286151
FALSE	2	rs114138760	ZBTB7B	ENSG00000160685	FALSE	76921	FALSE	0.985	0.508		25		0.007108425
FALSE	2	rs114138760	DCST2	ENSG00000163354	FALSE	92811	FALSE	0.487	-0.004		25		0.008814173
FALSE	2	rs114138760	DCST1	ENSG00000163357	FALSE	108115	FALSE	0.277	-0.116		25		0.019215236
FALSE	2	rs114138760	ADAM15	ENSG00000143537	FALSE	124857	TRUE	0.997	0.792		25		0.021091538
FALSE	2	rs114138760	EFNA4	ENSG00000243364	FALSE	138028	FALSE	0.429	-0.036		25		0.026980925
FALSE	2	rs114138760	EFNA4-EFNA3	ENSG00000251246	FALSE	138039	FALSE	0.367	-0.069		25		
FALSE	2	rs114138760	EFNA3	ENSG00000143590	FALSE	153128	FALSE	0.966	0.405		25		0.024859459
FALSE	2	rs114138760	EFNA1	ENSG00000169242	FALSE	202167	FALSE	0.899	0.272		25		0.002848349
FALSE	2	rs114138760	SLC50A1	ENSG00000169241	FALSE	209635	FALSE	0.960	0.386		25		0.006091254

FALSE	2	rs114138760	DPM3	ENSG00000179085	FALSE	214182	FALSE	0.938	0.330	25	0.014921706
FALSE	2	rs114138760	KRTCAP2	ENSG00000163463	FALSE	243699	FALSE	0.614	0.064	25	0.021273074
FALSE	2	rs114138760	ENSG00000273088	ENSG00000273088	FALSE	243700	FALSE	NA	NA	25	
FALSE	2	rs114138760	TRIM46	ENSG00000163462	FALSE	248078	FALSE	0.865	0.234	25	0.009067585
FALSE	2	rs114138760	MUC1	ENSG00000185499	FALSE	260115	FALSE	0.983	0.488	25	0.026984681
FALSE	2	rs114138760	THBS3	ENSG00000169231	FALSE	267194	FALSE	0.852	0.222	25	0.023446951
FALSE	2	rs114138760	MTX1	ENSG00000173171	FALSE	280301	FALSE	0.540	0.024	25	0.024108849
FALSE	2	rs114138760	ADAR	ENSG00000160710	FALSE	297696	FALSE	0.713	0.121	25	0.021128588
FALSE	3	rs35749011	KRTCAP2	ENSG00000163463	TRUE	6848	FALSE	0.614	0.064	33	0.021273074
FALSE	3	rs35749011	ENSG00000273088	ENSG00000273088	FALSE	6849	FALSE	NA	NA	33	
FALSE	3	rs35749011	TRIM46	ENSG00000163462	FALSE	11227	FALSE	0.865	0.234	33	0.009067585
FALSE	3	rs35749011	DPM3	ENSG00000179085	FALSE	21965	FALSE	0.938	0.330	33	0.014921706
FALSE	3	rs35749011	MUC1	ENSG00000185499	FALSE	23264	FALSE	0.983	0.488	33	0.026984681
FALSE	3	rs35749011	SLC50A1	ENSG00000169241	FALSE	23703	FALSE	0.960	0.386	33	0.006091254
FALSE	3	rs35749011	EFNA1	ENSG00000169242	FALSE	27661	FALSE	0.899	0.272	33	0.002848349
FALSE	3	rs35749011	THBS3	ENSG00000169231	FALSE	30343	FALSE	0.852	0.222	33	0.023446951
FALSE	3	rs35749011	MTX1	ENSG00000173171	FALSE	43450	FALSE	0.540	0.024	33	0.024108849
FALSE	3	rs35749011	GBA1	ENSG00000177628	FALSE	69207	FALSE	0.918	0.298	33	0.026320731
FALSE	3	rs35749011	EFNA3	ENSG00000143590	FALSE	75022	FALSE	0.966	0.405	33	0.024859459
FALSE	3	rs35749011	EFNA4-EFNA3	ENSG00000251246	FALSE	75753	FALSE	0.367	-0.069	33	
FALSE	3	rs35749011	ENTREP3	ENSG00000160767	FALSE	81960	FALSE	0.953	0.367	33	
FALSE	3	rs35749011	SCAMP3	ENSG00000116521	FALSE	90734	FALSE	0.974	0.440	33	0.00638
FALSE	3	rs35749011	EFNA4	ENSG00000243364	FALSE	93007	FALSE	0.429	-0.036	33	0.026980925
FALSE	3	rs35749011	CLK2	ENSG00000176444	FALSE	97623	FALSE	0.818	0.194	33	0.007254626
FALSE	3	rs35749011	ADAM15	ENSG00000143537	FALSE	99785	TRUE	0.997	0.792	33	0.021091538
FALSE	3	rs35749011	DCST1	ENSG00000163357	FALSE	111630	FALSE	0.277	-0.116	33	0.019215236
FALSE	3	rs35749011	HCN3	ENSG00000143630	FALSE	112218	FALSE	0.608	0.060	33	0.009357064
FALSE	3	rs35749011	PKLR	ENSG00000143627	FALSE	124048	FALSE	0.167	-0.182	33	0.005748648
FALSE	3	rs35749011	DCST2	ENSG00000163354	FALSE	128779	FALSE	0.487	-0.004	33	0.008814173

FALSE	3	rs35749011	FDPS	ENSG00000160752	FALSE	143503	FALSE	0.355	-0.075		33	0.017615313
FALSE	3	rs35749011	ZBTB7B	ENSG00000160685	FALSE	144037	FALSE	0.985	0.508		33	0.007108425
FALSE	3	rs35749011	RUSC1	ENSG00000160753	FALSE	155649	FALSE	0.956	0.374		33	0.026583843
FALSE	3	rs35749011	LENEP	ENSG00000163352	FALSE	168245	FALSE	0.391	-0.056		33	0.008286151
FALSE	3	rs35749011	FLAD1	ENSG00000160688	FALSE	169449	FALSE	0.630	0.073		33	0.026615797
FALSE	3	rs35749011	ASH1L	ENSG00000116539	FALSE	170023	FALSE	0.898	0.271		33	0.004017221
FALSE	3	rs35749011	CKS1B	ENSG00000173207	FALSE	183309	FALSE	0.166	-0.182		33	0.017501485
FALSE	3	rs35749011	SHC1	ENSG00000160691	FALSE	188165	FALSE	0.966	0.405		33	0.010275714
FALSE	3	rs35749011	PYGO2	ENSG00000163348	FALSE	198707	FALSE	0.922	0.304		33	0.004409654
FALSE	3	rs35749011	PBXIP1	ENSG00000163346	FALSE	206437	FALSE	0.243	-0.135		33	0.020343689
FALSE	3	rs35749011	PMVK	ENSG00000163344	FALSE	225841	FALSE	0.955	0.369		33	0.026196038
FALSE	3	rs35749011	KCNN3	ENSG00000143603	FALSE	292279	FALSE	0.893	0.265		33	0.023060345
TRUE	4	rs76763715	GBA1	ENSG00000177628	TRUE	0	FALSE	0.918	0.298	1	32	0.026320731
FALSE	4	rs76763715	ENTREP3	ENSG00000160767	FALSE	11362	FALSE	0.953	0.367		32	
FALSE	4	rs76763715	SCAMP3	ENSG00000116521	FALSE	20136	FALSE	0.974	0.440		32	0.00638
FALSE	4	rs76763715	MTX1	ENSG00000173171	FALSE	22019	FALSE	0.540	0.024		32	0.024108849
FALSE	4	rs76763715	THBS3	ENSG00000169231	FALSE	26792	FALSE	0.852	0.222		32	0.023446951
FALSE	4	rs76763715	CLK2	ENSG00000176444	FALSE	27025	FALSE	0.818	0.194		32	0.007254626
FALSE	4	rs76763715	HCN3	ENSG00000143630	FALSE	41620	FALSE	0.608	0.060		32	0.009357064
FALSE	4	rs76763715	MUC1	ENSG00000185499	FALSE	42927	FALSE	0.983	0.488		32	0.026984681
FALSE	4	rs76763715	ENSG00000273088	ENSG00000273088	FALSE	45886	FALSE	NA	NA		32	
FALSE	4	rs76763715	TRIM46	ENSG00000163462	FALSE	48187	FALSE	0.865	0.234		32	0.009067585
FALSE	4	rs76763715	PKLR	ENSG00000143627	FALSE	53450	FALSE	0.167	-0.182		32	0.005748648
FALSE	4	rs76763715	KRTCAP2	ENSG00000163463	FALSE	59683	FALSE	0.614	0.064		32	0.021273074
FALSE	4	rs76763715	FDPS	ENSG00000160752	FALSE	72905	FALSE	0.355	-0.075		32	0.017615313
FALSE	4	rs76763715	RUSC1	ENSG00000160753	FALSE	85051	FALSE	0.956	0.374		32	0.026583843
FALSE	4	rs76763715	DPM3	ENSG00000179085	FALSE	92563	FALSE	0.938	0.330		32	0.014921706
FALSE	4	rs76763715	SLC50A1	ENSG00000169241	FALSE	94301	FALSE	0.960	0.386		32	0.006091254
FALSE	4	rs76763715	EFNA1	ENSG00000169242	FALSE	98259	FALSE	0.899	0.272		32	0.002848349
FALSE	4	rs76763715	ASH1L	ENSG00000116539	FALSE	99425	FALSE	0.898	0.271		32	0.004017221
FALSE	4	rs76763715	EFNA3	ENSG00000143590	FALSE	145620	FALSE	0.966	0.405		32	0.024859459
FALSE	4	rs76763715	EFNA4-EFNA3	ENSG00000251246	FALSE	146351	FALSE	0.367	-0.069		32	
FALSE	4	rs76763715	EFNA4	ENSG00000243364	FALSE	163605	FALSE	0.429	-0.036		32	0.026980925
FALSE	4	rs76763715	ADAM15	ENSG00000143537	FALSE	170383	TRUE	0.997	0.792		32	0.021091538
FALSE	4	rs76763715	DCST1	ENSG00000163357	FALSE	182228	FALSE	0.277	-0.116		32	0.019215236

FALSE	4	rs76763715	DCST2	ENSG00000163354	FALSE	199377	FALSE	0.487	-0.004		32		0.008814173
FALSE	4	rs76763715	ZBTB7B	ENSG00000160685	FALSE	214635	FALSE	0.985	0.508		32		0.007108425
FALSE	4	rs76763715	LENEP	ENSG00000163352	FALSE	238843	FALSE	0.391	-0.056		32		0.008286151
FALSE	4	rs76763715	FLAD1	ENSG00000160688	FALSE	240047	FALSE	0.630	0.073		32		0.026615797
FALSE	4	rs76763715	CKS1B	ENSG00000173207	FALSE	253907	FALSE	0.166	-0.182		32		0.017501485
FALSE	4	rs76763715	SHC1	ENSG00000160691	FALSE	258763	FALSE	0.966	0.405		32		0.010275714
FALSE	4	rs76763715	PYGO2	ENSG00000163348	FALSE	269305	FALSE	0.922	0.304		32		0.004409654
FALSE	4	rs76763715	PBXIP1	ENSG00000163346	FALSE	277035	FALSE	0.243	-0.135		32		0.020343689
FALSE	4	rs76763715	PMVK	ENSG00000163344	FALSE	296439	FALSE	0.955	0.369		32		0.026196038
FALSE	5	rs12734374	ASH1L	ENSG00000116539	TRUE	0	FALSE	0.898	0.271		21		0.004017221
FALSE	5	rs12734374	RUSC1	ENSG00000160753	FALSE	87946	FALSE	0.956	0.374		21		0.026583843
FALSE	5	rs12734374	FDPS	ENSG00000160752	FALSE	98394	FALSE	0.355	-0.075		21		0.017615313
FALSE	5	rs12734374	PKLR	ENSG00000143627	FALSE	117622	FALSE	0.167	-0.182		21		0.005748648
FALSE	5	rs12734374	HCN3	ENSG00000143630	FALSE	129212	FALSE	0.608	0.060		21		0.009357064
FALSE	5	rs12734374	CLK2	ENSG00000176444	FALSE	140569	FALSE	0.818	0.194		21		0.007254626
FALSE	5	rs12734374	MSTO1	ENSG00000125459	FALSE	144175	FALSE	0.726	0.129		21		0.002901544
FALSE	5	rs12734374	SCAMP3	ENSG00000116521	FALSE	156630	FALSE	0.974	0.440		21		0.00638
FALSE	5	rs12734374	ENTREP3	ENSG00000160767	FALSE	163577	FALSE	0.953	0.367		21		
FALSE	5	rs12734374	GBA1	ENSG00000177628	FALSE	174361	FALSE	0.918	0.298		21		0.026320731
FALSE	5	rs12734374	MTX1	ENSG00000173171	FALSE	205236	FALSE	0.540	0.024		21		0.024108849
FALSE	5	rs12734374	THBS3	ENSG00000169231	FALSE	210009	FALSE	0.852	0.222		21		0.023446951
FALSE	5	rs12734374	MUC1	ENSG00000185499	FALSE	226144	TRUE	0.983	0.488		21		0.026984681
FALSE	5	rs12734374	ENSG00000273088	ENSG00000273088	FALSE	229103	FALSE	NA	NA		21		
FALSE	5	rs12734374	TRIM46	ENSG00000163462	FALSE	231404	FALSE	0.865	0.234		21		0.009067585
FALSE	5	rs12734374	YY1AP1	ENSG00000163374	FALSE	240383	FALSE	0.748	0.144		21		0.025975387
FALSE	5	rs12734374	KRTCAP2	ENSG00000163463	FALSE	242900	FALSE	0.614	0.064		21		0.021273074
FALSE	5	rs12734374	DAP3	ENSG00000132676	FALSE	268900	FALSE	0.829	0.201		21		0.016619417
FALSE	5	rs12734374	DPM3	ENSG00000179085	FALSE	275780	FALSE	0.938	0.330		21		0.014921706
FALSE	5	rs12734374	SLC50A1	ENSG00000169241	FALSE	277518	FALSE	0.960	0.386		21		0.006091254
FALSE	5	rs12734374	EFNA1	ENSG00000169242	FALSE	281476	FALSE	0.899	0.272		21		0.002848349
TRUE	6	rs1801274	FCGR2A	ENSG00000143226	TRUE	0	FALSE	0.669	0.095	0.707090025	21	0.663260759	0.031247052
FALSE	6	rs1801274	HSPA6	ENSG00000173110	FALSE	16587	FALSE	0.941	0.338		21	0.026875743	0.006680178
FALSE	6	rs1801274	FCGR3A	ENSG00000203747	FALSE	33806	FALSE	0.218	-0.149		21	0.020990759	0.008525668
FALSE	6	rs1801274	ENSG00000289768	ENSG00000289768	FALSE	36854	FALSE	NA	NA		21		
FALSE	6	rs1801274	FCGR2C	ENSG00000244682	FALSE	73386	FALSE	NA	NA		21		
FALSE	6	rs1801274	FCGR3B	ENSG00000162747	FALSE	115243	FALSE	0.321	-0.092		21	0.015777342	0.031842942

FALSE	6	rs1801274	CFAP126	ENSG00000188931	FALSE	140077	FALSE	0.123	-0.217		21	0.014839058	
FALSE	6	rs1801274	SDHC	ENSG00000143252	FALSE	144747	FALSE	0.747	0.144		21	0.010874679	0.006883231
FALSE	6	rs1801274	FCGR2B	ENSG00000072694	FALSE	155190	FALSE	0.911	0.288		21	0.01642173	0.004066405
FALSE	6	rs1801274	MPZ	ENSG00000158887	FALSE	197985	FALSE	0.699	0.112		21	0.012433841	0.006200594
FALSE	6	rs1801274	FCRLA	ENSG00000132185	FALSE	199019	FALSE	0.303	-0.102		21	0.011381998	0.038192966
FALSE	6	rs1801274	FCRLB	ENSG00000162746	FALSE	213591	FALSE	0.285	-0.111		21	0.013170902	0.035720826
FALSE	6	rs1801274	PCP4L1	ENSG00000248485	FALSE	222503	TRUE	0.992	0.616		21	0.010119798	0.026864222
FALSE	6	rs1801274	DUSP12	ENSG00000081721	FALSE	241805	FALSE	0.673	0.098		21	0.008663609	0.038160133
FALSE	6	rs1801274	ATF6	ENSG00000118217	FALSE	258345	FALSE	0.657	0.088		21	0.008197891	0.018311168
FALSE	6	rs1801274	NR1I3	ENSG00000143257	FALSE	269709	FALSE	0.531	0.019		21	0.009875359	0.036009104
FALSE	6	rs1801274	TOMM40L	ENSG00000158882	FALSE	277207	FALSE	0.558	0.033		21	0.008292477	0.007541062
FALSE	6	rs1801274	APOA2	ENSG00000158874	FALSE	284322	FALSE	0.156	-0.189		21	0.007939438	0.005456048
FALSE	6	rs1801274	AL590714.1	ENSG00000268387	FALSE	285447	FALSE	NA	NA		21		
FALSE	6	rs1801274	FCER1G	ENSG00000158869	FALSE	287254	FALSE	0.316	-0.094		21	0.007939438	0.039028986
FALSE	6	rs1801274	NDUFS2	ENSG00000158864	FALSE	293230	FALSE	0.436	-0.032		21	0.007827807	0.004722574
FALSE	7	rs10913559	VAMP4	ENSG00000117533	TRUE	0	FALSE	0.990	0.572		6	0.465952396	0.034009603
FALSE	7	rs10913559	METTL13	ENSG00000010165	FALSE	62437	FALSE	0.335	-0.085		6	0.07477361	0.117880838
FALSE	7	rs10913559	MYOC	ENSG00000034971	FALSE	66535	FALSE	0.080	-0.268		6	0.059516922	0.014071067
FALSE	7	rs10913559	MYOCOS	ENSG00000283683	FALSE	80424	FALSE	NA	NA		6	0.031820741	
FALSE	7	rs10913559	DNM3	ENSG00000197959	FALSE	98664	TRUE	0.998	0.854		6	0.022715047	0.024508996
FALSE	7	rs10913559	PRRC2C	ENSG00000117523	FALSE	125713	FALSE	0.988	0.543		6	0.014583742	0.062659857
FALSE	8	rs1772140	NUCKS1	ENSG00000069275	TRUE	0	FALSE	0.952	0.363		11	0.548724537	0.057825491
FALSE	8	rs1772140	SLC45A3	ENSG00000158715	FALSE	36707	TRUE	0.996	0.703		11	0.032611563	0.061203179
FALSE	8	rs1772140	RAB29	ENSG00000117280	FALSE	50770	FALSE	0.823	0.197		11	0.351124514	
FALSE	8	rs1772140	SLC41A1	ENSG00000133065	FALSE	71878	FALSE	0.965	0.402		11	0.040334062	0.023769683
FALSE	8	rs1772140	ELK4	ENSG00000158711	FALSE	85205	FALSE	0.381	-0.061		11	0.021943107	0.045261963
FALSE	8	rs1772140	PM20D1	ENSG00000162877	FALSE	110809	FALSE	0.706	0.116		11	0.024174948	0.012204163
FALSE	8	rs1772140	MFSD4A	ENSG00000174514	FALSE	114298	FALSE	0.689	0.106		11	0.017915939	
FALSE	8	rs1772140	CDK18	ENSG00000117266	FALSE	184423	FALSE	0.839	0.210		11	0.013082722	0.009799677
FALSE	8	rs1772140	SLC26A9	ENSG00000174502	FALSE	195832	FALSE	0.541	0.025		11	0.01596957	0.013701926
FALSE	8	rs1772140	BLACAT1	ENSG00000281406	FALSE	260125	FALSE	NA	NA		11	0.009043993	
FALSE	8	rs1772140	LEMD1	ENSG00000186007	FALSE	261262	FALSE	0.543	0.025		11	0.009043993	0.030087109
FALSE	9	rs11557080	NUCKS1	ENSG00000069275	TRUE	6265	FALSE	0.952	0.363		11	0.548724537	0.057825491
FALSE	9	rs11557080	RAB29	ENSG00000117280	FALSE	11539	FALSE	0.823	0.197		11	0.351124514	
FALSE	9	rs11557080	SLC41A1	ENSG00000133065	FALSE	32647	FALSE	0.965	0.402		11	0.040334062	0.023769683
FALSE	9	rs11557080	PM20D1	ENSG00000162877	FALSE	71578	FALSE	0.706	0.116		11	0.024174948	0.012204163

FALSE	9	rs11557080	SLC45A3	ENSG00000158715	FALSE	75938	TRUE	0.996	0.703		11	0.032611563	0.061203179
FALSE	9	rs11557080	ELK4	ENSG00000158711	FALSE	124436	FALSE	0.381	-0.061		11	0.021943107	0.045261963
FALSE	9	rs11557080	MFSD4A	ENSG00000174514	FALSE	153529	FALSE	0.689	0.106		11	0.017915939	
FALSE	9	rs11557080	SLC26A9	ENSG00000174502	FALSE	156601	FALSE	0.541	0.025		11	0.01596957	0.013701926
FALSE	9	rs11557080	CDK18	ENSG00000117266	FALSE	223654	FALSE	0.839	0.210		11	0.013082722	0.009799677
FALSE	9	rs11557080	BLACAT1	ENSG00000281406	FALSE	299356	FALSE	NA	NA		11	0.009043993	
FALSE	9	rs11557080	LEMD1	ENSG00000186007	FALSE	300493	FALSE	0.543	0.025		11	0.009043993	0.030087109
FALSE	10	rs708723	RAB29	ENSG00000117280	TRUE	3552	FALSE	0.823	0.197		11	0.351124514	
FALSE	10	rs708723	NUCKS1	ENSG00000069275	FALSE	14252	FALSE	0.952	0.363		11	0.548724537	0.057825491
FALSE	10	rs708723	SLC41A1	ENSG00000133065	FALSE	24660	FALSE	0.965	0.402		11	0.040334062	0.023769683
FALSE	10	rs708723	PM20D1	ENSG00000162877	FALSE	63591	FALSE	0.706	0.116		11	0.024174948	0.012204163
FALSE	10	rs708723	SLC45A3	ENSG00000158715	FALSE	83925	TRUE	0.996	0.703		11	0.032611563	0.061203179
FALSE	10	rs708723	ELK4	ENSG00000158711	FALSE	132423	FALSE	0.381	-0.061		11	0.021943107	0.045261963
FALSE	10	rs708723	SLC26A9	ENSG00000174502	FALSE	148614	FALSE	0.541	0.025		11	0.01596957	0.013701926
FALSE	10	rs708723	MFSD4A	ENSG00000174514	FALSE	161516	FALSE	0.689	0.106		11	0.017915939	
FALSE	10	rs708723	CDK18	ENSG00000117266	FALSE	231641	FALSE	0.839	0.210		11	0.013082722	0.009799677
FALSE	10	rs708723	BLACAT1	ENSG00000281406	FALSE	307343	FALSE	NA	NA		11	0.009043993	
FALSE	10	rs708723	LEMD1	ENSG00000186007	FALSE	308480	FALSE	0.543	0.025		11	0.009043993	0.030087109
TRUE	11	rs16846351	ITPKB	ENSG00000143772	TRUE	0	TRUE	0.999	0.923		7	0.735941768	0.5402806
FALSE	11	rs16846351	STUM	ENSG00000203685	FALSE	69817	FALSE	0.797	0.178		7	0.02694037	
FALSE	11	rs16846351	PSEN2	ENSG00000143801	FALSE	191137	FALSE	0.996	0.715		7	0.024573326	0.007812558
FALSE	11	rs16846351	ENSG00000288674	ENSG00000288674	FALSE	191137	FALSE	NA	NA		7		
FALSE	11	rs16846351	COQ8A	ENSG00000163050	FALSE	261239	FALSE	0.534	0.021		7	0.017844116	
FALSE	11	rs16846351	PARP1	ENSG00000143799	FALSE	270893	FALSE	0.985	0.514		7	0.013686241	0.013440237
FALSE	11	rs16846351	CDC42BPA	ENSG00000143776	FALSE	310818	FALSE	0.949	0.357		7	0.013778378	0.013444512
TRUE	12	rs4653767	ITPKB	ENSG00000143772	TRUE	0	TRUE	0.999	0.923		6	0.735941768	0.5402806
FALSE	12	rs4653767	STUM	ENSG00000203685	FALSE	122681	FALSE	0.797	0.178		6	0.02694037	
FALSE	12	rs4653767	PSEN2	ENSG00000143801	FALSE	138273	FALSE	0.996	0.715		6	0.024573326	0.007812558
FALSE	12	rs4653767	ENSG00000288674	ENSG00000288674	FALSE	138273	FALSE	NA	NA		6		
FALSE	12	rs4653767	COQ8A	ENSG00000163050	FALSE	208375	FALSE	0.534	0.021		6	0.017844116	
FALSE	12	rs4653767	CDC42BPA	ENSG00000143776	FALSE	257954	FALSE	0.949	0.357		6	0.013778378	0.013444512
TRUE	13	rs1326293	SIPA1L2	ENSG00000116991	TRUE	0	TRUE	0.931	0.317		2	0.795553267	0.8489601
FALSE	13	rs1326293	MAP10	ENSG00000212916	FALSE	273919	FALSE	0.270	-0.120		2	0.020959366	0.02569071
FALSE	14	rs76116224	KCNS3	ENSG00000170745	TRUE	0	FALSE	0.146	-0.197		4	0.862785161	0.805789253
FALSE	14	rs76116224	MSGN1	ENSG00000151379	FALSE	148783	FALSE	0.287	-0.110		4	0.039600812	0.025004794
FALSE	14	rs76116224	SMC6	ENSG00000163029	FALSE	166339	TRUE	0.438	-0.031		4	0.023899406	0.099528815

FALSE	14	rs76116224	GEN1	ENSG00000178295	FALSE	177635	FALSE	0.363	-0.071		4	0.018891973	0.025043167
TRUE	15	rs12613887	WDR43	ENSG00000163811	TRUE	0	TRUE	0.875	0.242		9		
FALSE	15	rs12613887	TOGARAM2	ENSG00000189350	FALSE	40707	FALSE	0.572	0.041		9		
FALSE	15	rs12613887	TRMT61B	ENSG00000171103	FALSE	45595	FALSE	0.408	-0.046		9		
FALSE	15	rs12613887	SPDYA	ENSG00000163806	FALSE	65293	FALSE	0.674	0.098		9		
FALSE	15	rs12613887	PPP1CB	ENSG00000213639	FALSE	112964	FALSE	0.727	0.130		9		
FALSE	15	rs12613887	PCARE	ENSG00000179270	FALSE	145072	FALSE	0.148	-0.196		9		
FALSE	15	rs12613887	CLIP4	ENSG00000115295	FALSE	181801	FALSE	0.466	-0.015		9		
FALSE	15	rs12613887	PLB1	ENSG00000163803	FALSE	271762	FALSE	0.486	-0.004		9		
FALSE	15	rs12613887	ALK	ENSG00000171094	FALSE	276870	FALSE	0.634	0.075		9		
TRUE	16	rs398290	BIRC6	ENSG00000115760	TRUE	0	TRUE	0.961	0.388	0.027850426	8		
FALSE	16	rs398290	YIPF4	ENSG00000119820	FALSE	69053	FALSE	0.760	0.152		8		
FALSE	16	rs398290	NLRC4	ENSG00000091106	FALSE	119915	FALSE	0.598	0.055		8		
FALSE	16	rs398290	SLC30A6	ENSG00000152683	FALSE	161268	FALSE	0.179	-0.172		8		
FALSE	16	rs398290	SPAST	ENSG00000021574	FALSE	228010	FALSE	0.889	0.259		8		
FALSE	16	rs398290	TTC27	ENSG00000018699	FALSE	242383	FALSE	0.660	0.090		8		
FALSE	16	rs398290	DPY30	ENSG00000162961	FALSE	345842	FALSE	0.403	-0.049		8		
FALSE	16	rs398290	MEMO1	ENSG00000162959	FALSE	374417	FALSE	0.763	0.155		8		
TRUE	17	rs9309337	XPO1	ENSG00000082898	TRUE	0	TRUE	0.997	0.805		9		
FALSE	17	rs9309337	USP34	ENSG00000115464	FALSE	62826	FALSE	0.963	0.397		9		
FALSE	17	rs9309337	FAM161A	ENSG00000170264	FALSE	290935	FALSE	0.321	-0.092		9		
FALSE	17	rs9309337	CCT4	ENSG00000115484	FALSE	334172	FALSE	0.549	0.029		9		
FALSE	17	rs9309337	COMMD1	ENSG00000173163	FALSE	354811	FALSE	0.507	0.007		9		
FALSE	17	rs9309337	C2orf74	ENSG00000237651	FALSE	369085	FALSE	0.547	0.028		9		
FALSE	17	rs9309337	SANBR	ENSG00000162929	FALSE	395879	FALSE	0.538	0.023		9		
FALSE	17	rs9309337	PEX13	ENSG00000162928	FALSE	481923	FALSE	0.408	-0.046		9		
FALSE	17	rs9309337	PUS10	ENSG00000162927	FALSE	515654	FALSE	0.761	0.153		9		
FALSE	18	rs4581940	NFU1	ENSG00000169599	TRUE	0	FALSE	0.703	0.114	0.012590168	6		
FALSE	18	rs4581940	GFPT1	ENSG00000198380	FALSE	18198	FALSE	0.857	0.227		6		
FALSE	18	rs4581940	AAK1	ENSG00000115977	FALSE	52549	TRUE	0.968	0.410		6		
FALSE	18	rs4581940	ANTXR1	ENSG00000169604	FALSE	156121	FALSE	0.271	-0.119		6		
FALSE	18	rs4581940	ANXA4	ENSG00000196975	FALSE	238977	FALSE	0.232	-0.141		6		
FALSE	18	rs4581940	GMCL1	ENSG00000087338	FALSE	424212	FALSE	0.497	0.001		6		
FALSE	19	rs72819488	PROM2	ENSG00000155066	TRUE	0	FALSE	0.748	0.144	0.076072142	12		0.070914514
FALSE	19	rs72819488	KCNIP3	ENSG00000115041	FALSE	13028	FALSE	0.766	0.157		12		0.733451682
FALSE	19	rs72819488	ZNF892	ENSG00000233757	FALSE	24525	FALSE	NA	NA		12		

FALSE	19	rs72819488	ZNF2	ENSG00000275111	FALSE	99982	FALSE	NA	NA	12		0.016158741
FALSE	19	rs72819488	FAHD2A	ENSG00000115042	FALSE	118409	FALSE	0.449	-0.026	12		0.052096176
FALSE	19	rs72819488	ZNF514	ENSG00000144026	FALSE	118634	FALSE	0.267	-0.121	12		0.0093047
FALSE	19	rs72819488	ENSG00000289685	ENSG00000289685	FALSE	118911	FALSE	NA	NA	12		
FALSE	19	rs72819488	MRPS5	ENSG00000144029	FALSE	162296	FALSE	0.901	0.274	12		0.010335558
FALSE	19	rs72819488	TRIM43B	ENSG00000144010	FALSE	192709	FALSE	NA	NA	12		
FALSE	19	rs72819488	MAL	ENSG00000172005	FALSE	230310	TRUE	0.996	0.711	12		0.008720193
FALSE	19	rs72819488	TRIM43	ENSG00000144015	FALSE	307702	FALSE	0.066	-0.291	12		0.007490584
FALSE	19	rs72819488	TEKT4	ENSG00000163060	FALSE	407479	FALSE	0.255	-0.128	12		0.007309218
TRUE	20	rs11683001	MAP4K4	ENSG00000071054	TRUE	0	TRUE	0.995	0.702	7	0.844995618	0.804053866
FALSE	20	rs11683001	FLJ20373	ENSG00000233404	FALSE	88499	FALSE	0.419	-0.041	7		
FALSE	20	rs11683001	IL1R2	ENSG00000115590	FALSE	187966	FALSE	0.607	0.060	7	0.037692439	0.025178261
FALSE	20	rs11683001	IL1R1	ENSG00000115594	FALSE	260548	FALSE	0.460	-0.019	7	0.020748705	0.037438119
FALSE	20	rs11683001	RFX8	ENSG00000196460	FALSE	328882	FALSE	0.057	-0.307	7	0.017434878	0.023663767
FALSE	20	rs11683001	IL1RL2	ENSG00000115598	FALSE	383010	FALSE	0.066	-0.291	7	0.015413071	0.007664413
FALSE	20	rs11683001	CREG2	ENSG00000175874	FALSE	416491	FALSE	0.296	-0.106	7	0.015413071	0.007194509
TRUE	21	rs57891859	TMEM163	ENSG00000152128	TRUE	0	TRUE	0.998	0.882	5	0.867876172	0.924661811
FALSE	21	rs57891859	ACMSD	ENSG00000153086	FALSE	131570	FALSE	0.366	-0.069	5	0.025962669	0.007444577
FALSE	21	rs57891859	CCNT2	ENSG00000082258	FALSE	211189	FALSE	0.988	0.541	5	0.01971324	0.012412093
FALSE	21	rs57891859	MGAT5	ENSG00000152127	FALSE	252424	FALSE	0.504	0.005	5	0.015129407	0.012694869
FALSE	21	rs57891859	MAP3K19	ENSG00000176601	FALSE	257439	FALSE	0.150	-0.194	5	0.014611791	0.007329885
TRUE	22	rs17698151	TMEM163	ENSG00000152128	TRUE	30121	TRUE	0.998	0.882	6	0.867876172	0.924661811
FALSE	22	rs17698151	ACMSD	ENSG00000153086	FALSE	89495	FALSE	0.366	-0.069	6	0.025962669	0.007444577
FALSE	22	rs17698151	CCNT2	ENSG00000082258	FALSE	169114	FALSE	0.988	0.541	6	0.01971324	0.012412093
FALSE	22	rs17698151	MAP3K19	ENSG00000176601	FALSE	215364	FALSE	0.150	-0.194	6	0.014611791	0.007329885
FALSE	22	rs17698151	MGAT5	ENSG00000152127	FALSE	294499	FALSE	0.504	0.005	6	0.015129407	0.012694869
FALSE	22	rs17698151	RAB3GAP1	ENSG00000115839	FALSE	303168	FALSE	0.454	-0.023	6	0.014514133	0.012690159
TRUE	23	rs4547489	RBMS1	ENSG00000153250	TRUE	0	TRUE	0.988	0.545	3		
FALSE	23	rs4547489	ITGB6	ENSG00000115221	FALSE	116312	FALSE	0.122	-0.217	3		
FALSE	23	rs4547489	PLA2R1	ENSG00000153246	FALSE	254010	FALSE	0.797	0.178	3		
FALSE	24	rs353128	SCN2A	ENSG00000136531	TRUE	0	FALSE	0.993	0.640	4		
FALSE	24	rs353128	SCN3A	ENSG00000153253	FALSE	83936	TRUE	0.998	0.889	4		
FALSE	24	rs353128	CSRNP3	ENSG00000178662	FALSE	181661	FALSE	0.475	-0.011	4		
FALSE	24	rs353128	SLC38A11	ENSG00000169507	FALSE	332461	FALSE	0.458	-0.020	4		
TRUE	25	rs1474055	STK39	ENSG00000198648	TRUE	6800	TRUE	0.988	0.549	2	0.805206954	0.891061902
FALSE	25	rs1474055	CERS6	ENSG00000172292	FALSE	201854	FALSE	0.794	0.177	2	0.033460323	0.020073005

TRUE	26	rs73038319	TBC1D5	ENSG00000131374	TRUE	0	TRUE	0.668	0.094	2		0.73659813
FALSE	26	rs73038319	SATB1	ENSG00000182568	FALSE	66467	FALSE	0.510	0.008	2		0.15966341
FALSE	27	rs6808178	RBMS3	ENSG00000144642	TRUE	0	FALSE	0.062	-0.297	3	0.728015602	0.22128376
FALSE	27	rs6808178	ENSG00000283563	ENSG00000283563	TRUE	0	TRUE	NA	NA	3		
FALSE	27	rs6808178	ZCWPW2	ENSG00000206559	FALSE	124056	FALSE	0.433	-0.034	3	0.026388697	0.09187855
FALSE	28	rs12497850	PRKAR2A	ENSG00000114302	TRUE	0	FALSE	0.912	0.289	44	0.044696713	0.032746612
FALSE	28	rs12497850	IP6K2	ENSG00000068745	FALSE	57159	FALSE	0.890	0.261	44	0.212184046	0.332881114
FALSE	28	rs12497850	SLC25A20	ENSG00000178537	FALSE	59414	FALSE	0.619	0.067	44	0.057031891	0.004503065
FALSE	28	rs12497850	NCKIPSD	ENSG00000213672	FALSE	111148	TRUE	0.951	0.360	44	0.077344456	0.214450911
FALSE	28	rs12497850	ARIH2	ENSG00000177479	FALSE	121309	FALSE	0.873	0.240	44	0.050367047	0.00811477
FALSE	28	rs12497850	CELSR3	ENSG00000008300	FALSE	134626	FALSE	0.936	0.327	44	0.012496587	0.13163236
FALSE	28	rs12497850	SLC26A6	ENSG00000225697	FALSE	162019	FALSE	0.788	0.172	44	0.008934291	0.009443827
FALSE	28	rs12497850	TMEM89	ENSG00000183396	FALSE	175743	FALSE	0.314	-0.095	44	0.012516053	0.007010093
FALSE	28	rs12497850	UQCRC1	ENSG00000010256	FALSE	186536	FALSE	0.257	-0.127	44	0.00870877	0.00499033
FALSE	28	rs12497850	P4HTM	ENSG00000178467	FALSE	192377	FALSE	0.880	0.248	44	0.055974673	0.008424477
FALSE	28	rs12497850	COL7A1	ENSG00000114270	FALSE	202183	FALSE	0.191	-0.166	44	0.008317394	0.004503065
FALSE	28	rs12497850	WDR6	ENSG00000178252	FALSE	209550	FALSE	0.911	0.288	44	0.106208964	0.00466498
FALSE	28	rs12497850	DALRD3	ENSG00000178149	FALSE	217976	FALSE	0.518	0.012	44	0.018088356	0.006028297
FALSE	28	rs12497850	NDUFAF3	ENSG00000178057	FALSE	222947	FALSE	0.800	0.181	44	0.015427035	0.005796478
FALSE	28	rs12497850	IMPDH2	ENSG00000178035	FALSE	226813	FALSE	0.820	0.195	44	0.017982782	0.004464239
FALSE	28	rs12497850	ENSG00000290315	ENSG00000290315	FALSE	226833	FALSE	NA	NA	44		
FALSE	28	rs12497850	QRICH1	ENSG00000198218	FALSE	232195	FALSE	0.784	0.169	44	0.034780066	0.009111993
FALSE	28	rs12497850	UCN2	ENSG00000145040	FALSE	233731	FALSE	0.209	-0.154	44	0.011003901	0.004669136
FALSE	28	rs12497850	PFKFB4	ENSG00000114268	FALSE	235497	FALSE	0.759	0.152	44	0.011604011	0.009273557
FALSE	28	rs12497850	SHISA5	ENSG00000164054	FALSE	292686	FALSE	0.910	0.287	44	0.008198231	0.004503065
FALSE	28	rs12497850	QARS1	ENSG00000172053	FALSE	298420	FALSE	0.497	0.001	44	0.032492016	
FALSE	28	rs12497850	USP19	ENSG00000172046	FALSE	310534	FALSE	0.792	0.175	44	0.052754535	0.007746792
FALSE	28	rs12497850	LAMB2	ENSG00000172037	FALSE	323602	FALSE	0.937	0.330	44	0.011116409	0.005734465
FALSE	28	rs12497850	TREX1	ENSG00000213689	FALSE	325901	FALSE	0.849	0.219	44	0.008440096	0.006964341
FALSE	28	rs12497850	ATRIP	ENSG00000164053	FALSE	325901	FALSE	0.696	0.110	44	0.007317619	0.007706165
FALSE	28	rs12497850	TMA7	ENSG00000232112	FALSE	349329	FALSE	0.476	-0.010	44	0.007803297	0.004607677
FALSE	28	rs12497850	CCDC51	ENSG00000164051	FALSE	353079	FALSE	0.597	0.054	44	0.008300078	0.004503065
FALSE	28	rs12497850	PLXNB1	ENSG00000164050	FALSE	363450	FALSE	0.679	0.100	44	0.00800138	0.004464239
FALSE	28	rs12497850	CCDC71	ENSG00000177352	FALSE	365023	FALSE	0.278	-0.115	44	0.012743739	0.006664143
FALSE	28	rs12497850	KLHDC8B	ENSG00000185909	FALSE	374086	FALSE	0.691	0.107	44	0.010630991	0.008966452
FALSE	28	rs12497850	C3orf84	ENSG00000236980	FALSE	380122	FALSE	0.159	-0.186	44	0.009562395	0.00441637

FALSE	28	rs12497850	FBXW12	ENSG00000164049	FALSE	392279	FALSE	0.358	-0.073		44	0.008343159	0.004464239
FALSE	28	rs12497850	IHO1	ENSG00000173421	FALSE	400916	FALSE	0.220	-0.148		44	0.011087105	
FALSE	28	rs12497850	SPINK8	ENSG00000229453	FALSE	459794	FALSE	0.426	-0.037		44	0.009303315	0.004464239
FALSE	28	rs12497850	C3orf62	ENSG00000188315	FALSE	471084	FALSE	0.560	0.034		44		0.006311645
FALSE	28	rs12497850	USP4	ENSG00000114316	FALSE	479632	FALSE	0.932	0.319		44		0.007895463
FALSE	28	rs12497850	NME6	ENSG00000172113	FALSE	491770	FALSE	0.560	0.034		44	0.008828495	0.006715994
FALSE	28	rs12497850	ZNF589	ENSG00000164048	FALSE	494202	FALSE	0.224	-0.146		44	0.010380696	0.005280307
FALSE	28	rs12497850	GPX1	ENSG00000233276	FALSE	559662	FALSE	0.893	0.264		44		0.007038717
FALSE	28	rs12497850	ENSG00000290318	ENSG00000290318	FALSE	559664	FALSE	NA	NA		44		
FALSE	28	rs12497850	RHOA	ENSG00000067560	FALSE	561627	FALSE	0.059	-0.303		44		0.004787382
FALSE	28	rs12497850	CAMP	ENSG00000164047	FALSE	567964	FALSE	0.086	-0.259		44	0.011029409	0.00441637
FALSE	28	rs12497850	CDC25A	ENSG00000164045	FALSE	605038	FALSE	0.518	0.012		44		0.00441637
FALSE	28	rs12497850	MAP4	ENSG00000047849	FALSE	704183	FALSE	0.810	0.188		44		0.007347022
FALSE	29	rs55961674	KPNA1	ENSG00000114030	TRUE	0	FALSE	0.823	0.198		13	0.271544953	0.73198158
FALSE	29	rs55961674	PARP9	ENSG00000138496	FALSE	34643	FALSE	0.493	-0.001	0.011585734	13	0.194140111	0.069798287
FALSE	29	rs55961674	DTX3L	ENSG00000163840	FALSE	71057	FALSE	0.717	0.124		13	0.095148525	0.019283106
FALSE	29	rs55961674	WDR5B	ENSG00000196981	FALSE	77219	FALSE	0.598	0.054		13	0.157646523	0.019380216
FALSE	29	rs55961674	FAM162A	ENSG00000114023	FALSE	80947	FALSE	0.738	0.138		13	0.125910521	0.012258235
FALSE	29	rs55961674	PARP15	ENSG00000173200	FALSE	84347	FALSE	0.115	-0.225		13	0.02601128	0.014241568
FALSE	29	rs55961674	MIX23	ENSG00000160124	FALSE	110050	FALSE	0.845	0.216		13	0.028504431	
FALSE	29	rs55961674	CSTA	ENSG00000121552	FALSE	151312	FALSE	0.865	0.234		13	0.021222073	0.006053641
FALSE	29	rs55961674	PARP14	ENSG00000173193	FALSE	187558	FALSE	0.581	0.046		13	0.017057198	0.005974961
FALSE	29	rs55961674	CASR	ENSG00000036828	FALSE	201652	FALSE	0.053	-0.317		13	0.013132538	0.00588291
FALSE	29	rs55961674	HSPBAP1	ENSG00000169087	FALSE	246718	FALSE	0.838	0.210		13	0.011366385	0.006564933
FALSE	29	rs55961674	SLC49A4	ENSG00000138463	FALSE	301788	FALSE	0.776	0.164		13	0.010948215	
FALSE	29	rs55961674	CD86	ENSG00000114013	FALSE	372142	TRUE	0.966	0.403		13	0.00884679	0.00588291
FALSE	30	rs6803771	MED12L	ENSG00000144893	TRUE	0	FALSE	0.986	0.523		7	0.389052331	0.14545432
FALSE	30	rs6803771	P2RY12	ENSG00000169313	FALSE	8592	TRUE	0.996	0.727		7	0.378167033	0.33879775
FALSE	30	rs6803771	IGSF10	ENSG00000152580	FALSE	32039	FALSE	0.414	-0.043		7	0.031020103	0.03727355
FALSE	30	rs6803771	P2RY13	ENSG00000181631	FALSE	63796	FALSE	0.887	0.256		7	0.029263558	0.025387341
FALSE	30	rs6803771	GPR87	ENSG00000138271	FALSE	76525	FALSE	0.918	0.298		7	0.028483393	0.007866387
FALSE	30	rs6803771	P2RY14	ENSG00000174944	FALSE	114803	FALSE	0.965	0.400		7	0.023821607	0.010589266
FALSE	30	rs6803771	GPR171	ENSG00000174946	FALSE	190130	FALSE	0.026	-0.415		7	0.017586116	0.007812558
FALSE	31	rs16864251	MBNL1	ENSG00000152601	TRUE	0	FALSE	0.991	0.587		12		0.012746658
FALSE	31	rs16864251	SUCNR1	ENSG00000198829	FALSE	491561	FALSE	0.051	-0.321		12	0.026987795	0.020211661
FALSE	31	rs16864251	AADAC	ENSG00000114771	FALSE	547692	FALSE	0.301	-0.103		12	0.010780717	0.007662146

FALSE	31	rs16864251	AADACL2	ENSG00000197953	FALSE	614841	FALSE	0.290	-0.109	12	0.013066905	0.007745196
FALSE	31	rs16864251	IGSF10	ENSG00000152580	FALSE	915119	FALSE	0.414	-0.043	12	0.031020103	0.03727355
FALSE	31	rs16864251	MED12L	ENSG00000144893	FALSE	939108	FALSE	0.986	0.523	12	0.389052331	0.14545432
FALSE	31	rs16864251	P2RY12	ENSG00000169313	FALSE	991427	TRUE	0.996	0.727	12	0.378167033	0.33879775
FALSE	31	rs16864251	P2RY13	ENSG00000181631	FALSE	1046631	FALSE	0.887	0.256	12	0.029263558	0.025387341
FALSE	31	rs16864251	GPR87	ENSG00000138271	FALSE	1059360	FALSE	0.918	0.298	12	0.028483393	0.007866387
FALSE	31	rs16864251	P2RY14	ENSG00000174944	FALSE	1097638	FALSE	0.965	0.400	12	0.023821607	0.010589266
FALSE	31	rs16864251	GPR171	ENSG00000174946	FALSE	1172965	FALSE	0.026	-0.415	12	0.017586116	0.007812558
FALSE	31	rs16864251	CLRN1	ENSG00000163646	FALSE	1403454	FALSE	0.027	-0.405	12	0.010942318	0.007662146
FALSE	32	rs1450522	SPTSSB	ENSG00000196542	TRUE	0	FALSE	0.930	0.317	5	0.709308494	0.763676
FALSE	32	rs1450522	NMD3	ENSG00000169251	FALSE	105738	FALSE	0.907	0.283	5	0.19302224	0.02349229
FALSE	32	rs1450522	OTOL1	ENSG00000182447	FALSE	137538	FALSE	0.099	-0.243	5	0.038329825	0.007812558
FALSE	32	rs1450522	B3GALNT1	ENSG00000169255	FALSE	253859	FALSE	0.687	0.105	5	0.035382337	0.007965676
FALSE	32	rs1450522	PPM1L	ENSG00000163590	FALSE	280368	TRUE	0.989	0.555	5	0.023957104	0.013444512
TRUE	33	rs6806917	PIK3CA	ENSG00000121879	TRUE	0	TRUE	0.999	0.931	8		
FALSE	33	rs6806917	KCNMB3	ENSG00000171121	FALSE	77541	FALSE	0.483	-0.006	8		
FALSE	33	rs6806917	ZMAT3	ENSG00000172667	FALSE	86935	FALSE	0.948	0.352	8		
FALSE	33	rs6806917	ZNF639	ENSG00000121864	FALSE	163881	FALSE	0.649	0.084	8		
FALSE	33	rs6806917	MFN1	ENSG00000171109	FALSE	188559	FALSE	0.568	0.038	8		
FALSE	33	rs6806917	GNB4	ENSG00000114450	FALSE	236938	FALSE	0.121	-0.219	8		
FALSE	33	rs6806917	KCNMB2	ENSG00000197584	FALSE	314721	FALSE	0.020	-0.446	8		
FALSE	33	rs6806917	ENSG00000275163	ENSG00000275163	FALSE	315850	FALSE	NA	NA	8		
FALSE	34	rs10513789	MCCC1	ENSG00000078070	TRUE	0	FALSE	0.966	0.404	6	0.298905581	0.500904688
FALSE	34	rs10513789	DCUN1D1	ENSG00000043093	FALSE	52982	FALSE	0.788	0.172	6	0.264706701	0.414915365
FALSE	34	rs10513789	LAMP3	ENSG00000078081	FALSE	83280	TRUE	0.970	0.424	6	0.027570635	0.016990971
FALSE	34	rs10513789	ATP11B	ENSG00000058063	FALSE	117306	FALSE	0.746	0.143	6	0.018459678	0.007917363
FALSE	34	rs10513789	MCF2L2	ENSG00000053524	FALSE	139106	FALSE	0.473	-0.011	6	0.016226299	0.008968432
FALSE	34	rs10513789	B3GNT5	ENSG00000176597	FALSE	214318	FALSE	0.022	-0.437	6	0.016064126	0.005158667
FALSE	35	rs873786	GAK	ENSG00000178950	TRUE	0	TRUE	0.989	0.563	16	0.219038133	0.164934066
FALSE	35	rs873786	TMEM175	ENSG00000127419	FALSE	6820	FALSE	0.966	0.406	16	0.559257672	0.494822208
FALSE	35	rs873786	DGKQ	ENSG00000145214	FALSE	33320	FALSE	0.944	0.345	16	0.112689494	0.1144256
FALSE	35	rs873786	SLC26A1	ENSG00000145217	FALSE	53506	FALSE	0.613	0.063	16	0.059530262	0.02499493
FALSE	35	rs873786	IDUA	ENSG00000127415	FALSE	61430	FALSE	0.987	0.535	16	0.082867251	0.030537374
FALSE	35	rs873786	FGFRL1	ENSG00000127418	FALSE	84369	FALSE	0.867	0.236	16	0.025095244	0.012206176
FALSE	35	rs873786	CPLX1	ENSG00000168993	FALSE	99438	FALSE	0.718	0.124	16	0.019378217	0.004408569
FALSE	35	rs873786	RNF212	ENSG00000178222	FALSE	130683	FALSE	0.428	-0.036	16	0.011882414	0.004294868

FALSE	35	rs873786	PCGF3	ENSG00000185619	FALSE	155478	FALSE	0.956	0.374		16	0.056788501	0.007727499
FALSE	35	rs873786	SLC49A3	ENSG00000169026	FALSE	236295	FALSE	0.540	0.024		16	0.012115942	
FALSE	35	rs873786	SPON2	ENSG00000159674	FALSE	241365	FALSE	0.178	-0.173		16	0.007950527	0.007928382
FALSE	35	rs873786	MYL5	ENSG00000215375	FALSE	243538	FALSE	0.497	0.001		16	0.006087064	0.007388624
FALSE	35	rs873786	ATP5ME	ENSG00000169020	FALSE	251236	FALSE	0.805	0.185		16	0.00453456	
FALSE	35	rs873786	PDE6B	ENSG00000133256	FALSE	254784	FALSE	0.579	0.045		16	0.005342248	0.004257837
FALSE	35	rs873786	CTBP1	ENSG00000159692	FALSE	285878	FALSE	0.722	0.126		16	0.006023336	0.006206608
FALSE	35	rs873786	TMEM271	ENSG00000273238	FALSE	349271	FALSE	NA	NA		16	0.003605475	
TRUE	36	rs34311866	TMEM175	ENSG00000127419	TRUE	0	FALSE	0.966	0.406	1	15	0.559257672	0.494822208
FALSE	36	rs34311866	DGKQ	ENSG00000145214	FALSE	728	FALSE	0.944	0.345		15	0.112689494	0.1144256
FALSE	36	rs34311866	SLC26A1	ENSG00000145217	FALSE	20914	FALSE	0.613	0.063		15	0.059530262	0.02499493
FALSE	36	rs34311866	GAK	ENSG00000178950	FALSE	25786	TRUE	0.989	0.563		15	0.219038133	0.164934066
FALSE	36	rs34311866	IDUA	ENSG00000127415	FALSE	28838	FALSE	0.987	0.535		15	0.082867251	0.030537374
FALSE	36	rs34311866	FGFRL1	ENSG00000127418	FALSE	51777	FALSE	0.867	0.236		15	0.025095244	0.012206176
FALSE	36	rs34311866	RNF212	ENSG00000178222	FALSE	98091	FALSE	0.428	-0.036		15	0.011882414	0.004294868
FALSE	36	rs34311866	CPLX1	ENSG00000168993	FALSE	132030	FALSE	0.718	0.124		15	0.019378217	0.004408569
FALSE	36	rs34311866	PCGF3	ENSG00000185619	FALSE	188070	FALSE	0.956	0.374		15	0.056788501	0.007727499
FALSE	36	rs34311866	SPON2	ENSG00000159674	FALSE	208773	FALSE	0.178	-0.173		15	0.007950527	0.007928382
FALSE	36	rs34311866	CTBP1	ENSG00000159692	FALSE	253286	FALSE	0.722	0.126		15	0.006023336	0.006206608
FALSE	36	rs34311866	SLC49A3	ENSG00000169026	FALSE	268887	FALSE	0.540	0.024		15	0.012115942	
FALSE	36	rs34311866	MYL5	ENSG00000215375	FALSE	276130	FALSE	0.497	0.001		15	0.006087064	0.007388624
FALSE	36	rs34311866	ATP5ME	ENSG00000169020	FALSE	283828	FALSE	0.805	0.185		15	0.00453456	
FALSE	36	rs34311866	PDE6B	ENSG00000133256	FALSE	287376	FALSE	0.579	0.045		15	0.005342248	0.004257837
FALSE	37	rs4698412	BST1	ENSG00000109743	TRUE	0	FALSE	0.937	0.329		9	0.760085801	0.242261828
FALSE	37	rs4698412	CD38	ENSG00000004468	FALSE	43109	TRUE	0.993	0.628		9	0.105459237	0.01363498
FALSE	37	rs4698412	FAM200B	ENSG00000237765	FALSE	44719	FALSE	0.705	0.115		9	0.039648074	0.277019308
FALSE	37	rs4698412	FBXL5	ENSG00000118564	FALSE	53487	FALSE	0.442	-0.029		9	0.037461691	0.421567405
FALSE	37	rs4698412	CC2D2A	ENSG00000048342	FALSE	133614	FALSE	0.365	-0.070		9	0.015732397	0.0052711
FALSE	37	rs4698412	FGFBP1	ENSG00000137440	FALSE	200411	FALSE	0.003	-0.727		9	0.011707845	0.005199374
FALSE	37	rs4698412	FGFBP2	ENSG00000137441	FALSE	225079	FALSE	0.008	-0.578		9	0.01143862	0.005163795
FALSE	37	rs4698412	PROM1	ENSG00000007062	FALSE	227910	FALSE	0.120	-0.220		9	0.008139739	0.005119271
FALSE	37	rs4698412	C1QTNF7	ENSG00000163145	FALSE	288998	FALSE	0.050	-0.325		9	0.004895032	0.005119271
TRUE	38	rs34025766	LCORL	ENSG00000178177	TRUE	0	TRUE	0.987	0.540		6	0.814537819	0.878999725
FALSE	38	rs34025766	NCAPG	ENSG00000109805	FALSE	127240	FALSE	0.544	0.026		6	0.050243772	0.023972959
FALSE	38	rs34025766	DCAF16	ENSG00000163257	FALSE	161347	FALSE	0.470	-0.013		6	0.044253706	0.032253305
FALSE	38	rs34025766	FAM184B	ENSG00000047662	FALSE	190484	FALSE	0.647	0.082		6	0.036921355	0.023972959

FALSE	38	rs34025766	MED28	ENSG00000118579	FALSE	338000	FALSE	0.630	0.073		6	0.013319052	0.01252086
FALSE	38	rs34025766	LAP3	ENSG00000002549	FALSE	364133	FALSE	0.123	-0.217		6	0.014599919	0.009396982
TRUE	39	rs3816248	SCARB2	ENSG00000138760	TRUE	0	TRUE	0.999	1.045		14	0.545993388	0.428399969
FALSE	39	rs3816248	NUP54	ENSG00000138750	FALSE	31898	FALSE	0.770	0.160		14	0.016757827	0.017499236
FALSE	39	rs3816248	FAM47E	ENSG00000189157	FALSE	33657	FALSE	0.767	0.158		14	0.424657749	0.14204084
FALSE	39	rs3816248	ART3	ENSG00000156219	FALSE	66335	FALSE	0.898	0.270		14	0.017198956	0.003501113
FALSE	39	rs3816248	FAM47E-STBD1	ENSG00000272414	FALSE	71338	FALSE	NA	NA		14	0.391151116	0.3096545
FALSE	39	rs3816248	STBD1	ENSG00000118804	FALSE	126350	FALSE	NA	NA		14	0.230084152	
FALSE	39	rs3816248	CCDC158	ENSG00000163749	FALSE	132614	FALSE	0.493	-0.001		14	0.030368633	0.022608937
FALSE	39	rs3816248	CXCL11	ENSG00000169248	FALSE	138968	FALSE	0.018	-0.463		14	0.033438791	0.003501113
FALSE	39	rs3816248	CXCL10	ENSG00000169245	FALSE	156886	FALSE	0.024	-0.429		14	0.015781999	0.006299078
FALSE	39	rs3816248	CXCL9	ENSG00000138755	FALSE	172874	FALSE	0.015	-0.492		14	0.013940548	0.006299078
FALSE	39	rs3816248	SDAD1	ENSG00000198301	FALSE	189421	FALSE	0.652	0.085		14	0.010104805	0.00348458
FALSE	39	rs3816248	NAAA	ENSG00000138744	FALSE	239370	FALSE	0.719	0.125		14	0.017563781	0.004213103
FALSE	39	rs3816248	SHROOM3	ENSG00000138771	FALSE	254846	FALSE	0.914	0.293		14	0.014105103	0.003787008
FALSE	39	rs3816248	PPEF2	ENSG00000156194	FALSE	277812	FALSE	0.383	-0.061		14	0.004458627	0.003447174
TRUE	40	rs1465922	SCARB2	ENSG00000138760	TRUE	0	TRUE	0.999	1.045		13	0.545993388	0.428399969
FALSE	40	rs1465922	FAM47E	ENSG00000189157	TRUE	0	FALSE	0.767	0.158		13	0.424657749	0.14204084
FALSE	40	rs1465922	FAM47E-STBD1	ENSG00000272414	FALSE	36173	FALSE	NA	NA		13	0.391151116	0.3096545
FALSE	40	rs1465922	NUP54	ENSG00000138750	FALSE	67063	FALSE	0.770	0.160		13	0.016757827	0.017499236
FALSE	40	rs1465922	STBD1	ENSG00000118804	FALSE	91185	FALSE	NA	NA		13	0.230084152	
FALSE	40	rs1465922	CCDC158	ENSG00000163749	FALSE	97449	FALSE	0.493	-0.001		13	0.030368633	0.022608937
FALSE	40	rs1465922	ART3	ENSG00000156219	FALSE	101500	FALSE	0.898	0.270		13	0.017198956	0.003501113
FALSE	40	rs1465922	CXCL11	ENSG00000169248	FALSE	174133	FALSE	0.018	-0.463		13	0.033438791	0.003501113
FALSE	40	rs1465922	CXCL10	ENSG00000169245	FALSE	192051	FALSE	0.024	-0.429		13	0.015781999	0.006299078
FALSE	40	rs1465922	CXCL9	ENSG00000138755	FALSE	208039	FALSE	0.015	-0.492		13	0.013940548	0.006299078
FALSE	40	rs1465922	SHROOM3	ENSG00000138771	FALSE	219681	FALSE	0.914	0.293		13	0.014105103	0.003787008
FALSE	40	rs1465922	SDAD1	ENSG00000198301	FALSE	224586	FALSE	0.652	0.085		13	0.010104805	0.00348458
FALSE	40	rs1465922	NAAA	ENSG00000138744	FALSE	274535	FALSE	0.719	0.125		13	0.017563781	0.004213103
FALSE	41	rs6854006	FAM47E	ENSG00000189157	TRUE	0	FALSE	0.767	0.158	0.024612164	12	0.424657749	0.14204084
FALSE	41	rs6854006	FAM47E-STBD1	ENSG00000272414	TRUE	0	FALSE	NA	NA		12	0.391151116	0.3096545
FALSE	41	rs6854006	STBD1	ENSG00000118804	FALSE	35706	FALSE	NA	NA		12	0.230084152	
FALSE	41	rs6854006	SCARB2	ENSG00000138760	FALSE	36491	TRUE	0.999	1.045		12	0.545993388	0.428399969
FALSE	41	rs6854006	CCDC158	ENSG00000163749	FALSE	41970	FALSE	0.493	-0.001		12	0.030368633	0.022608937
FALSE	41	rs6854006	NUP54	ENSG00000138750	FALSE	122542	FALSE	0.770	0.160		12	0.016757827	0.017499236
FALSE	41	rs6854006	ART3	ENSG00000156219	FALSE	156979	FALSE	0.898	0.270		12	0.017198956	0.003501113

FALSE	41	rs6854006	SHROOM3	ENSG00000138771	FALSE	164202	FALSE	0.914	0.293		12	0.014105103	0.003787008
FALSE	41	rs6854006	CXCL11	ENSG00000169248	FALSE	229612	FALSE	0.018	-0.463		12	0.033438791	0.003501113
FALSE	41	rs6854006	CXCL10	ENSG00000169245	FALSE	247530	FALSE	0.024	-0.429		12	0.015781999	0.006299078
FALSE	41	rs6854006	CXCL9	ENSG00000138755	FALSE	263518	FALSE	0.015	-0.492		12	0.013940548	0.006299078
FALSE	41	rs6854006	SDAD1	ENSG00000198301	FALSE	280065	FALSE	0.652	0.085		12	0.010104805	0.00348458
TRUE	42	rs356203	SNCA	ENSG00000145335	TRUE	0	TRUE	1.000	3.795		2	0.833159763	0.701174087
FALSE	42	rs356203	MMRN1	ENSG00000138722	FALSE	122452	FALSE	0.999	1.269		2	0.236753997	0.18473212
TRUE	43	rs2619356	SNCA	ENSG00000145335	TRUE	0	TRUE	1.000	3.795		3	0.833159763	0.701174087
FALSE	43	rs2619356	MMRN1	ENSG00000138722	FALSE	44369	FALSE	0.999	1.269		3	0.236753997	0.18473212
FALSE	43	rs2619356	CCSER1	ENSG00000184305	FALSE	292231	FALSE	0.969	0.417		3	0.018630434	0.086784521
TRUE	44	rs3910105	SNCA	ENSG00000145335	TRUE	0	TRUE	1.000	3.795		3	0.833159763	0.701174087
FALSE	44	rs3910105	MMRN1	ENSG00000138722	FALSE	43685	FALSE	0.999	1.269		3	0.236753997	0.18473212
FALSE	44	rs3910105	CCSER1	ENSG00000184305	FALSE	291547	FALSE	0.969	0.417		3	0.018630434	0.086784521
TRUE	45	rs356183	SNCA	ENSG00000145335	TRUE	0	TRUE	1.000	3.795		3	0.833159763	0.701174087
FALSE	45	rs356183	MMRN1	ENSG00000138722	FALSE	43402	FALSE	0.999	1.269		3	0.236753997	0.18473212
FALSE	45	rs356183	CCSER1	ENSG00000184305	FALSE	291264	FALSE	0.969	0.417		3	0.018630434	0.086784521
TRUE	46	rs13117519	CAMK2D	ENSG00000145349	TRUE	0	TRUE	0.981	0.475		2	0.80159688	0.801804334
FALSE	46	rs13117519	ANK2	ENSG00000145362	FALSE	63047	FALSE	0.578	0.044		2	0.059084818	0.100870258
TRUE	47	rs62333164	CLCN3	ENSG00000109572	TRUE	0	TRUE	0.999	1.181		3	0.490276244	0.490059215
FALSE	47	rs62333164	NEK1	ENSG00000137601	FALSE	39843	FALSE	0.924	0.308		3	0.384261997	0.461425179
FALSE	47	rs62333164	HPF1	ENSG00000056050	FALSE	76995	FALSE	0.703	0.114		3	0.074857411	
FALSE	48	rs4546327	ERCC8	ENSG00000049167	TRUE	0	FALSE	0.756	0.149		5	0.188361943	0.122081879
FALSE	48	rs4546327	NDUFAF2	ENSG00000164182	FALSE	42795	FALSE	0.642	0.079		5	0.35301614	0.430783044
FALSE	48	rs4546327	ELOVL7	ENSG00000164181	FALSE	58108	TRUE	0.974	0.437		5	0.412941907	0.417738436
FALSE	48	rs4546327	DEPDC1B	ENSG00000035499	FALSE	202192	FALSE	0.158	-0.187		5	0.019766203	0.004642046
FALSE	48	rs4546327	SMIM15	ENSG00000188725	FALSE	255322	FALSE	0.389	-0.057		5	0.008588603	0.004642046
TRUE	49	rs246815	SV2C	ENSG00000122012	TRUE	0	TRUE	0.913	0.291	0.030511632	3		
FALSE	49	rs246815	IQGAP2	ENSG00000145703	FALSE	106960	FALSE	0.004	-0.675		3		
FALSE	49	rs246815	F2RL2	ENSG00000164220	FALSE	319157	FALSE	0.075	-0.275		3		
FALSE	50	rs66872803	ZFYVE16	ENSG00000039319	TRUE	8924	FALSE	0.847	0.218	0.01912433	8		
FALSE	50	rs66872803	SPZ1	ENSG00000164299	FALSE	77247	FALSE	0.152	-0.193		8		
FALSE	50	rs66872803	FAM151B	ENSG00000152380	FALSE	88880	TRUE	0.877	0.245		8		
FALSE	50	rs66872803	SERINC5	ENSG00000164300	FALSE	143041	FALSE	0.551	0.030		8		
FALSE	50	rs66872803	ANKRD34B	ENSG00000189127	FALSE	157666	FALSE	0.056	-0.308		8		
FALSE	50	rs66872803	DHFR	ENSG00000228716	FALSE	227137	FALSE	0.103	-0.239		8		
FALSE	50	rs66872803	MSH3	ENSG00000113318	FALSE	255563	FALSE	0.414	-0.043		8		

FALSE	50	rs66872803	THBS4	ENSG00000113296	FALSE	315798	FALSE	0.678	0.100		8		
FALSE	51	rs26431	PAM	ENSG00000145730	TRUE	28027	FALSE	0.947	0.350		4	0.112282979	0.154538604
FALSE	51	rs26431	GIN1	ENSG00000145723	FALSE	28243	FALSE	0.675	0.098	0.002750503	4	0.573737342	0.382916996
FALSE	51	rs26431	PPIP5K2	ENSG00000145725	FALSE	62392	FALSE	0.805	0.184		4	0.285741095	0.409967089
FALSE	51	rs26431	MACIR	ENSG00000181751	FALSE	201003	TRUE	0.962	0.391		4	0.028238584	
TRUE	52	rs11950533	CAMLG	ENSG00000164615	TRUE	0	TRUE	0.972	0.428	0.05443107	14	0.256455302	0.189410491
FALSE	52	rs11950533	SEC24A	ENSG00000113615	FALSE	12616	FALSE	0.874	0.242		14	0.108639315	0.017473574
FALSE	52	rs11950533	DDX46	ENSG00000145833	FALSE	18246	FALSE	0.877	0.245		14	0.067107342	0.284433832
FALSE	52	rs11950533	SAR1B	ENSG00000152700	FALSE	91254	FALSE	0.888	0.258		14	0.156020477	0.004450453
FALSE	52	rs11950533	C5orf24	ENSG00000181904	FALSE	105155	FALSE	0.925	0.308		14	0.096217245	0.355233703
FALSE	52	rs11950533	TXNDC15	ENSG00000113621	FALSE	133846	FALSE	0.715	0.122		14	0.042531252	0.057179995
FALSE	52	rs11950533	JADE2	ENSG00000043143	FALSE	157295	FALSE	0.716	0.123		14	0.059466109	0.003673071
FALSE	52	rs11950533	PCBD2	ENSG00000132570	FALSE	164595	FALSE	0.812	0.190		14	0.025768291	0.018083978
FALSE	52	rs11950533	CATSPER3	ENSG00000152705	FALSE	227382	FALSE	0.644	0.080		14	0.021993041	0.003705017
FALSE	52	rs11950533	PITX1	ENSG00000069011	FALSE	287209	FALSE	0.941	0.337		14	0.016669136	0.003705017
FALSE	52	rs11950533	CDKN2AIPNL	ENSG00000237190	FALSE	328643	FALSE	0.788	0.172		14	0.009187154	0.003712571
FALSE	52	rs11950533	UBE2B	ENSG00000119048	FALSE	348416	FALSE	0.499	0.002		14	0.008771638	0.006042585
FALSE	52	rs11950533	CDKL3	ENSG00000006837	FALSE	369477	FALSE	0.508	0.007		14	0.009226246	0.003633686
FALSE	52	rs11950533	ENSG00000273345	ENSG00000273345	FALSE	369480	FALSE	NA	NA		14		
FALSE	53	rs4140646	H2BC13	ENSG00000185130	TRUE	99782	FALSE	0.572	0.041		29	0.113072619	
FALSE	53	rs4140646	H2AC13	ENSG00000196747	FALSE	100476	FALSE	0.131	-0.209		29	0.09605784	
FALSE	53	rs4140646	H3C10	ENSG00000278828	FALSE	102354	FALSE	NA	NA		29	0.083572149	
FALSE	53	rs4140646	H2AC14	ENSG00000276368	FALSE	106605	FALSE	NA	NA		29	0.047168296	
FALSE	53	rs4140646	H2BC14	ENSG00000273703	FALSE	107325	FALSE	NA	NA		29	0.070001401	
FALSE	53	rs4140646	H4C11	ENSG00000197238	FALSE	116395	FALSE	0.376	-0.065		29	0.131212309	
FALSE	53	rs4140646	H4C12	ENSG00000273542	FALSE	123477	FALSE	NA	NA		29	0.045706537	
FALSE	53	rs4140646	H2AC15	ENSG00000275221	FALSE	130183	FALSE	NA	NA		29	0.031733941	
FALSE	53	rs4140646	H2BC15	ENSG00000233822	FALSE	130848	TRUE	0.867	0.235		29	0.031733941	
FALSE	53	rs4140646	H2AC16	ENSG00000276903	FALSE	157620	FALSE	NA	NA		29	0.017317476	
FALSE	53	rs4140646	H1-5	ENSG00000184357	FALSE	159095	FALSE	0.597	0.054		29	0.024130423	
FALSE	53	rs4140646	H3C11	ENSG00000275379	FALSE	164148	FALSE	NA	NA		29	0.019619958	
FALSE	53	rs4140646	H4C13	ENSG00000275126	FALSE	165451	FALSE	NA	NA		29	0.019619958	
FALSE	53	rs4140646	H3C12	ENSG00000197153	FALSE	182618	FALSE	0.128	-0.211		29	0.016337676	
FALSE	53	rs4140646	H2AC17	ENSG00000278677	FALSE	185002	FALSE	NA	NA		29	0.016337676	
FALSE	53	rs4140646	H2BC17	ENSG00000274641	FALSE	185728	FALSE	NA	NA		29	0.015986733	
FALSE	53	rs4140646	OR2B2	ENSG00000168131	FALSE	203488	FALSE	0.172	-0.177		29	0.011444523	0.026116672

FALSE	53	rs4140646	ZNF184	ENSG00000096654	FALSE	234578	FALSE	0.207	-0.156		29	0.012302333	0.008622286
FALSE	53	rs4140646	OR2B6	ENSG00000124657	FALSE	249544	FALSE	0.155	-0.189		29	0.012374399	0.007971145
FALSE	53	rs4140646	ZNF391	ENSG00000124613	FALSE	303788	FALSE	0.145	-0.198		29	0.008813804	0.030484478
FALSE	53	rs4140646	OR2B8P	ENSG00000182477	FALSE	345531	FALSE	NA	NA		29		
FALSE	53	rs4140646	ZNF165	ENSG00000197279	FALSE	372871	FALSE	0.348	-0.078		29	0.008382804	0.022300953
FALSE	53	rs4140646	POM121L2	ENSG00000158553	FALSE	395423	FALSE	0.245	-0.134		29	0.007387798	0.016284157
FALSE	53	rs4140646	PRSS16	ENSG00000112812	FALSE	451072	FALSE	0.395	-0.054		29		0.011096242
FALSE	53	rs4140646	H2AC12	ENSG00000274997	FALSE	560134	FALSE	NA	NA		29		
FALSE	53	rs4140646	H2BC12	ENSG00000197903	FALSE	560841	FALSE	0.841	0.212		29		
FALSE	53	rs4140646	H4C9	ENSG00000276180	FALSE	568018	FALSE	NA	NA		29		
FALSE	53	rs4140646	H2AC11	ENSG00000196787	FALSE	574161	FALSE	0.799	0.180		29		
FALSE	53	rs4140646	H2BC11	ENSG00000124635	FALSE	574901	FALSE	0.714	0.121		29		
FALSE	54	rs3132453	HLA-A	ENSG00000206503	TRUE	49684	FALSE	0.212	-0.153		244	0.007332568	0.006699157
FALSE	54	rs3132453	HLA-G	ENSG00000204632	FALSE	60451	FALSE	0.094	-0.249		244	0.006344116	0.005412924
FALSE	54	rs3132453	HLA-F	ENSG00000204642	FALSE	153048	FALSE	0.065	-0.293		244	0.005437217	0.006410174
FALSE	54	rs3132453	POLR1H	ENSG00000066379	FALSE	167323	FALSE	0.485	-0.005		244	0.01200331	
FALSE	54	rs3132453	PPP1R11	ENSG00000204619	FALSE	175133	FALSE	0.678	0.100		244	0.012328765	0.005475942
FALSE	54	rs3132453	RNF39	ENSG00000204618	FALSE	178690	FALSE	0.500	0.003		244	0.019145144	0.008402052
FALSE	54	rs3132453	ZFP57	ENSG00000204644	FALSE	210421	FALSE	0.154	-0.191		244	0.006808795	0.007837236
FALSE	54	rs3132453	TRIM31	ENSG00000204616	FALSE	211321	FALSE	0.298	-0.104		244	0.043425194	0.033640337
FALSE	54	rs3132453	MOG	ENSG00000204655	FALSE	219204	FALSE	0.564	0.036		244	0.004733554	0.005396709
FALSE	54	rs3132453	TRIM40	ENSG00000204614	FALSE	244548	FALSE	0.134	-0.207	0.000186781	244	0.512495851	0.204245976
FALSE	54	rs3132453	GABBR1	ENSG00000204681	FALSE	257600	FALSE	0.959	0.382		244		0.008950594
FALSE	54	rs3132453	TRIM10	ENSG00000204613	FALSE	260367	FALSE	0.260	-0.125		244	0.089177052	0.033637742
FALSE	54	rs3132453	TRIM15	ENSG00000204610	FALSE	271630	FALSE	0.052	-0.318		244	0.155698438	0.033637742
FALSE	54	rs3132453	TRIM26	ENSG00000234127	FALSE	292879	FALSE	0.568	0.039		244	0.044402049	0.01189916
FALSE	54	rs3132453	OR2H2	ENSG00000204657	FALSE	301076	FALSE	0.068	-0.287		244		0.005396709
FALSE	54	rs3132453	UBD	ENSG00000213886	FALSE	331844	FALSE	0.301	-0.103		244		0.005396709
FALSE	54	rs3132453	OR2I1P	ENSG00000237988	FALSE	333803	FALSE	NA	NA		244		
FALSE	54	rs3132453	MAS1L	ENSG00000204687	FALSE	403620	FALSE	0.183	-0.170		244		0.005396709
FALSE	54	rs3132453	OR2H1	ENSG00000204688	FALSE	427248	FALSE	0.138	-0.203		244		0.005396709
FALSE	54	rs3132453	OR11A1	ENSG00000204694	FALSE	434505	FALSE	0.137	-0.205		244		0.005396709
FALSE	54	rs3132453	TRIM39	ENSG00000204599	FALSE	434903	FALSE	0.811	0.189		244	0.011918685	0.018176192
FALSE	54	rs3132453	TRIM39-RPP21	ENSG00000248167	FALSE	437331	FALSE	NA	NA		244	0.015218349	0.009037909
FALSE	54	rs3132453	OR10C1	ENSG00000206474	FALSE	450599	FALSE	0.257	-0.127		244		0.005396709
FALSE	54	rs3132453	RPP21	ENSG00000241370	FALSE	453555	FALSE	0.722	0.127		244	0.013790646	0.02095729

FALSE	54	rs3132453	OR5V1	ENSG00000243729	FALSE	459609	FALSE	0.112	-0.228	244		0.005396709
FALSE	54	rs3132453	OR12D1	ENSG00000251608	FALSE	472671	FALSE	NA	NA	244		
FALSE	54	rs3132453	OR12D2	ENSG00000280236	FALSE	493568	FALSE	NA	NA	244		0.009812798
FALSE	54	rs3132453	OR12D3	ENSG00000112462	FALSE	516285	FALSE	0.143	-0.200	244		0.005396709
FALSE	54	rs3132453	OR14J1	ENSG00000204695	FALSE	578559	FALSE	0.087	-0.258	244		0.005396709
FALSE	54	rs3132453	HLA-E	ENSG00000204592	FALSE	597933	FALSE	0.454	-0.023	244	0.00629883	0.018255627
FALSE	54	rs3132453	GNL1	ENSG00000204590	FALSE	649805	FALSE	0.563	0.035	244	0.006233023	0.009534414
FALSE	54	rs3132453	PRR3	ENSG00000204576	FALSE	665704	FALSE	0.714	0.122	244	0.005365064	0.009472305
FALSE	54	rs3132453	ABCF1	ENSG00000204574	FALSE	679817	FALSE	0.852	0.222	244	0.005995354	0.009625241
FALSE	54	rs3132453	PPP1R10	ENSG00000204569	FALSE	708837	FALSE	0.861	0.231	244	0.005747678	0.01018638
FALSE	54	rs3132453	OR2J2	ENSG00000204700	FALSE	715765	FALSE	0.148	-0.195	244		0.005396709
FALSE	54	rs3132453	MRPS18B	ENSG00000204568	FALSE	726264	FALSE	0.613	0.063	244	0.004274881	0.009472305
FALSE	54	rs3132453	ATAT1	ENSG00000137343	FALSE	735266	FALSE	0.932	0.320	244	0.005825436	0.009472305
FALSE	54	rs3132453	C6orf136	ENSG00000204564	FALSE	755463	FALSE	0.804	0.184	244		0.009573574
FALSE	54	rs3132453	DHX16	ENSG00000204560	FALSE	761543	FALSE	0.640	0.078	244		0.009206463
FALSE	54	rs3132453	OR2J3	ENSG00000204701	FALSE	776806	FALSE	0.073	-0.278	244		
FALSE	54	rs3132453	PPP1R18	ENSG00000146112	FALSE	784813	FALSE	0.846	0.216	244		0.009037909
FALSE	54	rs3132453	OR2J1	ENSG00000204702	FALSE	788875	FALSE	0.278	-0.115	244		
FALSE	54	rs3132453	NRM	ENSG00000137404	FALSE	796471	FALSE	0.647	0.082	244		0.009037909
FALSE	54	rs3132453	OR2B3	ENSG00000204703	FALSE	804263	FALSE	0.154	-0.190	244		
FALSE	54	rs3132453	MDC1	ENSG00000137337	FALSE	808231	FALSE	0.613	0.063	244		0.009166211
FALSE	54	rs3132453	TUBB	ENSG00000196230	FALSE	825859	FALSE	0.641	0.079	244		0.015510071
FALSE	54	rs3132453	FLOT1	ENSG00000137312	FALSE	836133	FALSE	0.829	0.201	244		0.020451814
FALSE	54	rs3132453	OR2W1	ENSG00000204704	FALSE	846401	FALSE	0.216	-0.151	244		
FALSE	54	rs3132453	IER3	ENSG00000137331	FALSE	851623	FALSE	0.086	-0.259	244		0.018704712
FALSE	54	rs3132453	ZNF311	ENSG00000197935	FALSE	886260	FALSE	0.101	-0.241	244		
FALSE	54	rs3132453	TRIM27	ENSG00000204713	FALSE	967588	FALSE	0.825	0.199	244		
FALSE	54	rs3132453	DDR1	ENSG00000204580	FALSE	984845	FALSE	0.790	0.174	244		0.019376768
FALSE	54	rs3132453	GTF2H4	ENSG00000213780	FALSE	1016631	FALSE	0.846	0.216	244		0.009037909
FALSE	54	rs3132453	VARS2	ENSG00000137411	FALSE	1022629	FALSE	0.878	0.245	244		0.017650135
FALSE	54	rs3132453	SFTA2	ENSG00000196260	FALSE	1039777	FALSE	0.636	0.077	244		0.009037909
FALSE	54	rs3132453	MUCL3	ENSG00000168631	FALSE	1042947	FALSE	0.260	-0.125	244		
FALSE	54	rs3132453	MUC21	ENSG00000204544	FALSE	1092142	FALSE	0.156	-0.189	244		0.009037909
FALSE	54	rs3132453	MUC22	ENSG00000261272	FALSE	1118898	FALSE	0.176	-0.174	244		0.009037909
FALSE	54	rs3132453	HCG22	ENSG00000228789	FALSE	1161874	FALSE	NA	NA	244		
FALSE	54	rs3132453	C6orf15	ENSG00000204542	FALSE	1219647	FALSE	0.372	-0.067	244		0.033637742

FALSE	54	rs3132453	PSORS1C1	ENSG00000204540	FALSE	1223174	FALSE	0.450	-0.025		244		0.068681177
FALSE	54	rs3132453	CDSN	ENSG00000204539	FALSE	1223511	FALSE	0.352	-0.076		244		0.033637742
FALSE	54	rs3132453	PSORS1C2	ENSG00000204538	FALSE	1245958	FALSE	0.123	-0.217		244		0.112157023
FALSE	54	rs3132453	CCHCR1	ENSG00000204536	FALSE	1250863	FALSE	0.510	0.008		244		
FALSE	54	rs3132453	TCF19	ENSG00000137310	FALSE	1266755	FALSE	0.230	-0.142		244		
FALSE	54	rs3132453	POU5F1	ENSG00000204531	FALSE	1272761	FALSE	0.386	-0.059		244		
FALSE	54	rs3132453	SCAND3	ENSG00000232040	FALSE	1275364	FALSE	0.273	-0.118		244		0.011923404
FALSE	54	rs3132453	GPX5	ENSG00000224586	FALSE	1356621	FALSE	0.120	-0.221		244		0.007795745
FALSE	54	rs3132453	GPX6	ENSG00000198704	FALSE	1363361	FALSE	0.166	-0.182		244		0.007795745
FALSE	54	rs3132453	HLA-C	ENSG00000204525	FALSE	1377173	FALSE	0.184	-0.170		244		
FALSE	54	rs3132453	ZSCAN23	ENSG00000187987	FALSE	1448074	FALSE	0.270	-0.120		244		0.019057256
FALSE	54	rs3132453	HLA-B	ENSG00000234745	FALSE	1462296	FALSE	0.041	-0.349		244		
FALSE	54	rs3132453	ZSCAN12	ENSG00000158691	FALSE	1491829	FALSE	0.375	-0.066		244		0.011923404
FALSE	54	rs3132453	MICA	ENSG00000204520	FALSE	1508208	FALSE	0.133	-0.208		244		
FALSE	54	rs3132453	ZKSCAN3	ENSG00000189298	FALSE	1522404	FALSE	0.313	-0.096		244		0.017281206
FALSE	54	rs3132453	ZSCAN31	ENSG00000235109	FALSE	1535305	FALSE	0.317	-0.094		244		0.015708035
FALSE	54	rs3132453	PGBD1	ENSG00000137338	FALSE	1589027	FALSE	0.263	-0.124		244		0.022196571
FALSE	54	rs3132453	MICB	ENSG00000204516	FALSE	1603305	FALSE	0.353	-0.075		244		
FALSE	54	rs3132453	ENSG00000276302	ENSG00000276302	FALSE	1609996	FALSE	NA	NA		244		
FALSE	54	rs3132453	ZSCAN26	ENSG00000197062	FALSE	1613352	FALSE	NA	NA		244	0.011245064	
FALSE	54	rs3132453	NKAPL	ENSG00000189134	FALSE	1630617	FALSE	0.586	0.048		244	0.008251852	0.008467144
FALSE	54	rs3132453	MCCD1	ENSG00000204511	FALSE	1637386	FALSE	0.145	-0.198		244		
FALSE	54	rs3132453	DDX39B	ENSG00000198563	FALSE	1638643	FALSE	0.769	0.160		244		
FALSE	54	rs3132453	ATP6V1G2-DDX39B	ENSG00000254870	FALSE	1638643	FALSE	NA	NA		244		
FALSE	54	rs3132453	ZKSCAN4	ENSG00000187626	FALSE	1639306	FALSE	0.545	0.027		244	0.007356829	0.013002361
FALSE	54	rs3132453	ATP6V1G2	ENSG00000213760	FALSE	1652868	FALSE	0.535	0.021		244		
FALSE	54	rs3132453	NFKBIL1	ENSG00000204498	FALSE	1655294	FALSE	0.749	0.144	0.009159415	244		
FALSE	54	rs3132453	ZSCAN9	ENSG00000137185	FALSE	1658088	FALSE	0.379	-0.063		244	0.010296515	0.014234562
FALSE	54	rs3132453	LTA	ENSG00000226979	FALSE	1680478	FALSE	0.299	-0.104		244		
FALSE	54	rs3132453	TNF	ENSG00000232810	FALSE	1683989	FALSE	0.415	-0.042		244		
FALSE	54	rs3132453	LTB	ENSG00000227507	FALSE	1688949	FALSE	0.186	-0.168		244		
FALSE	54	rs3132453	LST1	ENSG00000204482	FALSE	1694548	FALSE	0.380	-0.062		244		
FALSE	54	rs3132453	NCR3	ENSG00000204475	FALSE	1697319	FALSE	0.084	-0.262		244		
FALSE	54	rs3132453	ZKSCAN8P1	ENSG00000226314	FALSE	1721981	FALSE	NA	NA		244	0.005550133	
FALSE	54	rs3132453	AIF1	ENSG00000204472	FALSE	1723641	TRUE	0.979	0.463		244		0.004125259
FALSE	54	rs3132453	PRRC2A	ENSG00000204469	FALSE	1729139	FALSE	0.941	0.338	0.163304966	244		0.008151822

FALSE	54	rs3132453	ENSG00000291302	ENSG00000291302	FALSE	1731198	FALSE	NA	NA		244		
FALSE	54	rs3132453	ZKSCAN8	ENSG00000198315	FALSE	1732115	FALSE	0.682	0.102		244	0.007429547	0.023342675
FALSE	54	rs3132453	ENSG00000289282	ENSG00000289282	FALSE	1732298	FALSE	NA	NA		244		
FALSE	54	rs3132453	BAG6	ENSG00000204463	FALSE	1747452	FALSE	0.801	0.182		244		0.008151822
FALSE	54	rs3132453	APOM	ENSG00000204444	FALSE	1760840	FALSE	0.624	0.070		244		0.004125259
FALSE	54	rs3132453	ZSCAN16	ENSG00000196812	FALSE	1761493	FALSE	0.403	-0.049		244	0.008225856	0.018240722
FALSE	54	rs3132453	C6orf47	ENSG00000204439	FALSE	1766722	FALSE	0.499	0.002		244		0.004125259
FALSE	54	rs3132453	GPANK1	ENSG00000204438	FALSE	1769652	FALSE	0.765	0.157		244		0.004152467
FALSE	54	rs3132453	CSNK2B	ENSG00000204435	FALSE	1773651	FALSE	0.546	0.027		244		0.004220959
FALSE	54	rs3132453	ENSG00000263020	ENSG00000263020	FALSE	1774526	FALSE	NA	NA		244		
FALSE	54	rs3132453	LY6G5B	ENSG00000240053	FALSE	1778400	FALSE	0.623	0.069		244		0.006206293
FALSE	54	rs3132453	LY6G5C	ENSG00000204428	FALSE	1785108	FALSE	0.401	-0.051		244		0.006329166
FALSE	54	rs3132453	ABHD16A	ENSG00000204427	FALSE	1795379	FALSE	0.832	0.204		244		0.004125259
FALSE	54	rs3132453	ZNF165	ENSG00000197279	FALSE	1802012	FALSE	0.348	-0.078		244	0.008382804	0.022300953
FALSE	54	rs3132453	LY6G6F	ENSG00000204424	FALSE	1815290	FALSE	0.457	-0.021		244		0.004125259
FALSE	54	rs3132453	LY6G6F-LY6G6D	ENSG00000250641	FALSE	1815328	FALSE	0.546	0.027		244		
FALSE	54	rs3132453	LY6G6D	ENSG00000244355	FALSE	1823772	FALSE	0.329	-0.088		244		0.004125259
FALSE	54	rs3132453	MPIG6B	ENSG00000204420	FALSE	1827018	FALSE	0.725	0.128		244		
FALSE	54	rs3132453	LY6G6C	ENSG00000204421	FALSE	1827072	FALSE	0.128	-0.212		244		0.007158998
FALSE	54	rs3132453	DDAH2	ENSG00000213722	FALSE	1835462	FALSE	0.463	-0.017		244		0.004901233
FALSE	54	rs3132453	OR2B8P	ENSG00000182477	FALSE	1837410	FALSE	NA	NA		244		
FALSE	54	rs3132453	CLIC1	ENSG00000213719	FALSE	1839005	FALSE	0.145	-0.198		244		0.004125259
FALSE	54	rs3132453	MSH5	ENSG00000204410	FALSE	1848101	FALSE	0.578	0.044		244		0.004125259
FALSE	54	rs3132453	MSH5-SAPCD1	ENSG00000255152	FALSE	1848444	FALSE	NA	NA		244		0.004125259
FALSE	54	rs3132453	SAPCD1	ENSG00000228727	FALSE	1871080	FALSE	0.292	-0.108		244		0.004125259
FALSE	54	rs3132453	VWA7	ENSG00000204396	FALSE	1874014	FALSE	0.464	-0.017		244		0.007158998
FALSE	54	rs3132453	VARS1	ENSG00000204394	FALSE	1885942	FALSE	0.958	0.379		244		
FALSE	54	rs3132453	LSM2	ENSG00000204392	FALSE	1905820	FALSE	0.755	0.148		244		0.004116149
FALSE	54	rs3132453	HSPA1L	ENSG00000204390	FALSE	1918043	FALSE	0.830	0.202		244		0.006643702
FALSE	54	rs3132453	HSPA1A	ENSG00000204389	FALSE	1923967	FALSE	0.817	0.194		244		0.009229966
FALSE	54	rs3132453	OR2B6	ENSG00000124657	FALSE	1933393	FALSE	0.155	-0.189		244	0.012374399	0.007971145
FALSE	54	rs3132453	HSPA1B	ENSG00000204388	FALSE	1936162	FALSE	0.926	0.309		244		0.012294512
FALSE	54	rs3132453	NEU1	ENSG00000204386	FALSE	1966083	FALSE	0.864	0.233		244		0.03152073
FALSE	54	rs3132453	SLC44A4	ENSG00000204385	FALSE	1971616	FALSE	0.146	-0.197		244		0.140762703
FALSE	54	rs3132453	OR2B2	ENSG00000168131	FALSE	1979179	FALSE	0.172	-0.177		244	0.011444523	0.026116672
FALSE	54	rs3132453	EHMT2	ENSG00000204371	FALSE	1988183	FALSE	0.631	0.073		244		0.148025098

FALSE	54	rs3132453	H2BC17	ENSG00000274641	FALSE	1997684	FALSE	NA	NA	244	0.015986733	
FALSE	54	rs3132453	H2AC17	ENSG00000278677	FALSE	1998390	FALSE	NA	NA	244	0.016337676	
FALSE	54	rs3132453	H3C12	ENSG00000197153	FALSE	1998469	FALSE	0.128	-0.211	244	0.016337676	
FALSE	54	rs3132453	C2	ENSG00000166278	FALSE	2006209	FALSE	0.702	0.113	244		0.035140718
FALSE	54	rs3132453	ZBTB12	ENSG00000204366	FALSE	2008037	FALSE	0.260	-0.125	244		0.014854675
FALSE	54	rs3132453	H4C13	ENSG00000275126	FALSE	2018041	FALSE	NA	NA	244	0.019619958	
FALSE	54	rs3132453	H3C11	ENSG00000275379	FALSE	2019229	FALSE	NA	NA	244	0.019619958	
FALSE	54	rs3132453	H1-5	ENSG00000184357	FALSE	2023987	FALSE	0.597	0.054	244	0.024130423	
FALSE	54	rs3132453	H2AC16	ENSG00000276903	FALSE	2025777	FALSE	NA	NA	244	0.017317476	
FALSE	54	rs3132453	H2BC15	ENSG00000233822	FALSE	2035866	FALSE	0.867	0.235	244	0.031733941	
FALSE	54	rs3132453	ENSG00000244255	ENSG00000244255	FALSE	2036122	FALSE	0.087	-0.258	244		
FALSE	54	rs3132453	H2AC15	ENSG00000275221	FALSE	2053200	FALSE	NA	NA	244	0.031733941	
FALSE	54	rs3132453	CFB	ENSG00000243649	FALSE	2054074	FALSE	0.201	-0.160	244		0.006408368
FALSE	54	rs3132453	H4C12	ENSG00000273542	FALSE	2060015	FALSE	NA	NA	244	0.045706537	
FALSE	54	rs3132453	NELFE	ENSG00000204356	FALSE	2060511	FALSE	0.909	0.285	244		0.004713639
FALSE	54	rs3132453	H4C11	ENSG00000197238	FALSE	2067095	FALSE	0.376	-0.065	244	0.131212309	
FALSE	54	rs3132453	SKIC2	ENSG00000204351	FALSE	2067540	FALSE	0.772	0.161	244		
FALSE	54	rs3132453	H2BC14	ENSG00000273703	FALSE	2076086	FALSE	NA	NA	244	0.070001401	
FALSE	54	rs3132453	H2AC14	ENSG00000276368	FALSE	2076798	FALSE	NA	NA	244	0.047168296	
FALSE	54	rs3132453	DXO	ENSG00000204348	FALSE	2078234	FALSE	0.600	0.056	244		0.004116149
FALSE	54	rs3132453	STK19	ENSG00000204344	FALSE	2079515	FALSE	0.805	0.185	244		0.004116149
FALSE	54	rs3132453	H3C10	ENSG00000278828	FALSE	2080275	FALSE	NA	NA	244	0.083572149	
FALSE	54	rs3132453	H2AC13	ENSG00000196747	FALSE	2082908	FALSE	0.131	-0.209	244	0.09605784	
FALSE	54	rs3132453	H2BC13	ENSG00000185130	FALSE	2083646	FALSE	0.572	0.041	244	0.113072619	
FALSE	54	rs3132453	C4A	ENSG00000244731	FALSE	2090481	FALSE	0.739	0.138	244		0.004298489
FALSE	54	rs3132453	AL645922.1	ENSG00000268923	FALSE	2114592	FALSE	NA	NA	244		
FALSE	54	rs3132453	C4B	ENSG00000224389	FALSE	2123219	FALSE	0.849	0.219	244		0.004298489
FALSE	54	rs3132453	CYP21A2	ENSG00000231852	FALSE	2146751	FALSE	0.377	-0.064	244		0.006139348
FALSE	54	rs3132453	TNXB	ENSG00000168477	FALSE	2149577	FALSE	0.705	0.115	244	0.005596526	0.007115453
FALSE	54	rs3132453	ATF6B	ENSG00000213676	FALSE	2223688	FALSE	0.774	0.163	244	0.004556763	0.008133956
FALSE	54	rs3132453	FKBPL	ENSG00000204315	FALSE	2237131	FALSE	0.503	0.004	244	0.004603885	0.004116149
FALSE	54	rs3132453	PRRT1	ENSG00000204314	FALSE	2256783	FALSE	0.825	0.199	244	0.005726768	0.004690704
FALSE	54	rs3132453	ENSG00000285085	ENSG00000285085	FALSE	2258919	FALSE	NA	NA	244		
FALSE	54	rs3132453	PPT2	ENSG00000221988	FALSE	2261865	FALSE	0.897	0.270	244	0.004556763	0.007143279
FALSE	54	rs3132453	PPT2-EGFL8	ENSG00000258388	FALSE	2262269	FALSE	NA	NA	244	0.005030693	0.004160764
FALSE	54	rs3132453	EGFL8	ENSG00000241404	FALSE	2273019	FALSE	0.972	0.430	244	0.005030693	0.007143279

FALSE	54	rs3132453	AGPAT1	ENSG00000204310	FALSE	2276636	FALSE	0.906	0.281		244	0.005173083	0.008221458
FALSE	54	rs3132453	RNF5	ENSG00000204308	FALSE	2286829	FALSE	0.646	0.082		244	0.005030693	0.004160764
FALSE	54	rs3132453	AGER	ENSG00000204305	FALSE	2289392	FALSE	0.667	0.094		244	0.005030693	0.007220265
FALSE	54	rs3132453	PBX2	ENSG00000204304	FALSE	2293157	FALSE	0.481	-0.007		244	0.005212816	0.007220265
FALSE	54	rs3132453	GPSM3	ENSG00000213654	FALSE	2299190	FALSE	0.510	0.008		244	0.006783693	0.004160764
FALSE	54	rs3132453	NOTCH4	ENSG00000204301	FALSE	2303267	FALSE	0.831	0.203		244	0.005030693	0.007220265
FALSE	54	rs3132453	TSBP1	ENSG00000204296	FALSE	2396950	FALSE	0.125	-0.216		244	0.007443277	
FALSE	54	rs3132453	ZNF184	ENSG00000096654	FALSE	2418456	FALSE	0.207	-0.156		244	0.012302333	0.008622286
FALSE	54	rs3132453	ZNF391	ENSG00000124613	FALSE	2487666	FALSE	0.145	-0.198		244	0.008813804	0.030484478
FALSE	54	rs3132453	BTNL2	ENSG00000204290	FALSE	2501763	FALSE	0.111	-0.229		244	0.012115163	0.004196952
FALSE	54	rs3132453	HLA-DRA	ENSG00000204287	FALSE	2548302	FALSE	0.109	-0.232		244	0.016996205	0.007626992
FALSE	54	rs3132453	POM121L2	ENSG00000158553	FALSE	2579301	FALSE	0.245	-0.134		244	0.007387798	0.016284157
FALSE	54	rs3132453	HLA-DRB5	ENSG00000198502	FALSE	2625777	FALSE	0.140	-0.202		244	0.204653487	0.006774994
FALSE	54	rs3132453	PRSS16	ENSG00000112812	FALSE	2634950	FALSE	0.395	-0.054		244		0.011096242
FALSE	54	rs3132453	HLA-DRB1	ENSG00000196126	FALSE	2686326	FALSE	0.342	-0.082		244	0.033857968	0.11221558
FALSE	54	rs3132453	HLA-DQA1	ENSG00000196735	FALSE	2736603	FALSE	0.167	-0.181		244	0.287814587	0.036825269
FALSE	54	rs3132453	H2AC12	ENSG00000274997	FALSE	2744012	FALSE	NA	NA		244		
FALSE	54	rs3132453	H2BC12	ENSG00000197903	FALSE	2744719	FALSE	0.841	0.212		244		
FALSE	54	rs3132453	H4C9	ENSG00000276180	FALSE	2751896	FALSE	NA	NA		244		
FALSE	54	rs3132453	H2AC11	ENSG00000196787	FALSE	2758039	FALSE	0.799	0.180		244		
FALSE	54	rs3132453	H2BC11	ENSG00000124635	FALSE	2758779	FALSE	0.714	0.121		244		
FALSE	54	rs3132453	HLA-DQB1	ENSG00000179344	FALSE	2767891	FALSE	0.176	-0.175		244	0.037998568	0.007116397
FALSE	54	rs3132453	HLA-DQA2	ENSG00000237541	FALSE	2849815	FALSE	0.477	-0.010		244	0.016019372	0.004470911
FALSE	54	rs3132453	HLA-DQB2	ENSG00000232629	FALSE	2864522	FALSE	0.189	-0.167		244	0.014078485	0.004451132
FALSE	54	rs3132453	HLA-DOB	ENSG00000241106	FALSE	2921187	FALSE	0.040	-0.353		244	0.008545809	0.006259503
FALSE	54	rs3132453	ENSG00000250264	ENSG00000250264	FALSE	2922191	FALSE	0.198	-0.162		244		
FALSE	54	rs3132453	TAP2	ENSG00000204267	FALSE	2930257	FALSE	0.125	-0.214		244	0.009682467	0.006088336
FALSE	54	rs3132453	PSMB8	ENSG00000204264	FALSE	2949141	FALSE	0.797	0.178		244	0.009477983	0.004160764
FALSE	54	rs3132453	PSMB9	ENSG00000240065	FALSE	2952560	FALSE	0.280	-0.114		244	0.009623622	0.005950848
FALSE	54	rs3132453	TAP1	ENSG00000168394	FALSE	2953633	FALSE	0.639	0.078		244	0.009742906	0.007408089
FALSE	54	rs3132453	HLA-DMB	ENSG00000242574	FALSE	3043053	FALSE	0.045	-0.339		244	0.005852942	0.004116149
FALSE	54	rs3132453	ENSG00000248993	ENSG00000248993	FALSE	3045788	FALSE	NA	NA		244		
FALSE	54	rs3132453	HLA-DMA	ENSG00000204257	FALSE	3057037	FALSE	0.939	0.333		244	0.005723147	0.004174025
FALSE	54	rs3132453	BRD2	ENSG00000204256	FALSE	3077018	FALSE	0.933	0.323		244	0.005127585	0.004690704
FALSE	54	rs3132453	ZNF322	ENSG00000181315	FALSE	3199373	FALSE	0.561	0.034		244		
FALSE	54	rs3132453	ABT1	ENSG00000146109	FALSE	3258386	FALSE	0.431	-0.034		244		

FALSE	54	rs3132453	HMGN4	ENSG00000182952	FALSE	3312192	FALSE	0.486	-0.005	244
FALSE	54	rs3132453	BTN1A1	ENSG00000124557	FALSE	3348700	FALSE	0.064	-0.294	244
FALSE	54	rs3132453	BTN2A1	ENSG00000112763	FALSE	3382504	FALSE	0.408	-0.046	244
FALSE	54	rs3132453	BTN3A3	ENSG00000111801	FALSE	3405710	FALSE	0.152	-0.192	244
FALSE	54	rs3132453	BTN3A1	ENSG00000026950	FALSE	3443917	FALSE	0.213	-0.152	244
FALSE	54	rs3132453	BTN2A2	ENSG00000124508	FALSE	3464251	FALSE	0.143	-0.199	244
FALSE	54	rs3132453	BTN3A2	ENSG00000186470	FALSE	3480805	FALSE	0.079	-0.270	244
FALSE	54	rs3132453	H4C8	ENSG00000158406	FALSE	3573487	FALSE	0.417	-0.042	244
FALSE	54	rs3132453	H2BC10	ENSG00000278588	FALSE	3585713	FALSE	NA	NA	244
FALSE	54	rs3132453	H3C8	ENSG00000273983	FALSE	3587712	FALSE	NA	NA	244
FALSE	54	rs3132453	H2BC9	ENSG00000275713	FALSE	3607050	FALSE	NA	NA	244
FALSE	54	rs3132453	H3C7	ENSG00000277775	FALSE	3608490	FALSE	NA	NA	244
FALSE	54	rs3132453	H4C7	ENSG00000275663	FALSE	3612129	FALSE	NA	NA	244
FALSE	54	rs3132453	H1-3	ENSG00000124575	FALSE	3624138	FALSE	0.288	-0.110	244
FALSE	54	rs3132453	H3C6	ENSG00000274750	FALSE	3631652	FALSE	NA	NA	244
FALSE	54	rs3132453	H2AC8	ENSG00000277075	FALSE	3641688	FALSE	NA	NA	244
FALSE	54	rs3132453	H4C5	ENSG00000276966	FALSE	3654104	FALSE	NA	NA	244
FALSE	54	rs3132453	H2AC7	ENSG00000196866	FALSE	3659832	FALSE	0.884	0.252	244
FALSE	54	rs3132453	ENSG00000282988	ENSG00000282988	FALSE	3659832	FALSE	NA	NA	244
FALSE	54	rs3132453	H3C4	ENSG00000197409	FALSE	3661839	FALSE	0.342	-0.082	244
FALSE	54	rs3132453	H4C4	ENSG00000277157	FALSE	3670013	FALSE	NA	NA	244
FALSE	54	rs3132453	H2BC6	ENSG00000274290	FALSE	3674470	FALSE	NA	NA	244
FALSE	54	rs3132453	H2BC5	ENSG00000158373	FALSE	3687776	FALSE	0.948	0.352	244
FALSE	54	rs3132453	H1-4	ENSG00000168298	FALSE	3702010	FALSE	0.838	0.209	244
FALSE	54	rs3132453	H2AC6	ENSG00000180573	FALSE	3720009	FALSE	0.928	0.312	244
FALSE	54	rs3132453	H2BC4	ENSG00000180596	FALSE	3735199	FALSE	0.711	0.120	244
FALSE	54	rs3132453	H1-6	ENSG00000187475	FALSE	3750990	FALSE	0.405	-0.048	244
FALSE	54	rs3132453	H4C3	ENSG00000197061	FALSE	3754788	FALSE	0.525	0.016	244
FALSE	54	rs3132453	HFE	ENSG00000010704	FALSE	3760782	FALSE	0.704	0.115	244
FALSE	54	rs3132453	H1-2	ENSG00000187837	FALSE	3802655	FALSE	0.313	-0.096	244
FALSE	54	rs3132453	H3C3	ENSG00000287080	FALSE	3813256	FALSE	NA	NA	244
FALSE	54	rs3132453	H2BC3	ENSG00000276410	FALSE	3815412	FALSE	NA	NA	244
FALSE	54	rs3132453	H2AC4	ENSG00000278463	FALSE	3825507	FALSE	NA	NA	244
FALSE	54	rs3132453	H3C2	ENSG00000286522	FALSE	3827026	FALSE	NA	NA	244
FALSE	54	rs3132453	HIST1H4B	ENSG00000124529	FALSE	3831873	FALSE	0.417	-0.042	244
FALSE	54	rs3132453	H4C1	ENSG00000278637	FALSE	3837075	FALSE	NA	NA	244

FALSE	54	rs3132453	H3C1	ENSG00000275714	FALSE	3838167	FALSE	NA	NA		244		
FALSE	54	rs3132453	H1-1	ENSG00000124610	FALSE	3841338	FALSE	0.555	0.032		244		
FALSE	54	rs3132453	TRIM38	ENSG00000112343	FALSE	3867894	FALSE	0.129	-0.210		244		
FALSE	54	rs3132453	SLC17A2	ENSG00000112337	FALSE	3928434	FALSE	0.065	-0.292		244		
FALSE	55	rs112485576	HLA-DQA1	ENSG00000196735	TRUE	17184	FALSE	0.167	-0.181		15	0.287814587	0.036825269
FALSE	55	rs112485576	HLA-DRB1	ENSG00000196126	FALSE	21147	FALSE	0.342	-0.082		15	0.033857968	0.11221558
FALSE	55	rs112485576	HLA-DQB1	ENSG00000179344	FALSE	48472	FALSE	0.176	-0.175		15	0.037998568	0.007116397
FALSE	55	rs112485576	HLA-DRB5	ENSG00000198502	FALSE	80708	FALSE	0.140	-0.202		15	0.204653487	0.006774994
FALSE	55	rs112485576	HLA-DQA2	ENSG00000237541	FALSE	130396	FALSE	0.477	-0.010		15	0.016019372	0.004470911
FALSE	55	rs112485576	HLA-DQB2	ENSG00000232629	FALSE	145103	FALSE	0.189	-0.167		15	0.014078485	0.004451132
FALSE	55	rs112485576	HLA-DRA	ENSG00000204287	FALSE	165949	FALSE	0.109	-0.232		15	0.016996205	0.007626992
FALSE	55	rs112485576	HLA-DOB	ENSG00000241106	FALSE	201768	FALSE	0.040	-0.353		15	0.008545809	0.006259503
FALSE	55	rs112485576	ENSG00000250264	ENSG00000250264	FALSE	202772	FALSE	0.198	-0.162		15		
FALSE	55	rs112485576	BTNL2	ENSG00000204290	FALSE	203814	FALSE	0.111	-0.229		15	0.012115163	0.004196952
FALSE	55	rs112485576	TAP2	ENSG00000204267	FALSE	210838	FALSE	0.125	-0.214		15	0.009682467	0.006088336
FALSE	55	rs112485576	PSMB8	ENSG00000204264	FALSE	229722	TRUE	0.797	0.178		15	0.009477983	0.004160764
FALSE	55	rs112485576	PSMB9	ENSG00000240065	FALSE	233141	FALSE	0.280	-0.114		15	0.009623622	0.005950848
FALSE	55	rs112485576	TAP1	ENSG00000168394	FALSE	234214	FALSE	0.639	0.078		15	0.009742906	0.007408089
FALSE	55	rs112485576	TSBP1	ENSG00000204296	FALSE	239088	FALSE	0.125	-0.216		15	0.007443277	
TRUE	56	rs12528068	RIMS1	ENSG00000079841	TRUE	106902	TRUE	0.982	0.480		1	0.631680191	0.30885652
TRUE	57	rs9487736	FYN	ENSG00000010810	TRUE	0	TRUE	1.000	2.933		7	0.788654149	0.711094171
FALSE	57	rs9487736	CCN6	ENSG00000112761	FALSE	203649	FALSE	0.476	-0.010		7	0.034936287	
FALSE	57	rs9487736	TUBE1	ENSG00000074935	FALSE	220237	FALSE	0.640	0.079		7	0.021474894	0.101107448
FALSE	57	rs9487736	FAM229B	ENSG00000203778	FALSE	237165	FALSE	0.417	-0.042		7	0.041279223	0.045482768
FALSE	57	rs9487736	TRAF3IP2	ENSG00000056972	FALSE	243520	FALSE	0.796	0.178		7	0.010880588	0.016863011
FALSE	57	rs9487736	LAMA4	ENSG00000112769	FALSE	257505	FALSE	0.831	0.203		7	0.019501474	0.010465384
FALSE	57	rs9487736	ENSG00000281613	ENSG00000281613	FALSE	386378	FALSE	NA	NA		7		
TRUE	58	rs41286192	SLC18B1	ENSG00000146409	FALSE	8983	FALSE	0.669	0.095	0.725891173	11	0.420924573	0.060336806
FALSE	58	rs41286192	RPS12	ENSG00000112306	TRUE	4758	TRUE	0.951	0.362		11	0.318278948	0.102514796
FALSE	58	rs41286192	VNN2	ENSG00000112303	FALSE	58863	FALSE	0.048	-0.332		11	0.088860619	0.05328813
FALSE	58	rs41286192	VNN1	ENSG00000112299	FALSE	108267	FALSE	0.352	-0.076		11	0.059150025	0.05328813
FALSE	58	rs41286192	TAAR1	ENSG00000146399	FALSE	163140	FALSE	0.105	-0.238		11	0.023500163	0.03328507
FALSE	58	rs41286192	TAAR2	ENSG00000146378	FALSE	198047	FALSE	0.045	-0.338		11	0.014503499	0.03300539
FALSE	58	rs41286192	TAAR5	ENSG00000135569	FALSE	232581	FALSE	0.067	-0.289		11	0.012220667	0.03300539
FALSE	58	rs41286192	TAAR6	ENSG00000146383	FALSE	250963	FALSE	0.315	-0.095		11	0.012220667	0.03300539
FALSE	58	rs41286192	TAAR8	ENSG00000146385	FALSE	268566	FALSE	0.168	-0.181		11	0.011235971	0.010744601

FALSE	58	rs41286192	TAAR9	ENSG00000237110	FALSE	282986	FALSE	NA	NA	11	0.010295437	
FALSE	58	rs41286192	STX7	ENSG00000079950	FALSE	309124	FALSE	0.591	0.051	11	0.008890722	0.027834853
FALSE	59	rs199351	GPNMB	ENSG00000136235	TRUE	6850	FALSE	0.764	0.155	7	0.759734999	0.405959284
FALSE	59	rs199351	NUP42	ENSG00000136243	FALSE	28106	FALSE	0.771	0.161	7	0.068042115	
FALSE	59	rs199351	KLHL7	ENSG00000122550	FALSE	51203	FALSE	0.776	0.164	7	0.02481896	0.013011828
FALSE	59	rs199351	MALSU1	ENSG00000156928	FALSE	69622	FALSE	0.262	-0.124	7	0.060563061	0.462830158
FALSE	59	rs199351	IGF2BP3	ENSG00000136231	FALSE	81092	FALSE	0.759	0.152	7	0.021472649	0.00716216
FALSE	59	rs199351	HYCC1	ENSG00000122591	FALSE	214987	TRUE	0.978	0.458	7		
FALSE	59	rs199351	TRA2A	ENSG00000164548	FALSE	275663	FALSE	0.870	0.238	7	0.011363712	0.012357674
FALSE	60	rs76949143	KCTD7	ENSG00000243335	TRUE	4290	FALSE	0.338	-0.084	12	0.13676051	0.655707303
FALSE	60	rs76949143	ENSG00000284461	ENSG00000284461	FALSE	4367	FALSE	NA	NA	12		
FALSE	60	rs76949143	RABGEF1	ENSG00000154710	FALSE	57573	TRUE	0.963	0.396	12	0.306010273	0.05020455
FALSE	60	rs76949143	TPST1	ENSG00000169902	FALSE	204048	FALSE	0.946	0.347	12	0.064282147	0.019227229
FALSE	60	rs76949143	TMEM248	ENSG00000106609	FALSE	296634	FALSE	0.775	0.163	12	0.169882131	0.044274278
FALSE	60	rs76949143	SBDS	ENSG00000126524	FALSE	363089	FALSE	0.914	0.293	12	0.054831735	0.036462515
FALSE	60	rs76949143	TYW1	ENSG00000198874	FALSE	370582	FALSE	0.681	0.102	12	0.020284757	0.132900395
FALSE	60	rs76949143	CRCP	ENSG00000241258	FALSE	470023	FALSE	0.663	0.091	12	0.092726861	0.018633919
FALSE	60	rs76949143	ENSG00000249319	ENSG00000249319	FALSE	472314	FALSE	NA	NA	12		
FALSE	60	rs76949143	ASL	ENSG00000126522	FALSE	529894	FALSE	0.695	0.110	12	0.155221586	0.010944355
FALSE	60	rs76949143	GUSB	ENSG00000169919	FALSE	642376	FALSE	0.592	0.052	12		0.010944355
FALSE	60	rs76949143	VKORC1L1	ENSG00000196715	FALSE	665028	FALSE	0.472	-0.012	12		0.020701102
TRUE	61	rs1293298	CTSB	ENSG00000164733	TRUE	0	TRUE	0.998	0.875	13	0.604341209	0.499329112
FALSE	61	rs1293298	FDFT1	ENSG00000079459	FALSE	8633	FALSE	0.866	0.235	13	0.155188531	0.416765838
FALSE	61	rs1293298	NEIL2	ENSG00000154328	FALSE	60683	FALSE	0.889	0.259	13	0.027852776	0.004415948
FALSE	61	rs1293298	C8orf49	ENSG00000255394	FALSE	85228	FALSE	0.040	-0.351	13		0.004176373
FALSE	61	rs1293298	GATA4	ENSG00000136574	FALSE	88026	FALSE	0.777	0.165	13	0.019138608	0.004176373
FALSE	61	rs1293298	DEFB136	ENSG00000205884	FALSE	125909	FALSE	0.233	-0.141	13	0.018586554	0.004176373
FALSE	61	rs1293298	DEFB135	ENSG00000205883	FALSE	134228	FALSE	0.137	-0.205	13	0.017610963	0.004176373
FALSE	61	rs1293298	DEFB134	ENSG00000205882	FALSE	145146	FALSE	0.106	-0.236	13	0.021766515	0.004176373
FALSE	61	rs1293298	DEFB130B	ENSG00000233050	FALSE	216361	FALSE	0.311	-0.097	13	0.011655509	
FALSE	61	rs1293298	ZNF705D	ENSG00000215343	FALSE	241310	FALSE	0.162	-0.184	13	0.011408499	0.004176373
FALSE	61	rs1293298	BLK	ENSG00000136573	FALSE	283429	FALSE	0.973	0.435	13	0.009919639	0.004337192
FALSE	61	rs1293298	USP17L7	ENSG00000226430	FALSE	284389	FALSE	NA	NA	13	0.008971703	
FALSE	61	rs1293298	USP17L2	ENSG00000223443	FALSE	288407	FALSE	0.172	-0.178	13	0.008971703	0.004140367
FALSE	62	rs34096562	FGF20	ENSG00000078579	TRUE	149596	FALSE	0.381	-0.062	3	0.474293739	0.062917694
FALSE	62	rs34096562	MICU3	ENSG00000155970	FALSE	184653	TRUE	0.648	0.083	3	0.349697668	0.21418424

FALSE	62	rs34096562	MSR1	ENSG00000038945	FALSE	275095	FALSE	0.139	-0.203	3	0.066691148	0.038316887
FALSE	63	rs3736147	BIN3	ENSG00000147439	TRUE	0	FALSE	0.582	0.046	11	0.24293164	0.391558598
FALSE	63	rs3736147	CCAR2	ENSG00000158941	FALSE	2138	FALSE	0.763	0.155	11	0.293493417	0.147188227
FALSE	63	rs3736147	C8orf58	ENSG00000241852	FALSE	18952	FALSE	0.531	0.019	11	0.224362984	0.067419149
FALSE	63	rs3736147	ENSG00000248235	ENSG00000248235	FALSE	21018	FALSE	NA	NA	11		
FALSE	63	rs3736147	PDLIM2	ENSG00000120913	FALSE	25077	FALSE	0.895	0.267	11	0.106192129	0.083480884
FALSE	63	rs3736147	SORBS3	ENSG00000120896	FALSE	47314	FALSE	0.812	0.189	11	0.02089064	0.074806075
FALSE	63	rs3736147	EGR3	ENSG00000179388	FALSE	64557	FALSE	0.435	-0.032	11	0.023222272	0.020824005
FALSE	63	rs3736147	PPP3CC	ENSG00000120910	FALSE	81960	FALSE	0.849	0.219	11	0.010468462	0.011596718
FALSE	63	rs3736147	PEBP4	ENSG00000134020	FALSE	90149	FALSE	0.559	0.033	11	0.012009481	0.006592559
FALSE	63	rs3736147	SLC39A14	ENSG00000104635	FALSE	188973	TRUE	0.942	0.340	11	0.007701301	0.00744454
FALSE	63	rs3736147	PIWIL2	ENSG00000197181	FALSE	265534	FALSE	0.833	0.205	11	0.006988178	0.006592559
TRUE	64	rs13294100	SH3GL2	ENSG00000107295	TRUE	0	TRUE	1.000	1.699	2	0.854108691	0.9623985
FALSE	64	rs13294100	CNTLN	ENSG00000044459	FALSE	76588	FALSE	0.296	-0.106	2	0.019283784	0.013154971
TRUE	65	rs10756905	SH3GL2	ENSG00000107295	TRUE	0	TRUE	1.000	1.699	3	0.854108691	0.9623985
FALSE	65	rs10756905	ADAMTSL1	ENSG00000178031	FALSE	180392	FALSE	0.507	0.006	3	0.167852784	0.007662146
FALSE	65	rs10756905	CNTLN	ENSG00000044459	FALSE	222248	FALSE	0.296	-0.106	3	0.019283784	0.013154971
TRUE	66	rs6476434	UBAP2	ENSG00000137073	TRUE	0	TRUE	0.917	0.298	8	0.198334977	0.738002728
FALSE	66	rs6476434	UBE2R2	ENSG00000107341	FALSE	69257	FALSE	0.881	0.248	8	0.140065283	0.010517687
FALSE	66	rs6476434	DCAF12	ENSG00000198876	FALSE	96731	FALSE	0.734	0.135	8	0.027033797	0.006059957
FALSE	66	rs6476434	UBAP1	ENSG00000165006	FALSE	189349	FALSE	0.879	0.247	8	0.020664599	0.013270808
FALSE	66	rs6476434	PRSS3	ENSG00000010438	FALSE	190425	FALSE	0.166	-0.182	8	0.116139814	0.008896104
FALSE	66	rs6476434	KIF24	ENSG00000186638	FALSE	262724	FALSE	0.662	0.091	8	0.009847893	0.010469683
FALSE	66	rs6476434	NUDT2	ENSG00000164978	FALSE	339850	FALSE	0.888	0.258	8	0.008843789	0.015394062
FALSE	66	rs6476434	ANKRD18B	ENSG00000230453	FALSE	380808	FALSE	0.169	-0.180	8	0.010124623	0.005903999
TRUE	67	rs878321	ITGA8	ENSG00000077943	TRUE	699	TRUE	0.999	0.974	3	0.73591435	0.1793589
FALSE	67	rs878321	FAM171A1	ENSG00000148468	FALSE	141966	FALSE	0.533	0.020	3	0.137828052	0.11864184
FALSE	67	rs878321	MINDY3	ENSG00000148481	FALSE	264915	FALSE	0.852	0.222	3	0.017701559	
TRUE	68	rs10748818	GBF1	ENSG00000107862	TRUE	0	TRUE	0.975	0.446	18	0.374604672	0.289151733
FALSE	68	rs10748818	PITX3	ENSG00000107859	FALSE	10272	FALSE	0.518	0.012	18	0.06811776	0.123372275
FALSE	68	rs10748818	ELOVL3	ENSG00000119915	FALSE	22195	FALSE	0.036	-0.366	18	0.023143793	0.008568173
FALSE	68	rs10748818	NOLC1	ENSG00000166197	FALSE	87913	FALSE	0.516	0.011	18	0.016561702	0.002376351
FALSE	68	rs10748818	PPRC1	ENSG00000148840	FALSE	101451	FALSE	0.854	0.223	18	0.020344947	0.002376351
FALSE	68	rs10748818	LDB1	ENSG00000198728	FALSE	131416	FALSE	0.716	0.123	18	0.011606472	0.002372642
FALSE	68	rs10748818	NFKB2	ENSG00000077150	FALSE	142326	FALSE	0.962	0.391	18	0.075345635	0.05776466
FALSE	68	rs10748818	PSD	ENSG00000059915	FALSE	150833	FALSE	0.931	0.318	18	0.050618321	0.244885789

FALSE	68	rs10748818	FBXL15	ENSG00000107872	FALSE	167405	FALSE	0.700	0.112		18	0.019742232	0.110877162
FALSE	68	rs10748818	CUEDC2	ENSG00000107874	FALSE	171461	FALSE	0.534	0.021		18	0.016211569	0.069295513
FALSE	68	rs10748818	HPS6	ENSG00000166189	FALSE	183748	FALSE	0.904	0.278		18	0.011254336	0.002356406
FALSE	68	rs10748818	ARMH3	ENSG00000120029	FALSE	195591	FALSE	0.773	0.162		18	0.014757886	
FALSE	68	rs10748818	C10orf95	ENSG00000120055	FALSE	198053	FALSE	0.263	-0.124		18	0.016139852	0.004200493
FALSE	68	rs10748818	MFSD13A	ENSG00000138111	FALSE	209611	FALSE	0.465	-0.016		18	0.013306722	
FALSE	68	rs10748818	ACTR1A	ENSG00000138107	FALSE	210097	FALSE	0.836	0.208		18	0.010283733	0.002466304
FALSE	68	rs10748818	SUFU	ENSG00000107882	FALSE	252188	FALSE	0.859	0.229		18	0.00829654	0.002445627
FALSE	68	rs10748818	TRIM8	ENSG00000171206	FALSE	390719	FALSE	0.965	0.402		18	0.006651562	0.002336088
FALSE	68	rs10748818	ARL3	ENSG00000138175	FALSE	421947	FALSE	0.496	0.001		18	0.00805008	0.002506033
TRUE	69	rs72840788	BAG3	ENSG00000151929	TRUE	0	TRUE	0.994	0.644	0.150556488	7	0.705632985	0.406988442
FALSE	69	rs72840788	INPP5F	ENSG00000198825	FALSE	60830	FALSE	0.774	0.163		7	0.028153244	0.435154991
FALSE	69	rs72840788	TIAL1	ENSG00000151923	FALSE	68183	FALSE	0.934	0.323		7	0.018792847	0.070647022
FALSE	69	rs72840788	RGS10	ENSG00000148908	FALSE	122493	FALSE	0.954	0.369		7	0.025689175	0.041255572
FALSE	69	rs72840788	MCMBP	ENSG00000197771	FALSE	164192	FALSE	0.568	0.038		7	0.02147988	0.003647261
FALSE	69	rs72840788	GRK5	ENSG00000198873	FALSE	205467	FALSE	0.520	0.014		7	0.011608703	0.004322463
FALSE	69	rs72840788	SEC23IP	ENSG00000107651	FALSE	227495	FALSE	0.808	0.187		7	0.034197867	0.006591534
FALSE	70	rs117896735	INPP5F	ENSG00000198825	TRUE	0	FALSE	0.774	0.163		6	0.028153244	0.435154991
FALSE	70	rs117896735	MCMBP	ENSG00000197771	FALSE	52589	FALSE	0.568	0.038		6	0.02147988	0.003647261
FALSE	70	rs117896735	BAG3	ENSG00000151929	FALSE	98996	TRUE	0.994	0.644		6	0.705632985	0.406988442
FALSE	70	rs117896735	SEC23IP	ENSG00000107651	FALSE	115892	FALSE	0.808	0.187		6	0.034197867	0.006591534
FALSE	70	rs117896735	TIAL1	ENSG00000151923	FALSE	179786	FALSE	0.934	0.323		6	0.018792847	0.070647022
FALSE	70	rs117896735	RGS10	ENSG00000148908	FALSE	234096	FALSE	0.954	0.369		6	0.025689175	0.041255572
FALSE	71	rs4910149	RNF141	ENSG00000110315	TRUE	0	FALSE	0.970	0.424		9		0.6748906
FALSE	71	rs4910149	AMPD3	ENSG00000133805	FALSE	12252	FALSE	0.901	0.274		9		0.03733752
FALSE	71	rs4910149	LYVE1	ENSG00000133800	FALSE	37135	TRUE	0.997	0.801		9		0.020230565
FALSE	71	rs4910149	IRAG1	ENSG00000072952	FALSE	53260	FALSE	0.993	0.618		9		
FALSE	71	rs4910149	ADM	ENSG00000148926	FALSE	212434	FALSE	0.049	-0.329		9		0.007812558
FALSE	71	rs4910149	SBF2	ENSG00000133812	FALSE	214954	FALSE	0.873	0.241		9		0.013688249
FALSE	71	rs4910149	CTR9	ENSG00000198730	FALSE	231415	FALSE	0.730	0.132		9		0.007812558
FALSE	71	rs4910149	EIF4G2	ENSG00000110321	FALSE	277219	FALSE	0.851	0.221		9		0.011781662
FALSE	71	rs4910149	ZBED5	ENSG00000236287	FALSE	292243	FALSE	0.320	-0.092		9		0.007745196
TRUE	72	rs12283611	DLG2	ENSG00000150672	TRUE	0	TRUE	0.999	1.158		1	0.471075267	0.67603385
FALSE	73	rs2156675	IGSF9B	ENSG00000080854	TRUE	15871	FALSE	0.860	0.229		5	0.796442747	0.763293264
FALSE	73	rs2156675	SPATA19	ENSG00000166118	FALSE	35029	FALSE	0.235	-0.140		5	0.090994748	0.03942276
FALSE	73	rs2156675	JAM3	ENSG00000166086	FALSE	188504	TRUE	0.905	0.279		5	0.038769219	0.138102256

FALSE	73	rs2156675	NCAPD3	ENSG00000151503	FALSE	269546	FALSE	0.644	0.080		5	0.00769546	0.006806889
FALSE	73	rs2156675	VPS26B	ENSG00000151502	FALSE	344103	FALSE	0.652	0.085		5	0.006830698	0.006806889
FALSE	74	rs140427697	BICD1	ENSG00000151746	TRUE	0	TRUE	0.970	0.422		2		
FALSE	74	rs140427697	FGD4	ENSG00000139132	FALSE	60819	FALSE	0.704	0.115		2		
FALSE	75	rs814228	SLC2A13	ENSG00000151229	TRUE	0	FALSE	0.996	0.729		3	0.044013392	0.427145786
FALSE	75	rs814228	C12orf40	ENSG00000180116	FALSE	80564	FALSE	0.110	-0.231		3	0.022593128	0.004209084
FALSE	75	rs814228	LRRK2	ENSG00000188906	FALSE	207880	TRUE	1.000	2.242		3	0.837437928	0.503189471
FALSE	76	rs28370650	SLC2A13	ENSG00000151229	TRUE	0	FALSE	0.996	0.729		3	0.044013392	0.427145786
FALSE	76	rs28370650	C12orf40	ENSG00000180116	FALSE	97452	FALSE	0.110	-0.231		3	0.022593128	0.004209084
FALSE	76	rs28370650	LRRK2	ENSG00000188906	FALSE	190992	TRUE	1.000	2.242		3	0.837437928	0.503189471
FALSE	77	rs7134524	SLC2A13	ENSG00000151229	TRUE	0	FALSE	0.996	0.729		4	0.044013392	0.427145786
FALSE	77	rs7134524	C12orf40	ENSG00000180116	FALSE	142864	FALSE	0.110	-0.231		4	0.022593128	0.004209084
FALSE	77	rs7134524	LRRK2	ENSG00000188906	FALSE	145580	TRUE	1.000	2.242		4	0.837437928	0.503189471
FALSE	77	rs7134524	MUC19	ENSG00000205592	FALSE	342231	FALSE	0.335	-0.085		4		0.043289674
FALSE	78	rs144296031	SLC2A13	ENSG00000151229	TRUE	0	FALSE	0.996	0.729		4	0.044013392	0.427145786
FALSE	78	rs144296031	LRRK2	ENSG00000188906	FALSE	139112	TRUE	1.000	2.242		4	0.837437928	0.503189471
FALSE	78	rs144296031	C12orf40	ENSG00000180116	FALSE	149332	FALSE	0.110	-0.231		4	0.022593128	0.004209084
FALSE	78	rs144296031	MUC19	ENSG00000205592	FALSE	335763	FALSE	0.335	-0.085		4		0.043289674
TRUE	79	rs141336855	LRRK2	ENSG00000188906	TRUE	28721	TRUE	1.000	2.242		4	0.837437928	0.503189471
FALSE	79	rs141336855	SLC2A13	ENSG00000151229	FALSE	61934	FALSE	0.996	0.729		4	0.044013392	0.427145786
FALSE	79	rs141336855	MUC19	ENSG00000205592	FALSE	225372	FALSE	0.335	-0.085		4		0.043289674
FALSE	79	rs141336855	C12orf40	ENSG00000180116	FALSE	259723	FALSE	0.110	-0.231		4	0.022593128	0.004209084
TRUE	80	rs4272849	LRRK2	ENSG00000188906	TRUE	0	TRUE	1.000	2.242		4	0.837437928	0.503189471
FALSE	80	rs4272849	MUC19	ENSG00000205592	FALSE	123977	FALSE	0.335	-0.085		4		0.043289674
FALSE	80	rs4272849	SLC2A13	ENSG00000151229	FALSE	163329	FALSE	0.996	0.729		4	0.044013392	0.427145786
FALSE	80	rs4272849	CNTN1	ENSG00000018236	FALSE	423021	FALSE	0.941	0.338		4	0.023282932	0.00663422
TRUE	81	rs17442721	LRRK2	ENSG00000188906	TRUE	0	TRUE	1.000	2.242		4	0.837437928	0.503189471
FALSE	81	rs17442721	MUC19	ENSG00000205592	FALSE	68823	FALSE	0.335	-0.085		4		0.043289674
FALSE	81	rs17442721	SLC2A13	ENSG00000151229	FALSE	218483	FALSE	0.996	0.729		4	0.044013392	0.427145786
FALSE	81	rs17442721	C12orf40	ENSG00000180116	FALSE	416272	FALSE	0.110	-0.231		4	0.022593128	0.004209084
FALSE	82	rs11179174	CNTN1	ENSG00000018236	TRUE	0	TRUE	0.941	0.338		3	0.023282932	0.00663422
FALSE	82	rs11179174	PDZRN4	ENSG00000165966	FALSE	278586	FALSE	0.555	0.032		3		0.005831447
FALSE	82	rs11179174	MUC19	ENSG00000205592	FALSE	338902	FALSE	0.335	-0.085		3		0.043289674
FALSE	83	rs7134559	SCAF11	ENSG00000139218	TRUE	18927	FALSE	0.876	0.243	0.0072333	4	0.578471959	0.858275227
FALSE	83	rs7134559	ARID2	ENSG00000189079	FALSE	103007	TRUE	0.990	0.570		4	0.240334436	0.038210444
FALSE	83	rs7134559	SLC38A1	ENSG00000111371	FALSE	172016	FALSE	0.931	0.317		4	0.02433023	0.045750456

FALSE	83	rs7134559	SLC38A2	ENSG00000134294	FALSE	347141	FALSE	0.914	0.293	4	0.018760316	0.023735423
FALSE	84	rs10847864	HIP1R	ENSG00000130787	TRUE	0	FALSE	0.980	0.468	12	0.714345002	0.427253183
FALSE	84	rs10847864	CCDC62	ENSG00000130783	FALSE	14523	FALSE	0.666	0.094	12	0.027209221	0.284743978
FALSE	84	rs10847864	VPS37B	ENSG00000139722	FALSE	23279	FALSE	0.876	0.244	12	0.028666493	0.092021326
FALSE	84	rs10847864	DENR	ENSG00000139726	FALSE	70987	FALSE	0.535	0.021	12	0.039890288	0.031569122
FALSE	84	rs10847864	ABCB9	ENSG00000150967	FALSE	78900	TRUE	0.985	0.507	12	0.018448261	0.007813036
FALSE	84	rs10847864	HCAR1	ENSG00000196917	FALSE	111207	FALSE	0.594	0.053	12	0.018775935	0.005644796
FALSE	84	rs10847864	HCAR3	ENSG00000255398	FALSE	125240	FALSE	0.729	0.131	12	0.015605877	0.005135541
FALSE	84	rs10847864	OGFOD2	ENSG00000111325	FALSE	132529	FALSE	0.682	0.102	12	0.013262073	0.003794811
FALSE	84	rs10847864	ARL6IP4	ENSG00000182196	FALSE	138009	FALSE	0.886	0.255	12	0.016091415	0.006827775
FALSE	84	rs10847864	HCAR2	ENSG00000182782	FALSE	138694	FALSE	0.764	0.156	12	0.015041922	0.006827775
FALSE	84	rs10847864	PITPNM2	ENSG00000090975	FALSE	141429	FALSE	0.902	0.276	12	0.012056412	0.004616098
FALSE	84	rs10847864	KNTC1	ENSG00000184445	FALSE	215655	FALSE	0.931	0.319	12	0.010317361	0.003762091
TRUE	85	rs11610045	FBRSL1	ENSG00000112787	TRUE	2672	TRUE	0.982	0.482	9	0.563869178	0.664090012
FALSE	85	rs11610045	LRCOL1	ENSG00000204583	FALSE	116271	FALSE	NA	NA	9	0.019326713	
FALSE	85	rs11610045	P2RX2	ENSG00000187848	FALSE	131897	FALSE	0.276	-0.117	9	0.017774628	0.00842256
FALSE	85	rs11610045	POLE	ENSG00000177084	FALSE	136874	FALSE	0.352	-0.076	9	0.021472555	0.009147789
FALSE	85	rs11610045	GALNT9	ENSG00000182870	FALSE	157530	FALSE	0.053	-0.315	9	0.017632872	0.012405882
FALSE	85	rs11610045	PXMP2	ENSG00000176894	FALSE	200708	FALSE	0.455	-0.022	9	0.015994957	0.008453817
FALSE	85	rs11610045	PGAM5	ENSG00000247077	FALSE	223940	FALSE	0.770	0.160	9	0.016947629	0.009147789
FALSE	85	rs11610045	ANKLE2	ENSG00000176915	FALSE	238624	FALSE	0.788	0.173	9	0.011441734	0.012299673
FALSE	85	rs11610045	GOLGA3	ENSG00000090615	FALSE	282023	FALSE	0.803	0.183	9	0.014168362	0.018641061
TRUE	86	rs9535211	CAB39L	ENSG00000102547	TRUE	0	TRUE	0.979	0.465	13	0.695689213	0.657187987
FALSE	86	rs9535211	SETDB2	ENSG00000136169	FALSE	26204	FALSE	0.677	0.099	13	0.097736532	0.023720048
FALSE	86	rs9535211	PHF11	ENSG00000136147	FALSE	77540	FALSE	0.391	-0.056	13	0.029592143	0.023068172
FALSE	86	rs9535211	RCBTB1	ENSG00000136144	FALSE	113876	FALSE	0.531	0.019	13	0.017433174	0.033008804
FALSE	86	rs9535211	CDADC1	ENSG00000102543	FALSE	124585	FALSE	0.407	-0.047	13	0.085236084	0.023914935
FALSE	86	rs9535211	MLNR	ENSG00000102539	FALSE	195693	FALSE	0.075	-0.275	13	0.03146846	0.022461256
FALSE	86	rs9535211	FNDC3A	ENSG00000102531	FALSE	208291	FALSE	0.809	0.187	13	0.008313404	0.089137083
FALSE	86	rs9535211	ARL11	ENSG00000152213	FALSE	210437	FALSE	0.477	-0.009	13	0.01385225	0.022271098
FALSE	86	rs9535211	EBPL	ENSG00000123179	FALSE	242604	FALSE	0.789	0.173	13	0.012363875	0.034581864
FALSE	86	rs9535211	KPNA3	ENSG00000102753	FALSE	281250	FALSE	0.501	0.003	13	0.008314866	0.012999575
FALSE	86	rs9535211	SPRYD7	ENSG00000123178	FALSE	494632	FALSE	0.503	0.004	13		0.006654204
FALSE	86	rs9535211	TRIM13	ENSG00000204977	FALSE	577818	FALSE	0.566	0.037	13		0.006654204
FALSE	86	rs9535211	KCNRG	ENSG00000198553	FALSE	597184	FALSE	0.591	0.051	13		0.006654204
FALSE	87	rs6491345	MBNL2	ENSG00000139793	TRUE	0	FALSE	0.465	-0.016	3	0.811723411	0.911305893

FALSE	87	rs6491345	RAP2A	ENSG00000125249	FALSE	192122	TRUE	0.913	0.292		3	0.024666972	0.015911247
FALSE	87	rs6491345	OXGR1	ENSG00000165621	FALSE	247317	FALSE	0.109	-0.232		3	0.031996615	0.009821105
FALSE	88	rs8005136	MIPOL1	ENSG00000151338	TRUE	0	FALSE	0.820	0.195		4	0.313065671	0.6940375
FALSE	88	rs8005136	FOXA1	ENSG00000129514	FALSE	93999	TRUE	0.989	0.554	0.005522965	4	0.300961067	0.027362138
FALSE	88	rs8005136	TTC6	ENSG00000139865	FALSE	100076	FALSE	0.509	0.008		4	0.366866897	0.13273928
FALSE	88	rs8005136	SLC25A21	ENSG00000183032	FALSE	322947	FALSE	0.845	0.215		4	0.019106364	0.009997049
FALSE	89	rs11158026	WDHD1	ENSG00000198554	TRUE	8833	FALSE	0.771	0.161		8	0.102233134	0.201573785
FALSE	89	rs11158026	GCH1	ENSG00000131979	FALSE	27290	TRUE	1.000	1.422		8	0.735757828	0.487470235
FALSE	89	rs11158026	SOCS4	ENSG00000180008	FALSE	97114	FALSE	0.820	0.195		8	0.029433692	0.203171732
FALSE	89	rs11158026	MAPK1IP1L	ENSG00000168175	FALSE	121531	FALSE	0.663	0.092		8	0.01972873	0.046445955
FALSE	89	rs11158026	SAMD4A	ENSG00000020577	FALSE	136801	FALSE	0.898	0.271		8	0.014010593	0.006760953
FALSE	89	rs11158026	LGALS3	ENSG00000131981	FALSE	193994	FALSE	0.888	0.258		8	0.018788619	0.005871655
FALSE	89	rs11158026	DLGAP5	ENSG00000126787	FALSE	217996	FALSE	0.135	-0.206		8	0.010713015	0.003924357
FALSE	89	rs11158026	FBXO34	ENSG00000178974	FALSE	341228	FALSE	0.750	0.145		8	0.009311166	0.00962287
FALSE	90	rs3742785	PROX2	ENSG00000119608	TRUE	0	FALSE	0.475	-0.011		18	0.147128373	0.036342226
FALSE	90	rs3742785	YLPM1	ENSG00000119596	FALSE	7239	FALSE	0.896	0.268		18	0.06899935	0.007006717
FALSE	90	rs3742785	DLST	ENSG00000119689	FALSE	15217	FALSE	0.870	0.238		18	0.121547565	0.321164172
FALSE	90	rs3742785	RPS6KL1	ENSG00000198208	FALSE	37277	FALSE	0.866	0.235		18	0.072481573	0.44342186
FALSE	90	rs3742785	PGF	ENSG00000119630	FALSE	75160	TRUE	0.951	0.362		18	0.033400577	0.05625313
FALSE	90	rs3742785	FCF1	ENSG00000119616	FALSE	128054	FALSE	0.779	0.166		18	0.092209466	0.005949486
FALSE	90	rs3742785	EIF2B2	ENSG00000119718	FALSE	136247	FALSE	0.532	0.020		18	0.010173619	0.005926706
FALSE	90	rs3742785	MLH3	ENSG00000119684	FALSE	147095	FALSE	0.782	0.168		18	0.012337978	0.008230574
FALSE	90	rs3742785	AREL1	ENSG00000119682	FALSE	153557	FALSE	0.927	0.311		18	0.200561151	0.004028848
FALSE	90	rs3742785	ACYP1	ENSG00000119640	FALSE	186563	FALSE	0.706	0.116		18	0.012331012	0.004111246
FALSE	90	rs3742785	ZC2HC1C	ENSG00000119703	FALSE	197435	FALSE	0.439	-0.030		18	0.010296375	0.004011781
FALSE	90	rs3742785	NEK9	ENSG00000119638	FALSE	212679	FALSE	0.822	0.197		18	0.010613093	0.004483809
FALSE	90	rs3742785	LTBP2	ENSG00000119681	FALSE	254296	FALSE	0.605	0.059		18	0.012835749	0.00687448
FALSE	90	rs3742785	TMED10	ENSG00000170348	FALSE	264795	FALSE	0.922	0.304		18	0.006303752	0.004229757
FALSE	90	rs3742785	ISCA2	ENSG00000165898	FALSE	369568	FALSE	0.562	0.035		18	0.007765315	0.006922589
FALSE	90	rs3742785	NPC2	ENSG00000119655	FALSE	372497	FALSE	0.620	0.067		18	0.008913646	0.003977195
FALSE	90	rs3742785	SYNDIG1L	ENSG00000183379	FALSE	440464	FALSE	0.541	0.025		18	0.009052954	0.00390762
FALSE	90	rs3742785	VRTN	ENSG00000133980	FALSE	506666	FALSE	0.150	-0.194		18		0.00390762
TRUE	91	rs2008686	GPR65	ENSG00000140030	TRUE	714	TRUE	0.988	0.548		3	0.525071023	0.107523018
FALSE	91	rs2008686	GALC	ENSG00000054983	FALSE	21860	FALSE	0.983	0.485		3	0.432989745	0.78692148
FALSE	91	rs2008686	KCNK10	ENSG00000100433	FALSE	164578	FALSE	0.541	0.024		3	0.012127417	0.012964242
FALSE	92	rs28648524	TRPM7	ENSG00000092439	TRUE	0	FALSE	0.886	0.256		10		

FALSE	92	rs28648524	USP50	ENSG00000170236	FALSE	13839	FALSE	0.724	0.127		10		
FALSE	92	rs28648524	USP8	ENSG00000138592	FALSE	46126	TRUE	0.950	0.358		10		
FALSE	92	rs28648524	ENSG00000288645	ENSG00000288645	FALSE	88255	FALSE	NA	NA		10		
FALSE	92	rs28648524	SPPL2A	ENSG00000138600	FALSE	141719	FALSE	0.706	0.116		10		
FALSE	92	rs28648524	GABPB1	ENSG00000104064	FALSE	205139	FALSE	0.291	-0.108		10		
FALSE	92	rs28648524	HDC	ENSG00000140287	FALSE	294582	FALSE	0.014	-0.503		10		
FALSE	92	rs28648524	SLC27A2	ENSG00000140284	FALSE	324162	FALSE	0.071	-0.283		10		
FALSE	92	rs28648524	AP4E1	ENSG00000081014	FALSE	348125	FALSE	0.757	0.150		10		
FALSE	92	rs28648524	ATP8B4	ENSG00000104043	FALSE	377730	FALSE	0.115	-0.225		10		
TRUE	93	rs2251086	VPS13C	ENSG00000129003	TRUE	148601	TRUE	0.697	0.111		1	0.290622234	0.300101
TRUE	94	rs6497339	SYT17	ENSG00000103528	TRUE	0	TRUE	0.972	0.429		9	0.597931802	0.3564295
FALSE	94	rs6497339	CLEC19A	ENSG00000261210	FALSE	18605	FALSE	0.176	-0.175		9	0.248082608	0.09211205
FALSE	94	rs6497339	TMC5	ENSG00000103534	FALSE	143370	FALSE	0.257	-0.127		9	0.019452568	0.007878686
FALSE	94	rs6497339	ITPRIPL2	ENSG00000205730	FALSE	145497	FALSE	0.459	-0.020		9	0.030689018	0.007866387
FALSE	94	rs6497339	COQ7	ENSG00000167186	FALSE	187031	FALSE	0.625	0.070		9	0.012841116	0.007878686
FALSE	94	rs6497339	TMC7	ENSG00000170537	FALSE	203184	FALSE	0.267	-0.121		9	0.011776988	0.007829241
FALSE	94	rs6497339	GDE1	ENSG00000006007	FALSE	234567	FALSE	0.930	0.317		9	0.009415062	0.007878686
FALSE	94	rs6497339	CCP110	ENSG00000103540	FALSE	256685	FALSE	0.464	-0.016		9	0.007967195	0.008658815
FALSE	94	rs6497339	VPS35L	ENSG00000103544	FALSE	288114	FALSE	0.903	0.277		9	0.009156713	
FALSE	95	rs2904880	RABEP2	ENSG00000177548	TRUE	0	FALSE	0.931	0.319		16	0.035830848	0.118380104
FALSE	95	rs2904880	ATP2A1	ENSG00000196296	FALSE	1719	FALSE	0.724	0.128		16	0.101540952	0.02291168
FALSE	95	rs2904880	CD19	ENSG00000177455	FALSE	25780	FALSE	0.039	-0.356		16	0.157950499	0.164268673
FALSE	95	rs2904880	SH2B1	ENSG00000178188	FALSE	31973	FALSE	0.888	0.257	0.005741891	16	0.114641017	0.015974532
FALSE	95	rs2904880	NFATC2IP	ENSG00000176953	FALSE	44622	FALSE	0.424	-0.038		16	0.051133924	0.11224269
FALSE	95	rs2904880	TUFM	ENSG00000178952	FALSE	59837	FALSE	0.896	0.268		16	0.049792146	0.005498294
FALSE	95	rs2904880	SPNS1	ENSG00000169682	FALSE	68036	FALSE	0.956	0.374		16	0.125214439	0.271269072
FALSE	95	rs2904880	ATXN2L	ENSG00000168488	FALSE	68948	TRUE	0.957	0.376		16	0.082981806	0.006010971
FALSE	95	rs2904880	LAT	ENSG00000213658	FALSE	78641	FALSE	0.947	0.350		16	0.070617111	0.061292099
FALSE	95	rs2904880	NPIPB9	ENSG00000196993	FALSE	133321	FALSE	0.274	-0.118		16	0.020918203	0.005267276
FALSE	95	rs2904880	EIF3C	ENSG00000184110	FALSE	170458	FALSE	0.834	0.206		16	0.065099077	0.006369111
FALSE	95	rs2904880	NPIPB8	ENSG00000255524	FALSE	247441	FALSE	0.234	-0.140		16	0.014266401	0.00522186
FALSE	95	rs2904880	SULT1A1	ENSG00000196502	FALSE	291906	FALSE	0.560	0.034		16	0.014699611	0.015684962
FALSE	95	rs2904880	SULT1A2	ENSG00000197165	FALSE	309135	FALSE	0.402	-0.050		16	0.016082779	0.005623469
FALSE	95	rs2904880	SGF29	ENSG00000176476	FALSE	314395	FALSE	0.498	0.001		16	0.015472194	
FALSE	95	rs2904880	NUPR1	ENSG00000176046	FALSE	367177	FALSE	0.851	0.221		16	0.01128853	0.005184859
FALSE	96	rs11150601	SETD1A	ENSG00000099381	TRUE	0	FALSE	0.887	0.257		32	0.135776355	0.350914913

FALSE	96	rs11150601	ORAI3	ENSG00000175938	FALSE	1804	FALSE	0.515	0.011	32	0.03471793	0.055101549
FALSE	96	rs11150601	FBXL19	ENSG00000099364	FALSE	9482	FALSE	0.775	0.163	32	0.075749543	0.036947652
FALSE	96	rs11150601	HSD3B7	ENSG00000099377	FALSE	26942	FALSE	0.504	0.005	32	0.248689098	0.078473753
FALSE	96	rs11150601	STX1B	ENSG00000099365	FALSE	30991	FALSE	0.709	0.118	32	0.074239135	0.186529708
FALSE	96	rs11150601	CTF1	ENSG00000150281	FALSE	54718	FALSE	0.517	0.012	32	0.031416461	0.005053672
FALSE	96	rs11150601	BCL7C	ENSG00000099385	FALSE	63963	FALSE	0.898	0.270	32	0.014777434	0.018485471
FALSE	96	rs11150601	STX4	ENSG00000103496	FALSE	74624	FALSE	0.972	0.430	32	0.042957948	0.01471682
FALSE	96	rs11150601	ZNF668	ENSG00000167394	FALSE	102578	FALSE	0.908	0.284	32	0.013736193	0.005807979
FALSE	96	rs11150601	ZNF646	ENSG00000167395	FALSE	116157	FALSE	0.732	0.133	32	0.020363555	0.003246321
FALSE	96	rs11150601	PRSS53	ENSG00000151006	FALSE	125172	FALSE	0.726	0.129	32	0.018527238	0.003962712
FALSE	96	rs11150601	ENSG00000255439	ENSG00000255439	FALSE	125174	FALSE	NA	NA	32		
FALSE	96	rs11150601	VKORC1	ENSG00000167397	FALSE	132577	FALSE	0.459	-0.020	32	0.018909976	0.009924608
FALSE	96	rs11150601	BCKDK	ENSG00000103507	FALSE	147842	TRUE	0.979	0.461	32	0.014275574	0.003869311
FALSE	96	rs11150601	KAT8	ENSG00000103510	FALSE	156224	FALSE	0.806	0.186	32	0.011210583	0.008523742
FALSE	96	rs11150601	ZNF629	ENSG00000102870	FALSE	171060	FALSE	0.438	-0.031	32	0.008625051	0.003246321
FALSE	96	rs11150601	PRSS8	ENSG00000052344	FALSE	173168	FALSE	0.282	-0.113	32	0.013175357	0.003246321
FALSE	96	rs11150601	PRSS36	ENSG00000178226	FALSE	180661	FALSE	0.713	0.121	32	0.011659982	0.00713568
FALSE	96	rs11150601	RNF40	ENSG00000103549	FALSE	181958	FALSE	0.904	0.279	32	0.015167819	0.003246321
FALSE	96	rs11150601	CFAP119	ENSG00000196118	FALSE	196044	FALSE	0.360	-0.072	32	0.023489823	
FALSE	96	rs11150601	PHKG2	ENSG00000156873	FALSE	197089	FALSE	0.819	0.195	32	0.013259992	0.003246321
FALSE	96	rs11150601	TMEM265	ENSG00000281991	FALSE	213069	FALSE	NA	NA	32	0.007555171	
FALSE	96	rs11150601	ENSG00000282034	ENSG00000282034	FALSE	213096	FALSE	NA	NA	32		
FALSE	96	rs11150601	SRCAP	ENSG00000080603	FALSE	216856	FALSE	0.827	0.200	32	0.00927428	0.00321833
FALSE	96	rs11150601	FUS	ENSG00000089280	FALSE	221873	FALSE	0.960	0.386	32	0.010852786	0.005623915
FALSE	96	rs11150601	PYCARD	ENSG00000103490	FALSE	243221	FALSE	0.652	0.085	32	0.011692901	0.003246321
FALSE	96	rs11150601	TRIM72	ENSG00000177238	FALSE	255854	FALSE	0.203	-0.158	32	0.006790939	0.004838922
FALSE	96	rs11150601	PYDC1	ENSG00000169900	FALSE	257697	FALSE	0.534	0.021	32	0.005876017	0.003246321
FALSE	96	rs11150601	FBRS	ENSG00000156860	FALSE	287455	FALSE	0.966	0.402	32	0.0088139	0.00321833
FALSE	96	rs11150601	ITGAM	ENSG00000169896	FALSE	301702	FALSE	0.686	0.104	32	0.005609587	0.005186592
FALSE	96	rs11150601	PRR14	ENSG00000156858	FALSE	301852	FALSE	0.725	0.129	32	0.007066088	0.005584768
FALSE	96	rs11150601	ZNF689	ENSG00000156853	FALSE	334253	FALSE	0.369	-0.068	32	0.005648263	0.00321833
TRUE	97	rs6500328	NOD2	ENSG00000167207	TRUE	0	TRUE	0.995	0.691	4	0.486185653	0.281535168
FALSE	97	rs6500328	SNX20	ENSG00000167208	FALSE	26226	FALSE	0.504	0.005	4	0.29811148	0.069285831
FALSE	97	rs6500328	CYLD	ENSG00000083799	FALSE	34471	FALSE	0.985	0.509	4	0.149006131	0.601617108
FALSE	97	rs6500328	NKD1	ENSG00000140807	FALSE	58330	FALSE	0.868	0.236	4	0.032885614	0.005406701
TRUE	98	rs3104783	TOX3	ENSG00000103460	TRUE	9687	TRUE	0.998	0.843	1	0.678462803	0.75447317

FALSE	99	rs10221156	CHD9NB	ENSG00000277639	TRUE	104429	FALSE	NA	NA		2		
FALSE	99	rs10221156	CHD9	ENSG00000177200	FALSE	123730	TRUE	0.791	0.174		2	0.523112476	0.175312312
FALSE	100	rs9217	ZBTB4	ENSG00000174282	TRUE	0	FALSE	0.982	0.479		93	0.149201421	0.278820276
FALSE	100	rs9217	SLC35G6	ENSG00000259224	TRUE	0	FALSE	0.298	-0.104		93	0.113404263	0.042359862
FALSE	100	rs9217	POLR2A	ENSG00000181222	FALSE	1371	FALSE	0.987	0.536		93	0.101157788	0.214464949
FALSE	100	rs9217	CHRNB1	ENSG00000170175	FALSE	25285	FALSE	0.800	0.181	0.000520426	93	0.138442595	0.095377824
FALSE	100	rs9217	FGF11	ENSG00000161958	FALSE	38058	FALSE	0.512	0.009		93	0.021858698	0.054876243
FALSE	100	rs9217	TMEM102	ENSG00000181284	FALSE	45316	FALSE	0.421	-0.040		93	0.017945908	0.02602444
FALSE	100	rs9217	ENSG00000286007	ENSG00000286007	FALSE	46717	FALSE	NA	NA		93		
FALSE	100	rs9217	SPEM3	ENSG00000283439	FALSE	50175	FALSE	NA	NA		93	0.01244949	
FALSE	100	rs9217	SPEM2	ENSG00000184560	FALSE	55427	FALSE	0.325	-0.090		93	0.013814796	
FALSE	100	rs9217	SPEM1	ENSG00000181323	FALSE	61363	FALSE	0.342	-0.082		93	0.013434724	0.008275214
FALSE	100	rs9217	NLGN2	ENSG00000169992	FALSE	63135	FALSE	0.977	0.454		93	0.013454978	0.058993725
FALSE	100	rs9217	TNFSF12	ENSG00000239697	FALSE	65511	FALSE	0.706	0.116		93	0.018129212	0.004941253
FALSE	100	rs9217	TNFSF12-TNFSF13	ENSG00000248871	FALSE	66102	FALSE	NA	NA		93	0.014738704	0.004065662
FALSE	100	rs9217	TNFSF13	ENSG00000161955	FALSE	75295	FALSE	0.633	0.075	0.001230813	93	0.013703002	0.004254046
FALSE	100	rs9217	TMEM256	ENSG00000205544	FALSE	78898	FALSE	0.459	-0.020		93	0.011046568	0.004065662
FALSE	100	rs9217	TMEM256-PLSCR3	ENSG00000262481	FALSE	78898	FALSE	NA	NA		93	0.011046568	0.004065662
FALSE	100	rs9217	SENP3	ENSG00000161956	FALSE	78922	FALSE	0.656	0.088		93	0.010421047	0.004065662
FALSE	100	rs9217	SENP3-EIF4A1	ENSG00000277957	FALSE	80290	FALSE	NA	NA		93	0.010147225	
FALSE	100	rs9217	PLSCR3	ENSG00000187838	FALSE	88153	FALSE	NA	NA		93	0.009945007	
FALSE	100	rs9217	EIF4A1	ENSG00000161960	FALSE	89828	FALSE	0.554	0.031		93	0.009809481	0.008530742
FALSE	100	rs9217	TNK1	ENSG00000174292	FALSE	93221	FALSE	0.381	-0.062		93	0.010321579	0.004065662
FALSE	100	rs9217	CD68	ENSG00000129226	FALSE	96495	FALSE	0.936	0.328	0.010907015	93	0.010020993	0.00411567
FALSE	100	rs9217	MPDU1	ENSG00000129255	FALSE	100533	FALSE	0.915	0.293	0.018403574	93	0.009840625	0.00413175
FALSE	100	rs9217	SOX15	ENSG00000129194	FALSE	105182	FALSE	0.642	0.079		93	0.008538394	0.002296482
FALSE	100	rs9217	FXR2	ENSG00000129245	FALSE	108234	FALSE	0.797	0.178		93	0.00757795	0.002476726
FALSE	100	rs9217	TMEM95	ENSG00000182896	FALSE	125776	FALSE	0.196	-0.163		93	0.008133503	0.002296482
FALSE	100	rs9217	KCTD11	ENSG00000213859	FALSE	128051	FALSE	0.654	0.087		93	0.008731773	0.002296482
FALSE	100	rs9217	SHBG	ENSG00000129214	FALSE	130950	FALSE	0.834	0.206	0.002561463	93	0.008608602	0.002296482
FALSE	100	rs9217	ACAP1	ENSG00000072818	FALSE	131518	FALSE	0.528	0.017		93	0.007307973	0.002296482
FALSE	100	rs9217	SAT2	ENSG00000141504	FALSE	143238	FALSE	0.591	0.051		93	0.007841649	0.002320632
FALSE	100	rs9217	NEURL4	ENSG00000215041	FALSE	153602	FALSE	0.878	0.246		93	0.006353612	0.002296482
FALSE	100	rs9217	ATP1B2	ENSG00000129244	FALSE	163631	FALSE	0.611	0.062		93	0.007332663	0.002280768
FALSE	100	rs9217	ENSG00000261915	ENSG00000261915	FALSE	163821	FALSE	NA	NA		93		
FALSE	100	rs9217	GPS2	ENSG00000132522	FALSE	167431	FALSE	0.946	0.349		93	0.007753285	0.002280768

FALSE	100	rs9217	EIF5A	ENSG00000132507	FALSE	170532	FALSE	0.908	0.284	93	0.009518741	0.002280768
FALSE	100	rs9217	TP53	ENSG00000141510	FALSE	178783	FALSE	0.912	0.289	93	0.006367648	0.002288077
FALSE	100	rs9217	YBX2	ENSG00000006047	FALSE	188356	FALSE	0.243	-0.135	93	0.007318147	0.002280768
FALSE	100	rs9217	SLC2A4	ENSG00000181856	FALSE	194738	FALSE	0.514	0.010	93	0.006383865	0.002280768
FALSE	100	rs9217	WRAP53	ENSG00000141499	FALSE	203075	FALSE	0.856	0.226	93	0.006233466	0.002280768
FALSE	100	rs9217	CLDN7	ENSG00000181885	FALSE	219012	FALSE	0.817	0.193	93	0.007445913	0.002280768
FALSE	100	rs9217	ENSG00000262302	ENSG00000262302	FALSE	220906	FALSE	NA	NA	93		
FALSE	100	rs9217	EFNB3	ENSG00000108947	FALSE	222206	FALSE	0.399	-0.052	93	0.005322529	0.002280768
FALSE	100	rs9217	ELP5	ENSG00000170291	FALSE	223055	FALSE	0.414	-0.043	93	0.005805638	0.002280768
FALSE	100	rs9217	CTDNEP1	ENSG00000175826	FALSE	230504	FALSE	0.926	0.309	93	0.005906793	0.002280768
FALSE	100	rs9217	DNAH2	ENSG00000183914	FALSE	234748	FALSE	0.272	-0.119	93	0.005311316	0.002280768
FALSE	100	rs9217	ENSG00000262526	ENSG00000262526	FALSE	238360	FALSE	NA	NA	93		
FALSE	100	rs9217	GABARAP	ENSG00000170296	FALSE	240546	FALSE	0.628	0.072	93	0.004786433	0.002280768
FALSE	100	rs9217	PHF23	ENSG00000040633	FALSE	243273	FALSE	0.885	0.253	93	0.005285137	0.002280768
FALSE	100	rs9217	DVL2	ENSG00000004975	FALSE	248478	FALSE	0.770	0.160	93	0.005617979	0.002280768
FALSE	100	rs9217	ACADVL	ENSG00000072778	FALSE	257729	FALSE	0.746	0.143	93	0.005184823	0.002400213
FALSE	100	rs9217	DLG4	ENSG00000132535	FALSE	263154	TRUE	0.999	1.150	93	0.004775726	0.002280768
FALSE	100	rs9217	ASGR1	ENSG00000141505	FALSE	303431	FALSE	0.552	0.030	93	0.004728263	0.002261102
FALSE	100	rs9217	KDM6B	ENSG00000132510	FALSE	351221	FALSE	0.956	0.374	93	0.005168541	0.002261102
FALSE	100	rs9217	ASGR2	ENSG00000161944	FALSE	367295	FALSE	0.361	-0.072	93	0.004728263	0.002261102
FALSE	100	rs9217	TMEM88	ENSG00000167874	FALSE	372070	FALSE	0.376	-0.064	93	0.005016861	0.002261102
FALSE	100	rs9217	NAA38	ENSG00000183011	FALSE	373689	FALSE	0.814	0.191	93	0.004932389	
FALSE	100	rs9217	CYB5D1	ENSG00000182224	FALSE	374750	FALSE	0.715	0.122	93	0.008253474	0.002261102
FALSE	100	rs9217	CHD3	ENSG00000170004	FALSE	401800	FALSE	0.916	0.296	93	0.005101997	0.002236857
FALSE	100	rs9217	CLEC10A	ENSG00000132514	FALSE	402688	FALSE	0.205	-0.157	93	0.004729683	0.002261102
FALSE	100	rs9217	RNF227	ENSG00000179859	FALSE	430343	FALSE	NA	NA	93	0.0053004	
FALSE	100	rs9217	KCNAB3	ENSG00000170049	FALSE	438863	FALSE	0.056	-0.309	93	0.004927085	0.002236857
FALSE	100	rs9217	SLC16A11	ENSG00000174326	FALSE	438903	FALSE	0.502	0.004	93	0.004893685	0.002236857
FALSE	100	rs9217	SLC16A13	ENSG00000174327	FALSE	442878	FALSE	0.659	0.090	93	0.00536207	0.002236857
FALSE	100	rs9217	ENSG00000262730	ENSG00000262730	FALSE	442935	FALSE	NA	NA	93		
FALSE	100	rs9217	TRAPPC1	ENSG00000170043	FALSE	447349	FALSE	0.740	0.139	93	0.004715321	0.002236857
FALSE	100	rs9217	CNTROB	ENSG00000170037	FALSE	449105	FALSE	0.649	0.083	93	0.004715321	0.002236857
FALSE	100	rs9217	BCL6B	ENSG00000161940	FALSE	452705	FALSE	0.191	-0.165	93	0.004729683	0.002236857
FALSE	100	rs9217	C17orf49	ENSG00000258315	FALSE	465470	FALSE	0.607	0.060	93	0.004729683	0.002236857
FALSE	100	rs9217	RNASEK-C17orf49	ENSG00000161939	FALSE	465475	FALSE	NA	NA	93	0.004729683	0.002236857
FALSE	100	rs9217	RNASEK	ENSG00000219200	FALSE	468463	FALSE	0.878	0.245	93	0.004729683	0.002236857

FALSE	100	rs9217	ALOX12	ENSG00000108839	FALSE	472241	FALSE	0.558	0.033	93	0.004729683	0.002236857
FALSE	100	rs9217	GUCY2D	ENSG00000132518	FALSE	519619	FALSE	0.412	-0.044	93		0.002236857
FALSE	100	rs9217	ALOX15B	ENSG00000179593	FALSE	556038	FALSE	0.270	-0.120	93		0.002236857
FALSE	100	rs9217	ALOX12B	ENSG00000179477	FALSE	589640	FALSE	0.584	0.048	93		0.002236857
FALSE	100	rs9217	ALOXE3	ENSG00000179148	FALSE	612904	FALSE	0.518	0.012	93		0.002236857
FALSE	100	rs9217	HES7	ENSG00000179111	FALSE	637596	FALSE	0.545	0.026	93		0.002236857
FALSE	100	rs9217	PER1	ENSG00000179094	FALSE	657476	FALSE	0.996	0.717	93		0.002236857
FALSE	100	rs9217	ENSG00000263620	ENSG00000263620	FALSE	667820	FALSE	NA	NA	93		
FALSE	100	rs9217	VAMP2	ENSG00000220205	FALSE	676153	FALSE	0.963	0.396	93		0.003767025
FALSE	100	rs9217	TMEM107	ENSG00000179029	FALSE	689461	FALSE	0.602	0.057	93		0.002236857
FALSE	100	rs9217	BORCS6	ENSG00000196544	FALSE	705349	FALSE	0.458	-0.020	93		
FALSE	100	rs9217	AURKB	ENSG00000178999	FALSE	721737	FALSE	0.835	0.207	93		0.002236857
FALSE	100	rs9217	CTC1	ENSG00000178971	FALSE	741819	FALSE	0.972	0.427	93		0.002236857
FALSE	100	rs9217	PFAS	ENSG00000178921	FALSE	764622	FALSE	0.634	0.075	93		0.002236857
FALSE	100	rs9217	SLC25A35	ENSG00000125434	FALSE	804767	FALSE	0.596	0.053	93		0.002236857
FALSE	100	rs9217	RANGRF	ENSG00000108961	FALSE	805658	FALSE	0.675	0.098	93		0.002236857
FALSE	100	rs9217	ARHGEF15	ENSG00000198844	FALSE	827245	FALSE	0.429	-0.036	93		0.002236857
FALSE	100	rs9217	ODF4	ENSG00000184650	FALSE	856844	FALSE	0.347	-0.079	93		0.002236857
FALSE	100	rs9217	KRBA2	ENSG00000184619	FALSE	873906	FALSE	0.460	-0.019	93		0.002236857
FALSE	100	rs9217	ENSG00000263809	ENSG00000263809	FALSE	885642	FALSE	NA	NA	93		
FALSE	100	rs9217	RPL26	ENSG00000161970	FALSE	894520	FALSE	0.234	-0.140	93		0.002236857
FALSE	100	rs9217	RNF222	ENSG00000189051	FALSE	907706	FALSE	0.455	-0.022	93		0.002236857
FALSE	100	rs9217	NDEL1	ENSG00000166579	FALSE	930135	FALSE	0.994	0.666	93		0.002260914
TRUE	101	rs178654	NCOR1	ENSG00000141027	TRUE	0	TRUE	0.977	0.455	15		
FALSE	101	rs178654	TTC19	ENSG00000011295	FALSE	76488	FALSE	0.785	0.170	15		
FALSE	101	rs178654	PIGL	ENSG00000108474	FALSE	95688	FALSE	0.906	0.282	15		
FALSE	101	rs178654	ZSWIM7	ENSG00000214941	FALSE	121786	FALSE	0.595	0.053	15		
FALSE	101	rs178654	ADORA2B	ENSG00000170425	FALSE	145757	FALSE	0.549	0.029	15		
FALSE	101	rs178654	CENPV	ENSG00000166582	FALSE	221031	FALSE	0.931	0.318	15		
FALSE	101	rs178654	UBB	ENSG00000170315	FALSE	259295	FALSE	0.145	-0.198	15		
FALSE	101	rs178654	TRPV2	ENSG00000187688	FALSE	294068	FALSE	0.970	0.424	15		
FALSE	101	rs178654	LRRC75A	ENSG00000181350	FALSE	320074	FALSE	0.546	0.027	15		
FALSE	101	rs178654	TBC1D26	ENSG00000214946	FALSE	375341	FALSE	0.246	-0.133	15		
FALSE	101	rs178654	ZNF286A-TBC1D26	ENSG00000255104	FALSE	375563	FALSE	0.212	-0.153	15		
FALSE	101	rs178654	ZNF286A	ENSG00000187607	FALSE	400716	FALSE	0.419	-0.041	15		
FALSE	101	rs178654	ZNF287	ENSG00000141040	FALSE	425451	FALSE	0.716	0.123	15		

FALSE	101	rs178654	TRIM16	ENSG00000221926	FALSE	437192	FALSE	0.542	0.025		15		
FALSE	101	rs178654	ZNF624	ENSG00000197566	FALSE	499231	FALSE	0.139	-0.203		15		
FALSE	102	rs12944773	HNF1B	ENSG00000275410	TRUE	31782	TRUE	NA	NA		9		
FALSE	102	rs12944773	DDX52	ENSG00000278053	FALSE	133357	TRUE	NA	NA		9		
FALSE	102	rs12944773	TBC1D3K	ENSG00000273513	FALSE	147128	TRUE	NA	NA		9		
FALSE	102	rs12944773	TBC1D3F	ENSG00000185128	FALSE	147139	FALSE	0.297	-0.105		9		
FALSE	102	rs12944773	SYNRG	ENSG00000275066	FALSE	167312	TRUE	NA	NA		9		
FALSE	102	rs12944773	TBC1D3	ENSG00000197681	FALSE	200879	FALSE	0.350	-0.077		9		
FALSE	102	rs12944773	TBC1D3L	ENSG00000274512	FALSE	200879	TRUE	NA	NA		9		
FALSE	102	rs12944773	DUSP14	ENSG00000276023	FALSE	263229	TRUE	NA	NA		9		
FALSE	102	rs12944773	TADA2A	ENSG00000276234	FALSE	297002	TRUE	NA	NA		9		
FALSE	103	rs665268	HSD17B1	ENSG00000108786	TRUE	2563	FALSE	0.915	0.293		32	0.084254035	0.014926337
FALSE	103	rs665268	NAGLU	ENSG00000108784	FALSE	5910	FALSE	0.474	-0.011		32	0.034469985	0.014787432
FALSE	103	rs665268	COASY	ENSG00000068120	FALSE	11108	FALSE	0.575	0.042		32	0.045109152	0.050308615
FALSE	103	rs665268	MLX	ENSG00000108788	FALSE	16713	FALSE	0.760	0.153	0.461546728	32	0.207828738	0.30189218
FALSE	103	rs665268	PSMC3IP	ENSG00000131470	FALSE	21951	FALSE	0.330	-0.088		32	0.084937753	0.05171773
FALSE	103	rs665268	ATP6V0A1	ENSG00000033627	FALSE	27777	TRUE	0.994	0.645		32	0.015723979	0.010221091
FALSE	103	rs665268	RETREG3	ENSG00000141699	FALSE	29154	FALSE	0.775	0.164		32	0.038277682	
FALSE	103	rs665268	TUBG1	ENSG00000131462	FALSE	59282	FALSE	0.983	0.487		32	0.019588723	0.0232633
FALSE	103	rs665268	TUBG2	ENSG00000037042	FALSE	108925	FALSE	0.965	0.400		32	0.109223277	0.12409547
FALSE	103	rs665268	PLEKHH3	ENSG00000068137	FALSE	117555	FALSE	0.715	0.122		32	0.040968332	0.039800504
FALSE	103	rs665268	CAVIN1	ENSG00000177469	FALSE	127103	FALSE	0.732	0.134		32	0.03006851	
FALSE	103	rs665268	CCR10	ENSG00000184451	FALSE	128530	FALSE	0.025	-0.419		32	0.01586892	0.006077828
FALSE	103	rs665268	CNTNAP1	ENSG00000108797	FALSE	132172	FALSE	0.394	-0.055		32	0.017302481	0.016720138
FALSE	103	rs665268	EZH1	ENSG00000108799	FALSE	149916	FALSE	0.818	0.194		32	0.014177707	0.003274884
FALSE	103	rs665268	STAT3	ENSG00000168610	FALSE	161819	FALSE	0.816	0.193		32	0.009319049	0.0089152
FALSE	103	rs665268	RAMP2	ENSG00000131477	FALSE	208088	FALSE	0.059	-0.301		32	0.011097446	0.003036005
FALSE	103	rs665268	VPS25	ENSG00000131475	FALSE	223090	FALSE	0.821	0.196		32	0.009243281	0.003036005
FALSE	103	rs665268	WNK4	ENSG00000126562	FALSE	230251	FALSE	0.801	0.181		32	0.013229414	0.003036005
FALSE	103	rs665268	STAT5A	ENSG00000126561	FALSE	238416	FALSE	0.122	-0.218		32	0.00787919	0.003009828
FALSE	103	rs665268	COA3	ENSG00000183978	FALSE	244788	FALSE	0.441	-0.029		32	0.008862958	0.003036005
FALSE	103	rs665268	CNTD1	ENSG00000176563	FALSE	248441	FALSE	0.563	0.035		32	0.008810528	0.003036005
FALSE	103	rs665268	BECN1	ENSG00000126581	FALSE	259775	FALSE	0.980	0.468		32	0.011559264	0.003210108
FALSE	103	rs665268	STAT5B	ENSG00000173757	FALSE	261726	FALSE	0.254	-0.128		32	0.00682073	0.005230431
FALSE	103	rs665268	PSME3	ENSG00000131467	FALSE	274025	FALSE	0.607	0.060		32	0.007884682	0.003036005
FALSE	103	rs665268	AOC2	ENSG00000131480	FALSE	294220	FALSE	0.666	0.093		32	0.009019766	0.003036005

FALSE	103	rs665268	AOC3	ENSG00000131471	FALSE	300824	FALSE	0.570	0.039		32	0.011526043	0.003036005
FALSE	103	rs665268	G6PC1	ENSG00000131482	FALSE	350437	FALSE	0.148	-0.195		32	0.007244643	
FALSE	103	rs665268	GHDC	ENSG00000167925	FALSE	355846	FALSE	0.678	0.100		32	0.0064964	0.003009828
FALSE	103	rs665268	HCRT	ENSG00000161610	FALSE	364907	FALSE	0.385	-0.059		32	0.006851023	0.003009828
FALSE	103	rs665268	KCNH4	ENSG00000089558	FALSE	369217	FALSE	0.374	-0.066		32	0.006979401	0.003009828
FALSE	103	rs665268	RAB5C	ENSG00000108774	FALSE	395315	FALSE	0.941	0.338		32	0.008147322	0.002977554
FALSE	103	rs665268	ENSG00000267261	ENSG00000267261	FALSE	395443	FALSE	NA	NA		32		
FALSE	104	rs62053943	LINC02210-CRHR1	ENSG00000263715	TRUE	0	FALSE	NA	NA		6	0.737684869	
FALSE	104	rs62053943	CRHR1	ENSG00000120088	FALSE	117440	FALSE	0.993	0.618		6	0.350029378	0.051738397
FALSE	104	rs62053943	PLEKHM1	ENSG00000225190	FALSE	176079	FALSE	0.913	0.292		6	0.019346539	0.006766499
FALSE	104	rs62053943	SPPL2C	ENSG00000185294	FALSE	178044	FALSE	0.225	-0.145		6	0.587292492	0.101902905
FALSE	104	rs62053943	MAPT	ENSG00000186868	FALSE	227690	TRUE	0.998	0.890		6	0.06379012	0.540917437
FALSE	104	rs62053943	ARHGAP27	ENSG00000159314	FALSE	232416	FALSE	0.407	-0.047		6	0.014042274	0.004414258
FALSE	105	rs35941271	NSF	ENSG00000073969	TRUE	0	FALSE	0.992	0.603		8	0.526476576	0.055906609
FALSE	105	rs35941271	WNT3	ENSG00000108379	FALSE	10878	TRUE	1.000	1.597		8	0.680840211	0.315430112
FALSE	105	rs35941271	WNT9B	ENSG00000158955	FALSE	81573	FALSE	0.461	-0.018		8	0.179333608	0.021158912
FALSE	105	rs35941271	GOSR2	ENSG00000108433	FALSE	171447	FALSE	0.904	0.279		8	0.016136989	0.003256274
FALSE	105	rs35941271	ENSG00000262633	ENSG00000262633	FALSE	171505	FALSE	NA	NA		8		
FALSE	105	rs35941271	ARL17A	ENSG00000185829	FALSE	171933	FALSE	0.324	-0.091		8	0.069793883	0.008354106
FALSE	105	rs35941271	LRRC37A2	ENSG00000238083	FALSE	194718	FALSE	0.461	-0.018		8	0.056328559	0.004516927
FALSE	105	rs35941271	RPRML	ENSG00000179673	FALSE	226528	FALSE	0.608	0.060		8	0.019036692	0.003278557
FALSE	106	rs10208	NSF	ENSG00000073969	TRUE	0	FALSE	0.992	0.603		8	0.526476576	0.055906609
FALSE	106	rs10208	WNT3	ENSG00000108379	FALSE	10783	TRUE	1.000	1.597		8	0.680840211	0.315430112
FALSE	106	rs10208	WNT9B	ENSG00000158955	FALSE	81478	FALSE	0.461	-0.018		8	0.179333608	0.021158912
FALSE	106	rs10208	GOSR2	ENSG00000108433	FALSE	171352	FALSE	0.904	0.279		8	0.016136989	0.003256274
FALSE	106	rs10208	ENSG00000262633	ENSG00000262633	FALSE	171410	FALSE	NA	NA		8		
FALSE	106	rs10208	ARL17A	ENSG00000185829	FALSE	172028	FALSE	0.324	-0.091		8	0.069793883	0.008354106
FALSE	106	rs10208	LRRC37A2	ENSG00000238083	FALSE	194813	FALSE	0.461	-0.018		8	0.056328559	0.004516927
FALSE	106	rs10208	RPRML	ENSG00000179673	FALSE	226433	FALSE	0.608	0.060		8	0.019036692	0.003278557
FALSE	107	rs12951057	NSF	ENSG00000073969	TRUE	0	FALSE	0.992	0.603		8	0.526476576	0.055906609
FALSE	107	rs12951057	WNT3	ENSG00000108379	FALSE	8548	TRUE	1.000	1.597		8	0.680840211	0.315430112
FALSE	107	rs12951057	WNT9B	ENSG00000158955	FALSE	79243	FALSE	0.461	-0.018		8	0.179333608	0.021158912
FALSE	107	rs12951057	GOSR2	ENSG00000108433	FALSE	169117	FALSE	0.904	0.279		8	0.016136989	0.003256274
FALSE	107	rs12951057	ENSG00000262633	ENSG00000262633	FALSE	169175	FALSE	NA	NA		8		
FALSE	107	rs12951057	ARL17A	ENSG00000185829	FALSE	174263	FALSE	0.324	-0.091		8	0.069793883	0.008354106
FALSE	107	rs12951057	LRRC37A2	ENSG00000238083	FALSE	197048	FALSE	0.461	-0.018		8	0.056328559	0.004516927

FALSE	107	rs12951057	RPRML	ENSG00000179673	FALSE	224198	FALSE	0.608	0.060	8	0.019036692	0.003278557
FALSE	108	rs11652924	NSF	ENSG00000073969	TRUE	1892	FALSE	0.992	0.603	8	0.526476576	0.055906609
FALSE	108	rs11652924	WNT3	ENSG00000108379	FALSE	2935	TRUE	1.000	1.597	8	0.680840211	0.315430112
FALSE	108	rs11652924	WNT9B	ENSG00000158955	FALSE	73630	FALSE	0.461	-0.018	8	0.179333608	0.021158912
FALSE	108	rs11652924	GOSR2	ENSG00000108433	FALSE	163504	FALSE	0.904	0.279	8	0.016136989	0.003256274
FALSE	108	rs11652924	ENSG00000262633	ENSG00000262633	FALSE	163562	FALSE	NA	NA	8		
FALSE	108	rs11652924	ARL17A	ENSG00000185829	FALSE	179876	FALSE	0.324	-0.091	8	0.069793883	0.008354106
FALSE	108	rs11652924	LRRC37A2	ENSG00000238083	FALSE	202661	FALSE	0.461	-0.018	8	0.056328559	0.004516927
FALSE	108	rs11652924	RPRML	ENSG00000179673	FALSE	218585	FALSE	0.608	0.060	8	0.019036692	0.003278557
FALSE	109	rs112857769	INTS2	ENSG00000108506	TRUE	0	FALSE	0.913	0.292	6	0.488799821	0.13165429
FALSE	109	rs112857769	MED13	ENSG00000108510	FALSE	16534	TRUE	0.962	0.391	6	0.293093661	0.03503913
FALSE	109	rs112857769	BRIP1	ENSG00000136492	FALSE	62512	FALSE	0.876	0.244	6	0.172706393	0.5010731
FALSE	109	rs112857769	NACA2	ENSG00000253506	FALSE	334852	FALSE	0.415	-0.042	6	0.011206383	0.007866387
FALSE	109	rs112857769	RP11-51L5.7	ENSG00000270033	FALSE	346416	FALSE	NA	NA	6		
FALSE	109	rs112857769	EFCAB3	ENSG00000172421	FALSE	417870	FALSE	0.244	-0.135	6		0.007675922
FALSE	110	rs8071516	PGS1	ENSG00000087157	TRUE	0	FALSE	0.967	0.407	13	0.333208383	0.501506057
FALSE	110	rs8071516	DNAH17	ENSG00000187775	TRUE	0	FALSE	0.610	0.062	13	0.438631045	0.313608673
FALSE	110	rs8071516	SOCS3	ENSG00000184557	FALSE	64725	FALSE	0.106	-0.236	13	0.08088878	0.035097495
FALSE	110	rs8071516	TMEM235	ENSG00000204278	FALSE	183815	FALSE	0.652	0.085	13	0.015093458	0.020102629
FALSE	110	rs8071516	BIRC5	ENSG00000089685	FALSE	199166	FALSE	0.262	-0.124	13	0.011965625	0.006103229
FALSE	110	rs8071516	AFMID	ENSG00000183077	FALSE	217100	FALSE	0.610	0.061	13	0.01124989	0.006103229
FALSE	110	rs8071516	TK1	ENSG00000167900	FALSE	237569	FALSE	0.406	-0.047	13	0.013373696	0.006103229
FALSE	110	rs8071516	CYTH1	ENSG00000108669	FALSE	249247	FALSE	0.627	0.072	13	0.012565309	0.007267059
FALSE	110	rs8071516	SYNGR2	ENSG00000108639	FALSE	251275	FALSE	0.950	0.359	13	0.00955575	0.006103229
FALSE	110	rs8071516	C17orf99	ENSG00000187997	FALSE	258505	FALSE	0.471	-0.013	13	0.010497649	0.006050611
FALSE	110	rs8071516	TMC8	ENSG00000167895	FALSE	281834	FALSE	0.462	-0.018	13	0.010208642	0.006050611
FALSE	110	rs8071516	TMC6	ENSG00000141524	FALSE	292395	FALSE	0.733	0.135	13	0.010163678	0.006050611
FALSE	110	rs8071516	TNRC6C	ENSG00000078687	FALSE	315980	TRUE	0.977	0.453	13	0.009352093	0.010491019
FALSE	111	rs8091977	ASXL3	ENSG00000141431	TRUE	9391	FALSE	0.361	-0.072	3	0.849568784	0.733563
FALSE	111	rs8091977	NOL4	ENSG00000101746	FALSE	90514	TRUE	0.954	0.368	3	0.057453863	0.10247218
FALSE	111	rs8091977	CCDC178	ENSG00000166960	FALSE	319485	FALSE	0.136	-0.205	3	0.040830996	0.025730142
TRUE	112	rs12456492	RIT2	ENSG00000152214	TRUE	0	TRUE	1.000	1.369	2	0.89265722	0.95866966
FALSE	112	rs12456492	SYT4	ENSG00000132872	FALSE	173880	FALSE	0.999	1.052	2	0.064867303	0.013575859
FALSE	113	rs55818311	SPPL2B	ENSG00000005206	TRUE	0	FALSE	NA	NA	20	0.450160146	
FALSE	113	rs55818311	ENSG00000273734	ENSG00000273734	FALSE	320	FALSE	NA	NA	20		
FALSE	113	rs55818311	LSM7	ENSG00000130332	FALSE	12880	FALSE	0.936	0.327	20	0.14689602	0.398439661

FALSE	113	rs55818311	TMPRSS9	ENSG00000178297	FALSE	18746	FALSE	0.631	0.074		20	0.053423002	0.087233318
FALSE	113	rs55818311	LINGO3	ENSG00000220008	FALSE	33337	FALSE	0.527	0.017		20	0.01755047	0.026259437
FALSE	113	rs55818311	PEAK3	ENSG00000188305	FALSE	59316	FALSE	0.194	-0.163		20	0.014640792	
FALSE	113	rs55818311	OAZ1	ENSG00000104904	FALSE	68001	FALSE	0.864	0.233		20	0.010773341	0.020895136
FALSE	113	rs55818311	JSRP1	ENSG00000167476	FALSE	71732	FALSE	0.976	0.447		20	0.010744971	0.006843916
FALSE	113	rs55818311	TIMM13	ENSG00000099800	FALSE	84133	FALSE	0.836	0.208		20	0.006949114	0.01075858
FALSE	113	rs55818311	LMNB2	ENSG00000176619	FALSE	86146	FALSE	0.652	0.085		20	0.015548516	0.007405729
FALSE	113	rs55818311	AMH	ENSG00000104899	FALSE	89418	FALSE	0.615	0.064		20	0.014077955	0.006843916
FALSE	113	rs55818311	SF3A2	ENSG00000104897	FALSE	92836	FALSE	0.651	0.084		20	0.00954839	0.009876279
FALSE	113	rs55818311	PLEKHJ1	ENSG00000104886	FALSE	103787	FALSE	0.698	0.111		20	0.008774271	0.009876279
FALSE	113	rs55818311	DOT1L	ENSG00000104885	FALSE	108913	FALSE	0.952	0.363		20	0.01007726	0.009876279
FALSE	113	rs55818311	GADD45B	ENSG00000099860	FALSE	134630	FALSE	0.375	-0.065		20	0.009873826	0.006859024
FALSE	113	rs55818311	GNG7	ENSG00000176533	FALSE	169727	FALSE	0.950	0.358		20	0.007561666	0.009588219
FALSE	113	rs55818311	AP3D1	ENSG00000065000	FALSE	177023	TRUE	0.998	0.894		20	0.007654348	0.011333798
FALSE	113	rs55818311	IZUMO4	ENSG00000099840	FALSE	241898	FALSE	0.881	0.249		20	0.007240197	0.007371431
FALSE	113	rs55818311	MOB3A	ENSG00000172081	FALSE	244818	FALSE	0.413	-0.043		20	0.00632151	0.008839702
FALSE	113	rs55818311	MKNK2	ENSG00000099875	FALSE	290247	FALSE	0.962	0.391		20	0.004955833	0.007057799
FALSE	114	rs2295545	DDRGK1	ENSG00000198171	TRUE	4861	FALSE	0.939	0.332		13	0.211123824	
FALSE	114	rs2295545	LZTS3	ENSG00000088899	FALSE	11940	FALSE	0.731	0.133		13	0.477866173	
FALSE	114	rs2295545	ITPA	ENSG00000125877	FALSE	23379	FALSE	0.841	0.212		13	0.12110167	
FALSE	114	rs2295545	UBOX5	ENSG00000185019	FALSE	25293	FALSE	0.802	0.183		13	0.02425714	
FALSE	114	rs2295545	FASTKD5	ENSG00000215251	FALSE	25624	FALSE	0.308	-0.099		13	0.020302901	
FALSE	114	rs2295545	SLC4A11	ENSG00000088836	FALSE	41928	FALSE	0.534	0.021		13	0.017939115	
FALSE	114	rs2295545	DNAAF9	ENSG00000088854	FALSE	63817	FALSE	0.836	0.208		13	0.010060025	
FALSE	114	rs2295545	AVP	ENSG00000101200	FALSE	100765	FALSE	0.352	-0.076		13	0.014231381	
FALSE	114	rs2295545	OXT	ENSG00000101405	FALSE	112972	FALSE	0.356	-0.074		13	0.014164827	
FALSE	114	rs2295545	MRPS26	ENSG00000125901	FALSE	137239	FALSE	0.878	0.246		13	0.007645635	
FALSE	114	rs2295545	GNRH2	ENSG00000125787	FALSE	139742	FALSE	0.091	-0.253		13	0.012070815	
FALSE	114	rs2295545	PTPRA	ENSG00000132670	FALSE	146413	FALSE	0.851	0.221		13	0.007663764	
FALSE	114	rs2295545	ATRN	ENSG00000088812	FALSE	285530	TRUE	0.966	0.404		13	0.005165942	
TRUE	115	rs111593423	CRLS1	ENSG00000088766	TRUE	0	TRUE	0.985	0.514	0.04854053	8	0.739086311	0.741284124
FALSE	115	rs111593423	ENSG00000286235	ENSG00000286235	TRUE	0	FALSE	NA	NA		8		
FALSE	115	rs111593423	LRRN4	ENSG00000125872	FALSE	17853	FALSE	0.379	-0.062		8	0.095649504	0.075300251
FALSE	115	rs111593423	MCM8	ENSG00000125885	FALSE	23716	FALSE	0.574	0.042		8	0.056318347	0.079419229
FALSE	115	rs111593423	FERMT1	ENSG00000101311	FALSE	52153	FALSE	0.488	-0.004		8	0.042070207	0.006641644
FALSE	115	rs111593423	TRMT6	ENSG00000089195	FALSE	72135	FALSE	0.581	0.046		8	0.027388213	0.036620276

FALSE	115	rs111593423	CHGB	ENSG00000089199	FALSE	97340	FALSE	0.740	0.139		8	0.026167912	0.011607951
FALSE	115	rs111593423	SHLD1	ENSG00000171984	FALSE	158298	FALSE	0.525	0.016		8	0.008426052	
FALSE	116	rs1413146	SULF2	ENSG00000196562	TRUE	0	FALSE	0.898	0.271		2		
FALSE	116	rs1413146	NCOA3	ENSG00000124151	FALSE	120638	TRUE	0.976	0.451		2		
TRUE	117	rs11701722	DYRK1A	ENSG00000157540	TRUE	0	TRUE	0.995	0.701		5	0.581327915	0.8643034
FALSE	117	rs11701722	AP001421.1	ENSG00000267937	FALSE	66636	FALSE	NA	NA		5		
FALSE	117	rs11701722	KCNJ6	ENSG00000157542	FALSE	157571	FALSE	0.753	0.147		5	0.017004684	0.013729562
FALSE	117	rs11701722	VPS26C	ENSG00000157538	FALSE	181884	FALSE	0.815	0.192		5	0.019873863	
FALSE	117	rs11701722	TTC3	ENSG00000182670	FALSE	246691	FALSE	0.892	0.264		5	0.020222548	0.01854176
TRUE	118	rs2837424	DSCAM	ENSG00000171587	TRUE	0	TRUE	1.000	1.728		3		
FALSE	118	rs2837424	PCP4	ENSG00000183036	FALSE	367890	FALSE	0.143	-0.199		3		
FALSE	118	rs2837424	IGSF5	ENSG00000183067	FALSE	495204	FALSE	0.375	-0.065		3		
TRUE	119	rs9611522	EP300	ENSG00000100393	TRUE	0	TRUE	0.998	0.877	0.013772335	19		
FALSE	119	rs9611522	L3MBTL2	ENSG00000100395	FALSE	107403	FALSE	0.463	-0.017		19		
FALSE	119	rs9611522	RBX1	ENSG00000100387	FALSE	124570	FALSE	0.867	0.235		19		
FALSE	119	rs9611522	CHADL	ENSG00000100399	FALSE	131631	FALSE	0.962	0.393		19		
FALSE	119	rs9611522	RANGAP1	ENSG00000100401	FALSE	146900	FALSE	0.818	0.194		19		
FALSE	119	rs9611522	XPNPEP3	ENSG00000196236	FALSE	165064	FALSE	0.490	-0.003		19		
FALSE	119	rs9611522	ZC3H7B	ENSG00000100403	FALSE	203646	FALSE	0.813	0.190		19		
FALSE	119	rs9611522	DNAJB7	ENSG00000172404	FALSE	235766	FALSE	0.532	0.020		19		
FALSE	119	rs9611522	ST13	ENSG00000100380	FALSE	241240	FALSE	0.561	0.035		19		
FALSE	119	rs9611522	TEF	ENSG00000167074	FALSE	269454	FALSE	0.831	0.203		19		
FALSE	119	rs9611522	SLC25A17	ENSG00000100372	FALSE	278480	FALSE	0.501	0.003		19		
FALSE	119	rs9611522	TOB2	ENSG00000183864	FALSE	335615	FALSE	0.763	0.155		19		
FALSE	119	rs9611522	ACO2	ENSG00000100412	FALSE	349951	FALSE	0.702	0.113		19		
FALSE	119	rs9611522	PHF5A	ENSG00000100410	FALSE	361838	FALSE	0.378	-0.063		19		
FALSE	119	rs9611522	POLR3H	ENSG00000100413	FALSE	427920	FALSE	0.742	0.140		19		
FALSE	119	rs9611522	CSDC2	ENSG00000172346	FALSE	463131	FALSE	0.687	0.105		19		
FALSE	119	rs9611522	PMM1	ENSG00000100417	FALSE	479021	FALSE	0.845	0.215		19		
FALSE	119	rs9611522	DESI1	ENSG00000100418	FALSE	500149	FALSE	0.913	0.291		19		
FALSE	119	rs9611522	XRCC6	ENSG00000196419	FALSE	523284	FALSE	0.868	0.236		19		
FALSE	120	rs10775809	PPP6R2	ENSG00000100239	TRUE	0	FALSE	0.859	0.229		28		
FALSE	120	rs10775809	DENND6B	ENSG00000205593	FALSE	39990	FALSE	0.843	0.214		28		
FALSE	120	rs10775809	PLXNB2	ENSG00000196576	FALSE	59356	FALSE	0.970	0.421		28		
FALSE	120	rs10775809	SBF1	ENSG00000100241	FALSE	76217	FALSE	0.920	0.301		28		
FALSE	120	rs10775809	MAPK11	ENSG00000185386	FALSE	96235	FALSE	0.885	0.254		28		

FALSE	120	rs10775809	MAPK12	ENSG00000188130	FALSE	105286	FALSE	0.787	0.172		28
FALSE	120	rs10775809	ADM2	ENSG00000128165	FALSE	114541	FALSE	0.431	-0.035		28
FALSE	120	rs10775809	HDAC10	ENSG00000100429	FALSE	115597	FALSE	0.796	0.178		28
FALSE	120	rs10775809	MIOX	ENSG00000100253	FALSE	119782	FALSE	0.586	0.048		28
FALSE	120	rs10775809	TUBGCP6	ENSG00000128159	FALSE	121979	FALSE	0.935	0.326		28
FALSE	120	rs10775809	LMF2	ENSG00000100258	FALSE	135947	FALSE	0.815	0.192		28
FALSE	120	rs10775809	NCAPH2	ENSG00000025770	FALSE	141222	FALSE	0.431	-0.035		28
FALSE	120	rs10775809	SELENOO	ENSG00000073169	FALSE	149386	FALSE	0.512	0.009		28
FALSE	120	rs10775809	SCO2	ENSG00000284194	FALSE	156566	FALSE	NA	NA		28
FALSE	120	rs10775809	TYMP	ENSG00000025708	FALSE	158750	FALSE	0.645	0.081		28
FALSE	120	rs10775809	ODF3B	ENSG00000177989	FALSE	162708	FALSE	0.651	0.084		28
FALSE	120	rs10775809	TRABD	ENSG00000170638	FALSE	167404	FALSE	0.738	0.138		28
FALSE	120	rs10775809	KLHDC7B	ENSG00000130487	FALSE	178897	FALSE	0.325	-0.090		28
FALSE	120	rs10775809	SYCE3	ENSG00000217442	FALSE	184110	FALSE	0.456	-0.022		28
FALSE	120	rs10775809	PANX2	ENSG00000073150	FALSE	186707	FALSE	0.689	0.106		28
FALSE	120	rs10775809	CPT1B	ENSG00000205560	FALSE	201859	FALSE	0.693	0.108		28
FALSE	120	rs10775809	CHKB-CPT1B	ENSG00000254413	FALSE	201867	FALSE	NA	NA		28
FALSE	120	rs10775809	MOV10L1	ENSG00000073146	FALSE	205312	FALSE	0.714	0.121		28
FALSE	120	rs10775809	CHKB	ENSG00000100288	FALSE	211957	FALSE	0.591	0.051		28
FALSE	120	rs10775809	MAPK8IP2	ENSG00000008735	FALSE	233791	FALSE	0.920	0.302		28
FALSE	120	rs10775809	ARSA	ENSG00000100299	FALSE	255751	FALSE	0.979	0.466		28
FALSE	120	rs10775809	MLC1	ENSG00000100427	FALSE	281576	TRUE	0.981	0.478		28
FALSE	120	rs10775809	TTLL8	ENSG00000138892	FALSE	308704	FALSE	0.097	-0.247		28

Prioritized = Boolean value indicating whether the gene was prioritized based on our criteria (distance to the credible set, PoPS value, and presence of non-synonymous variant(s) in the credible set; Locus = locus number; rsID = GWAS lead SNP for this locus; Gene = HUGO Gene Nomenclature Committee gene symbol; ENSGID = ENSEMBL gene ID; BestDistance = Distance (basepairs) between credible set and the prioritized gene (position weighted by posterior inclusion probability); Distance = Distance (basepairs) between credible set and the prioritized gene (position weighted by posterior inclusion probability); BestPoPS = Boolean value indicating whether the gene has the highest PoPS score in the locus; PoPS quantile = PoPS score quantile; PoPS value = PoPS score raw; Non-synonymous PIP = Summed posterior inclusion probability of non-synonymous variants in the credible set; # genes = Number of genes in the locus; L2G = Probability that this gene is causal in this GWAS locus, predicted by the L2G model (Mountjoy et al. 2021); Yu2024 = Probability that a this gene is causal in this GWAS locus, predicted by the Yu2024 model (Yu et al. 2024). If the sum of causal probabilities exceeded 100%, we rescaled them to sum to 100%.

Supplementary Table 3. Prioritized genes targeted by approved and investigational drugs.

**Gene**	**Example approved drugs (indications)**	**Example investigational drugs (indications, phase)**
SNCA	None	Cinpanemab (Parkinson's disease, Phase II, terminated)<br>Prasinezumab (Parkinson's disease, Phase II)<br>Anle138b (Parkinson's disease, Phase I; completed)
LRRK2	None	BIIB122 (Parkinson's disease, Phase II)<br>NEU-411 (Parkinson's disease, Phase II)
GBA1	Ambroxol (Respiratory diseases)	Ambroxol (Parkinson's disease, Phase II)<br>Imiglucerase [Cerezyme®] (Gaucher disease, Phase III + IV, completed)<br>Abcertin (Gaucher disease, Phase I; completed)<br>LY3884961 (Parkinson's disease and Gaucher disease Type 2, Phase I/IIa)<br>PEG-Glucocerebrosidase (Lysodase) (Gaucher disease, Phase I, completed)<br>Velaglucerase alfa [gene-activated human glucocerebrosidase, VPRIV] (Gaucher disease Type 1, Phase III, completed)<br>Glucocerebrosidase (Gaucher disease, Phase II + III)<br>Taliglucerase alfa [Plant Cell Expressed Recombinant Human Glucocerebrosidase, prGCD] (Gaucher disease, Phase III, completed)<br>Eliglustat tartrate [Genz-112638] (Gaucher disease, Phase II + III, completed)<br>BIA 28-6156 (Parkinson's disease, Phase II)<br>Venglustat GZ/SAR402671 (Parkinson's disease, Phase II, terminated)<br>Afegostat (Gaucher disease, Phase II, completed)
FYN	Dasatinib (Various malignancies)<br>Bosutinib (Philadelphia chromosome-positive chronic myeloid leukemia)<br>Ponatibin (Philadelphia chromosome-positive chronic myeloid leukemia)	Saracatinib (Alcohol drinking behaviour, various malignancies, Alzheimer's disease, Phase II, completed)<br>Dasatinib (Various malignancies, Phase II - IV)<br>Dasatinib (Accelerated Aging in Mental Disorders, Aging/Individuals at risk for Alzheimer's disease, Mild cognitive impairment, Alzheimer's disease, Phase I + II)<br>JNJ-26483327 (Solid tumors, Phase I)<br>Ponatinib (Acute Lymphoblastic Leukemia, Phase II)<br>TPX-0046 and TPX-0022 (Various malignancies, Phase I-II); AZD0424 (Solid tumors, Phase I)<br>AZD0530 (Non Small Cell Lung Cancer, Epithelial Ovarian Cancer, Phase I)<br>Bosutinib (Breast Cancer, Phase I)<br>KX2-391 (Bone-Metastatic, Prostate Cancer, Phase II)<br>Repotrectinib (Solid Tumors, Phase I + II)

DYRK1A	None	Epigallocatechin-3-gallate [EGCG] (Cognitive deficits in Down syndrome, Phase II)<br>FRTX-02 (Atopic Dermatitis Eczema, Phase 1, terminated)<br>Leucettinib-21 (Down Syndrome and Alzheimer's disease, Phase I)
*CTSB**	Ionafarnib (Hutchinson-Gilford progeria syndrome)<br>Lonafarnib (Hutchinson-Gilford progeria syndrome)<br>Sorafenib (Various malignancies)	Lonafarnib (Various malignancies, Chronic hepatitis D, Phase I/II, most studies completed/terminated)<br>Tipifarnib (Various malignancies, Phase I/II, most studies completed/terminated)<br>Sorafenib (Various malignancies, Phase I/II, most studies completed/terminated)
NOD2	Mifamurtide (Osteosarcoma)	None
SV2C	Padsevonil (Epilepsy)	Padsevonil (Liver disease, Phase I, terminated)
XPO1	Selinexor (Multiple myeloma and diffuse large B-cell lymphoma)	Selinexor (Various malignancies, Phase I-III)<br>KPT-8602 (Various malignancies, Phase I/II)<br>SL-801 (Solid tumors, Phase I)
PIK3CA	Alpelisib (Breast cancer and PIK3CA-related overgrowth spectrum)<br>Idelasinib (Blood cancer)<br>Duvelisib (Leukemia/Lymphoma)<br>Inavolisib (Breast cancer)<br>Copanlisib (Follicular lymphoma)	Multiple PIK3CA inhibitors in Phase II (e.g., Gedatolisib, Voxtalisib) and III (e.g., Buparlisib, Dactolisib) clinical trials for various malignancies

* Note: All farnesyltransferase inhibitors listed here do not target CTSB directly but have effects on the molecular pathway CTSB is involved in.

Column descriptions:
Gene = HUGO Gene Nomenclature Committee gene symbol
Example approved drugs (indications) = Examples of approved drugs for a selection of genes mentioned in the main manuscript based on the information available on the Open Targets Platform (https://platform.opentargets.org/) and ongoing clinical trials (listed on https://clinicaltrials.gov/). Associated indications as well as the clinical trial phase for investigational drugs are displayed in parentheses. Alternative drugs names are indicated in square brackets (if applicable).
Example investigational drugs (indications, phase) = Examples of investigational drugs for a selection of genes mentioned in the main manuscript based on the information available on the Open Targets Platform (https://platform.opentargets.org/) and ongoing clinical trials (listed on https://clinicaltrials.gov/). Associated indications as well as the clinical trial phase for investigational drugs are displayed in parentheses. Alternative drugs names are indicated in square brackets (if applicable).

Supplementary Figure

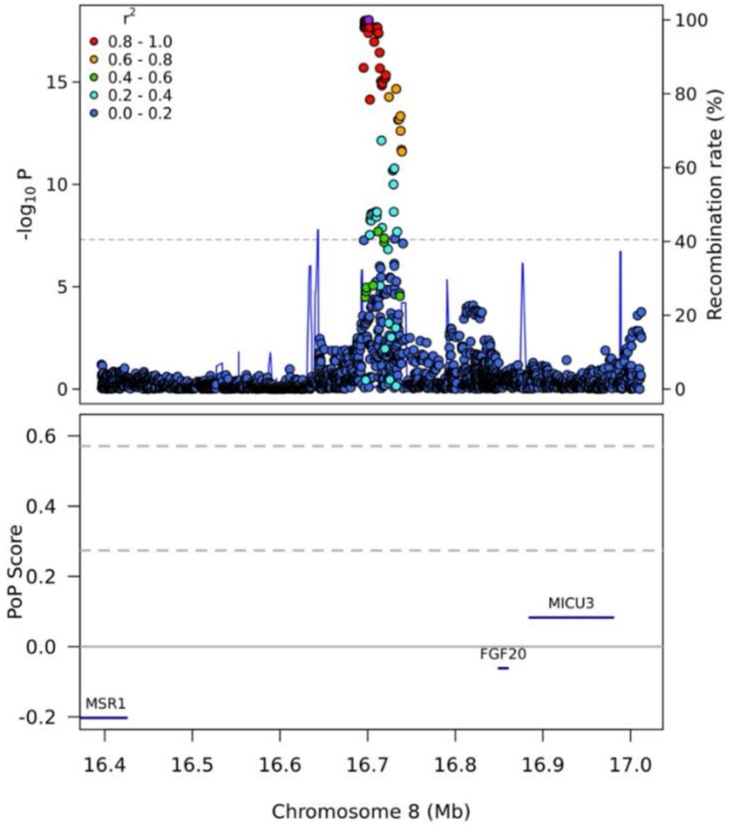

Supplementary Figure 1. Variant-level associations and PoPS results for the *MICU3* locus. The upper portion of each sub-plot is a LocusZoom plot. Each point represents a different genetic variant, the x-axis represents physical position on the listed chromosome, the left y-axis represents $-\log_{10}$-transformed P value, the right y-axis represents the recombination rate, colour represents linkage disequilibrium with the lead variant in the locus (as shown in the legend), and the horizontal dashed line represents the genome-wide significance P value threshold of 5×10^{-8}. The lower portion of each figure is a PoPS plot. Genes are denoted as blue bars spanning from their transcription start site to their transcription stop site using the same x-axis as the LocusZoom plot, the y-axis represents the raw PoPS score, the dashed horizontal grey lines represent the top 10% and 1% of PoPS scores genome-wide, and the solid horizontal grey line represents a PoPS score of 0.
